# Dysregulation of core neurodevelopmental pathways—a common feature of cancers with perineural invasion

**DOI:** 10.3389/fgene.2023.1181775

**Published:** 2023-08-31

**Authors:** Luz María González-Castrillón, Maud Wurmser, Daniel Öhlund, Sara Ivy Wilson

**Affiliations:** ^1^ Department of Integrative Medical Biology, Umeå University, Umeå, Sweden; ^2^ Wallenberg Centre for Molecular Medicine, Department of Radiation Sciences, Umeå University, Umeå, Sweden

**Keywords:** perineural invasion, cancer, neurodevelopment, bioinformatics, biomarker, head and neck squamous cell carcinoma, pancreatic ductal adenocarcinoma, stomach adenocarcinoma

## Abstract

**Background:** High nerve density in tumors and metastasis via nerves (perineural invasion—PNI) have been reported extensively in solid tumors throughout the body including pancreatic, head and neck, gastric, prostate, breast, and colorectal cancers. Ablation of tumor nerves results in improved disease outcomes, suggesting that blocking nerve–tumor communication could be a novel treatment strategy. However, the molecular mechanisms underlying this remain poorly understood. Thus, the aim here was to identify molecular pathways underlying nerve–tumor crosstalk and to determine common molecular features between PNI-associated cancers.

**Results:** Analysis of head and neck (HNSCC), pancreatic, and gastric (STAD) cancer Gene Expression Omnibus datasets was used to identify differentially expressed genes (DEGs). This revealed extracellular matrix components as highly dysregulated. To enrich for pathways associated with PNI, genes previously correlated with PNI in STAD and in 2 HNSCC studies where tumor samples were segregated by PNI status were analyzed. Neurodevelopmental genes were found to be enriched with PNI. In datasets where tumor samples were not segregated by PNI, neurodevelopmental pathways accounted for 12%–16% of the DEGs. Further dysregulation of axon guidance genes was common to all cancers analyzed. By examining paralog genes, a clear pattern emerged where at least one family member from several axon guidance pathways was affected in all cancers examined. Overall 17 different axon guidance gene families were disrupted, including the ephrin–Eph, semaphorin–neuropilin/plexin, and slit–robo pathways. These findings were validated using The Cancer Genome Atlas and cross-referenced to other cancers with a high incidence of PNI including colon, cholangiocarcinoma, prostate, and breast cancers. Survival analysis revealed that the expression levels of neurodevelopmental gene families impacted disease survival.

**Conclusion:** These data highlight the importance of the tumor as a source of signals for neural tropism and neural plasticity as a common feature of cancer. The analysis supports the hypothesis that dysregulation of neurodevelopmental programs is a common feature associated with PNI. Furthermore, the data suggested that different cancers may have evolved to employ alternative genetic strategies to disrupt the same pathways*.* Overall, these findings provide potential druggable targets for novel therapies of cancer management and provide multi-cancer molecular biomarkers.

## 1 Introduction

Cancer is one of the leading causes of death worldwide (Ferlay et al., 2021). Discovery of new and better ways to detect and treat cancer therefore remains an urgent global health challenge. During tumor establishment, several features important for the organs’ normal function and homeostasis are hijacked by the tumor to facilitate its growth and progression. This includes molecular and cellular changes in the surrounding non-cancerous cells, changes in the extracellular matrix, and growth and remodeling of the vasculature (Hanahan, 2022).

In addition to the vasculature, tumors are invaded by nerves, a phenomenon first documented over 100 years ago (Young, 1897). It has long been observed that at a histopathological level, tumor nerves are used as a route for metastasis, through a process referred to as perineural invasion (PNI) (Liebig et al., 2009). Together, high nerve density and PNI are histopathological features of a wide range of solid tumors throughout the body, including pancreas, colon, bile duct, gastric, head and neck, prostate, breast, skin tumors and others (Amit et al., 2016; Schmitd et al., 2018; Chen et al., 2019; Eviston et al., 2021; Yang et al., 2021). These disease features have also been widely correlated with more aggressive disease outcomes and are associated with disease stage in a number of cancers (Cui et al., 2015; Blumenthaler et al., 2021; Lee et al., 2021; Li et al., 2022). Importantly, studies using a number of cancer animal models have revealed that chemical or surgical ablation of nerves supplying tumors results in improved disease outcomes such as delays in preneoplastic lesions, reduction in tumor growth, invasion, and metastasis (Zhao et al., 2014; Zahalka and Frenette, 2020). Supporting this, vagotomy (vagus nerve resection) during gastric cancer surgery in patients resulted in better disease outcomes (Zhao et al., 2014). Taken together, this evidence supports the notion that communication between the organs’ endogenous nerves and the tumor microenvironment has an important pathophysiological role in tumor development and progression and therefore provides a novel therapeutic target for disease management. Despite the pathophysiological importance of nerve–tumor interactions, the molecular pathways underlying high nerve density in tumors and subsequent PNI remain poorly understood. This is thought to be a multistep process involving nerve signaling, activation of neural growth, and branching, in addition to tropism and migration of the tumor cells to the nerve, invasion of the nerve by the tumor cells, and metastasis via the nerves. Through those processes, both the tumor cells and neural and glial cells of the growing nerve must navigate through complex tissue environments.

In healthy adults, growth and reorganization of endogenous nerves occurs to a limited extent, at both structural and functional levels via neural plasticity. This includes synaptic remodeling, branching, and growth of existing nerves. While neural plasticity of established nerves occurs in healthy adults, most neural growth, remodeling, and innervation occur during embryonic and postnatal development. Both the growth and development of nerves during embryogenesis and neural plasticity are directed by distinct molecular pathways and sculpted neural activity (Cooper, 2002; Kolodkin and Tessier-Lavigne, 2011; Stoeckli, 2018). This includes axon guidance pathways which are composed of a wide array of molecules and processes such as groups of ligand/receptor families; growth factors such as neurotropic factors and mitogens; chemokines; and classical axon guidance molecules such as Slit/Robo, Eph/Ephrin, and Netrin/DCC/Unc pathways among other molecular classes such as transcription factors, intracellular signaling, adhesion molecules, extracellular matrix components, and neural activity (Cho and Miller, 2002; Butler and Tear, 2007; Li and Ransohoff, 2008; Kolodkin and Tessier-Lavigne, 2011; Stoeckli, 2018).

A number of classes of neurodevelopmental molecules have been reported as significantly dysregulated in a wide range of cancers including neurotrophic factors, chemokines, transcription factors, classical axon guidance molecules, extracellular matrix components, and adhesion molecules amongst other molecules (Duman-Scheel, 2009; Biankin et al., 2012; Pickup et al., 2014; Ashrafizadeh et al., 2020; Brotto et al., 2020). Furthermore, studies on several cancers have identified a number of these molecules in the processes of either tumor nerve growth or PNI (Liebig et al., 2009; Bapat et al., 2011; Amit et al., 2016; Liang et al., 2016; Liu et al., 2022). For instance, neurotrophic molecules such as NGF, BDNF, and GDNF have been found overexpressed in tumors from the pancreas and prostate and can promote nerve growth (Liu et al., 2022). SLIT ligands and their cognate receptors ROBOs have been observed in pancreatic adenocarcinoma (PDAC), leading to more aggressive disease and can modulate tumor nerve growth (Biankin et al., 2012; Göhrig et al., 2014). In a similar way, dysregulation of the classical axon guidance ligands ephrins, semaphorins, and their cognate receptors Ephs and neuropilins/plexins, respectively, have been noted in a wide range of cancers, and in some individual cases, expression of these genes in tumor cells has been found to modulate nerve growth in *in vitro* assays (Capparuccia and Tamagnone, 2009; Pasquale, 2010).

Overall, a clear pattern is starting to emerge where a number of key molecules important during normal neurodevelopment have been found to be dysregulated in tumors and in some cases, associated with tumor nerve growth and PNI in a broad range of cancer types (Capparuccia and Tamagnone, 2009; Pasquale, 2010; Liu et al., 2022). This has led to the hypothesis examined here that tumor nerve growth and PNI could be a reactivation of the molecular pathways normally driving nerve growth, guidance, and cell migration during embryonic and postnatal development. Furthermore, we hypothesize that a broad range of cancers could share common molecular features to achieve this. Discovery of pathways which underlie nerve–tumor interaction could lead to treatment strategies which target this process. However, the current literature has shown that the degree of dysregulation and phenotypic role of each molecule in tumor nerve growth and PNI varies for individual molecules and cancer types. Therefore, while individual studies have brought insight into individual molecules and cancers, whether different cancer types use common molecular pathways to promote tumor nerve growth and PNI remains largely unexplored. The study here, therefore, provides a molecular analysis across several cancers associated with a high incidence of tumor nerve growth and PNI by examining the broad landscape of neurodevelopmental molecular programs. Bioinformatics analysis of Gene Expression Omnibus (GEO) and The Cancer Genome Atlas (TCGA) datasets revealed a number of neurodevelopmental programs including axon guidance genes dysregulated across all cancers analyzed. Furthermore, a clear pattern has emerged that different cancers have evolved to disrupt the same molecular pathways, albeit by dysregulation of differing paralog genes. Overall, these data support the hypothesis that dysregulation of neurodevelopmental programs known to be fundamental to the growth and guidance of neurons during normal development and involved in neural plasticity is a common feature among the cancers analyzed.

## 2 Results

### 2.1 Study population and examination of the GEO dataset characteristics

**FIGURE 1:**
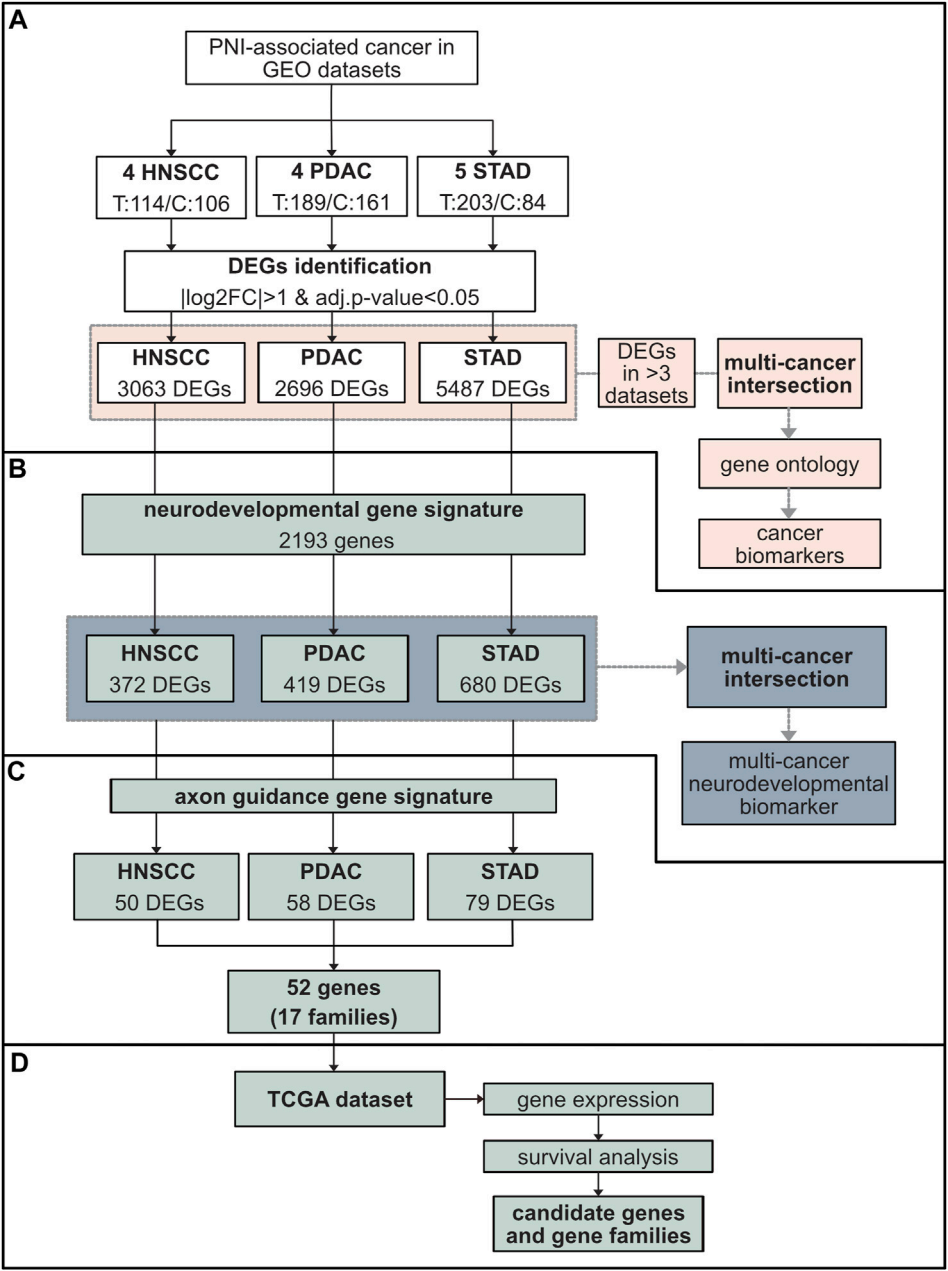
Schematic representation of the study design and data analysis. **(A)** Study population comprising GEO cohorts from three cancers associated with high nerve density and PNI, HNSCC (four datasets), PDAC (four datasets), and STAD (five datasets) (Table 1). The total number of tumor (T) versus control **(C)** samples are indicated. The fold change in gene expression between tumor and control tissue was performed through the software GEO2R. Genes with |log2FC|>1 and adj. p-val <0.05 were considered differentially expressed genes (DEGs). The number of DEGs for each cancer is indicated. For each cancer type, DEGs common in at least three datasets from the same cancer type were identified (pink). An intersection between the DEGs common in at least three datasets for each cancer were subsequently cross-referenced between PDAC, HNSCC, and STAD to identify common genes and gene ontology performed (pink). **(B)** The DEGs identified in **(A)** were cross-referenced with a neurodevelopmental gene signature (QuickGO GO:0007399) (green). A total of 372 neurodevelopmental DEGs were isolated in HNSCC, 419 in PDAC, and 680 in STAD. Neurodevelopmental DEGs were intersected among all cancers analyzed (green). **(C)** DEGs from each cancer type were analyzed for the presence of axon guidance genes. This resulted in 52 individual axon guidance genes across cancers comprising 17 common axon guidance gene families dysregulated in all cancers. **(D)** These results were further analyzed using TCGA data cohorts including the same cancers to the GEO analysis in addition to other cancer types associated with high nerve density and PNI: BRCA, PRCA, CHOL, and COAD. This analysis included gene expression comparison between tumor and control tissue and survival analysis. Abbreviations: GEO, Gene Expression Omnibus database; HNSCC, head and neck squamous cell carcinoma; PDAC, pancreatic ductal adenocarcinoma; STAD, stomach adenocarcinoma; DEG, differentially expressed gene; FC, fold change; TCGA, The Cancer Genome Atlas; BRCA, breast cancer; PRCA, prostate cancer; CHOL, cholangiocarcinoma; COAD, colon adenocarcinoma.

The goal of the study was to identify molecular pathways underlying nerve plasticity/growth in response to the tumor microenvironment and subsequent PNI. To this end, microarray datasets of solid tumors outside the nervous system associated with high nerve density and PNI were identified in the GEO database. While a number of cancers have been reported to have a high incidence of PNI, in this study, we selected cancers where several high-quality GEO datasets were available. This included a total of 13 datasets from head and neck squamous cell carcinoma (HNSCC), pancreatic ductal adenocarcinoma (PDAC), and stomach adenocarcinoma (STAD) datasets (Figure 1A; Table1 and Supplementary Figures S1–S14). The quality and characteristics of the 13 selected datasets were first analyzed using expression density and box plots (Supplementary Figures S1A, B–S13A, B). This analysis showed that the datasets were appropriate for differential expression analysis given their median center distribution. In addition to this and given that five datasets had an unbalanced tumor/control sample composition (HNSCC-GSE23036 and GSE31056, PDAC-GSE16515, GC-GSE33651, and GSE54129), a group composition equalization analysis was performed. This equalization analysis revealed that a large number of differentially expressed genes (DEGs) for each unbalanced datasets overlapped with the DEGs identified in the datasets with a balanced sample composition (Supplementary Figures S1–S5). This demonstrated that the datasets with unbalanced composition were suitable to include in the analysis. Next, all datasets were analyzed by unsupervised clustering to determine if samples clustered into tumor and control groups. Approximately half the datasets broadly clustered into tumor and control groups, while others did not (Supplementary Figures S1–S13). Of note, datasets where disease-free adjacent control tissue was used clustered less well than those using contralateral or healthy donor as control tissue (Supplementary Figures S1–S13). Taken together, these data showed that the 13 GEO datasets representing PDAC, STAD, and HNSCC consisted of good-quality data cohorts with some caveats.

**TABLE 1:** GEO dataset study population characteristics. The GEO microarray datasets included in this study are indicated. Of those which met the quality criteria, four datasets were available for HNSCC, four for PDAC, and five for gastric cancer (referred to as STAD in the manuscript). Samples comprised tumor and adjacent tissue or healthy biopsy. For the analysis of the dataset GSE138206, control samples consisted of contralateral normal samples; in this study the tissue adjacent to cancer was excluded. Abbreviations: GEO, Gene Expression Omnibus database; HNSCC, head and neck squamous cell carcinoma; PDAC, pancreatic ductal adenocarcinoma; STAD, stomach adenocarcinoma; GC, gastric cancer; OSCC, oral squamous cell carcinoma.

Cancer type	Dataset	Tumor/normal	Tumor/normal type	Platform (# probes)	Author, year
Head and neck squamous cell carcinoma cancer tumor: 114/control: 106	GSE138206	6/6	OSCC tumor/contralateral normal	GPL570 (54,675)	Pan H. et al., 2019
	GSE23036	63/5	Pre-treatment HNSCC biopsy/healthy tissue	GPL571 (22,277)	Pavon et al. (2012)
	GSE31056	23/73	OSCC tumor/adjacent tissue	GPL10526 (17,788)	Reis et al. (2011)
	GSE6631	22/22	HNSCC tumor/adjacent tissue or contralateral normal	GPL8300 (12,625)	Kuriakose et al. (2004)
Pancreatic ductal adenocarcinoma tumor: 189/control: 161	GSE15471	39/39	PDAC tumor/adjacent tissue	GPL570 (54,675)	Badea et al. (2008)
	GSE16515	36/16	Pancreatic tumor/adjacent tissue	GPL570 (54,675)	Pei et al. (2009)
	GSE28735	45/45	PDAC tumor/adjacent tissue	GPL6244 (33,297)	Zhang et al. (2012)
	GSE62452	69/61	PDAC tumor/adjacent tissue	GPL6244 (33,297)	Yang et al. (2016)
Gastric cancer tumor: 203/control: 84	GSE103236	10/9	GC tumor/adjacent tissue	GPL4133 (45,220)	Economescu et al. (2010)
	GSE33651	40/12	GC tumor/healthy biopsy	GPL2895 (56,448)	Park et al. (2011)
	GSE54129	111/21	GC tumor/healthy biopsy	GPL570 (54,675)	Lui B. et al., 2014
	GSE65801	32/32	GC tumor/adjacent tissue	GPL14550 (42,545)	Li et al. (2015)
	GSE79973	10/10	GC tumor/adjacent tissue	GPL570 (54,675)	He et al. (2016)

### 2.2 Extracellular matrix remodeling is highly enriched across all cancers examined

**FIGURE 2:**
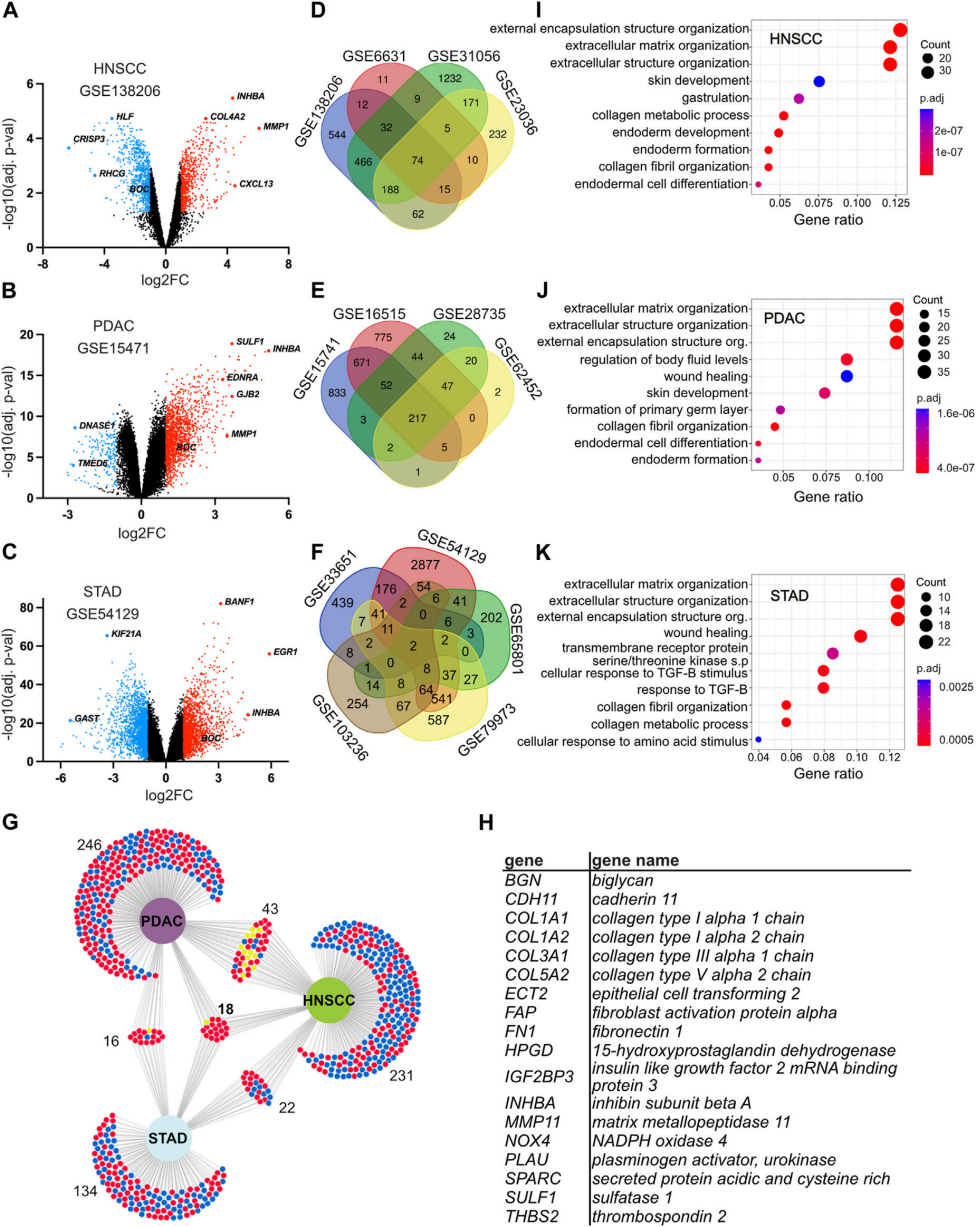
Identification of differentially expressed genes and genes common in head and neck squamous cell carcinoma (HNSCC), pancreatic ductal adenocarcinoma (PDAC), and stomach adenocarcinoma (STAD). **(A–C)** Volcano plots displaying the distribution of upregulated and downregulated genes when comparing gene expression between tumor and control tissues in selected datasets GSE138206 for HNSCC **(A)**, GSE15471 for PDAC **(B),** and GSE54129 for STAD **(C)**. All datasets are shown in Supplementary Figure S14. The *X*-axis indicates the log2FC and the *Y*-axis the −log10 (adj. *p*-value). Each dot represents a gene. Red depicts upregulated genes with log2FC > 1 and adj. *p*-value <0.05; blue depicts downregulated genes with a log2FC < −1 and adj. *p*-value <0.05. Black depicts genes that were either not significant or where the fold change was below the threshold. **(D–F)** Venn diagrams of DEGs in the indicated datasets showing the intersection between different datasets from the same cancer type. Each dataset has a distinct color and is labeled with the dataset identification number for HNSCC **(D)**, PDAC **(E),** and STAD **(F)**. **(G)** Intersection analysis of DEGs between HNSCC, PDAC, and STAD. DEGs common in at least three datasets for the same cancer were intersected between each cancer examined. Significantly downregulated and upregulated DEGs are delineated with blue and red, respectively. Genes that are either down and upregulated depending on the cancer type are depicted in yellow. The number of genes in each dataset is indicated. **(H)** List of the 18 DEGs common in at least three datasets in each cancer. **(I–K)** Gene Ontology analysis of DEGs common in at least three different datasets for the same cancer. Enriched terms for the top 10 biological processes ranked by their count and gene ratio are presented for HNSCC **(I)**, PDAC **(J),** and STAD **(K)**. Abbreviations: DEG, differentially expressed gene; HNSCC, head and neck squamous cell carcinoma; PDAC, pancreatic ductal adenocarcinoma; STAD, stomach adenocarcinoma; s. p, signaling pathway; TGF-B, transforming growth factor beta adj. *p*-value, adjusted *p*-value.

In order to identify candidate genes differentially expressed in PDAC, STAD, and HNSCC, next the limma package in GEO2R was used to compare gene expression values between the tumor and control tissue of GEO datasets. Here, DEGs were defined as having an expression log fold change of more than 2 (|log2FC|>1) and adjusted *p*-value (adj. P. val) <0.05 (Figure 1A, 2A–C, Supplementary Figure S14; Supplementary Table S1). Through this analysis, the combined number of DEGs for all datasets from a particular cancer type was found to be 3,063 for HNSCC, 2,696 for PDAC, and 5,487 for STAD (Figure 1A; Supplementary Table S2).

Next, to identify common pathways and potential biomarkers for HNSCC, PDAC, and STAD, an intersectional analysis between datasets within the same cancer type was performed. This revealed 314 DEGs for HNSCC, 323 for PDAC, and 190 for STAD common in at least three datasets from the same cancer type, of which 18 genes were common to all cancers analyzed (Figures 2A–H; Supplementary Tables S2, S3), which were *BGN*, *CDH11*, *COL1A1*, *COL1A2*, *COL3A1*, *COL5A2*, *ECT2*, *FAP*, *FN1*, *HPGD*, *IGF2BP3*, *INHBA*, *MMP11*, *NOX4*, *PLAU*, *SPARC*, *SULF1*, and *THBS2*. To identify potential dysregulated processes and pathways in PDAC, HNSCC, and STAD, a Gene Ontology (GO) analysis was performed using DEGs common in at least three datasets for each cancer type. From this analysis, cellular components, molecular functions, and biological processes associated with extracellular matrix were highly enriched in all three cancers examined as well as the genes common to all cancers. This included terms for extracellular matrix organization (biological process), external encapsulation structure organization (biological processes), collagen-containing extracellular matrix (cellular component), and extracellular matrix structural constituent (molecular function) (Figures 2I–K, Supplementary Figure S15). Overall, this analysis led to the identification of extracellular matrix components and organization as commonly dysregulated among the cancers analyzed.

### 2.3 Neurodevelopmental genes are highly dysregulated in PDAC, HNSCC, and STAD

**FIGURE 3:**
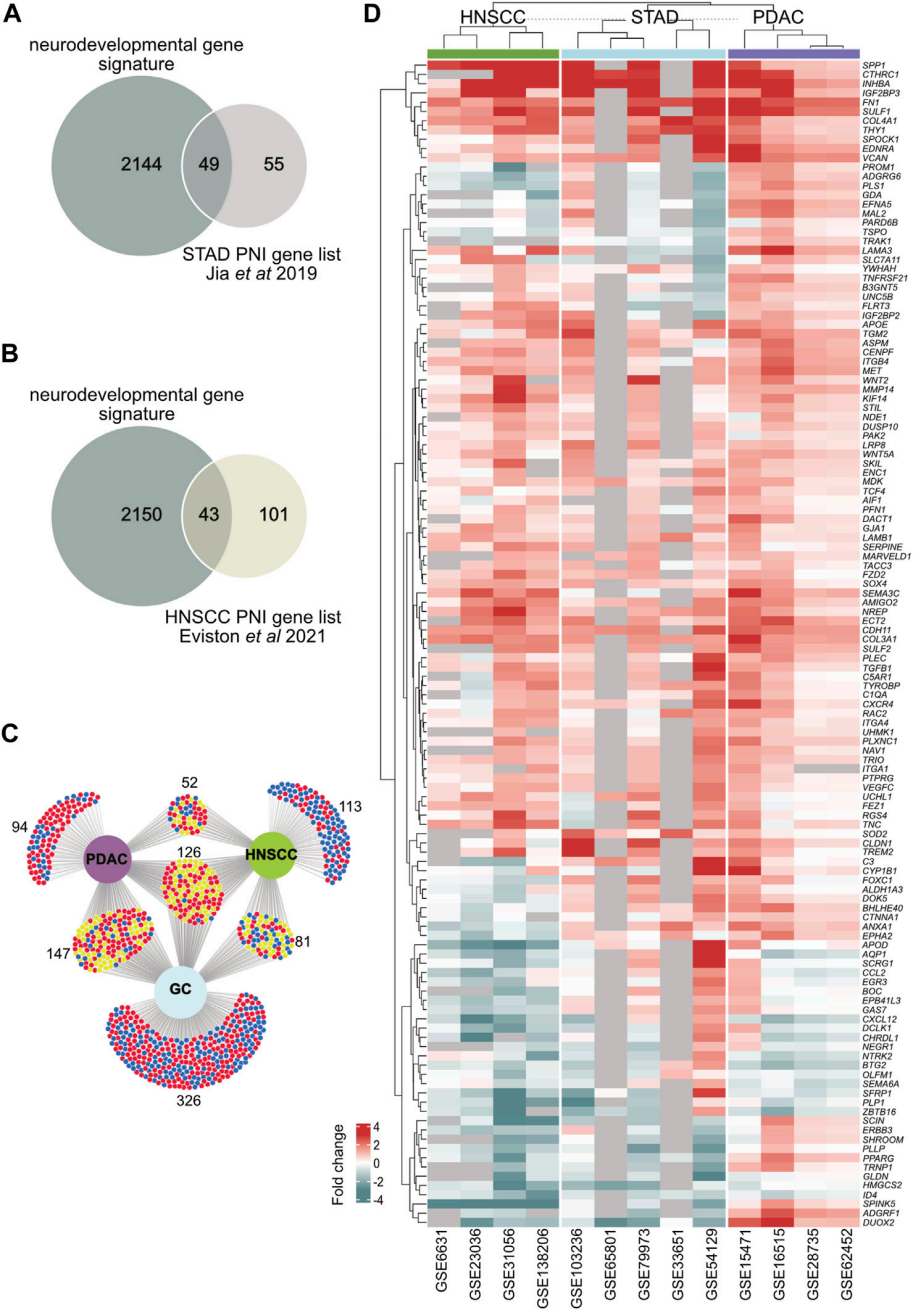
Identification of neurodevelopmental DEGs in head and neck squamous cell carcinoma (HNSCC), pancreatic ductal adenocarcinoma (PDAC), and stomach adenocarcinoma (STAD). **(A)** A Venn diagram showing the intersection analysis between a gastric cancer PNI gene list constructed from Jia et al. (2019) and a neurodevelopmental gene signature in this paper. **(B)** A Venn diagram showing the intersection analysis between a HNSCC PNI gene list constructed from Eviston et al. (2021) and a neurodevelopmental gene signature in this paper. **(C)** DEGs from all cancer datasets were cross-referenced with the neurodevelopmental gene signature. An intersection analysis of neurodevelopmental DEGs from PDAC, HNSCC, and STAD is shown. 372 neurodevelopmental DEGs were found in HNSCC, 419 in PDAC, and 680 in STAD. There were 126 neurodevelopmental DEGs common to all cancers in any dataset from each cancer. Significantly downregulated and upregulated DEGs are delineated with blue and red, respectively. Genes that are either down or upregulated depending on the cancer type are depicted in yellow. **(D)** A hierarchical clustering heatmap of the 126 DEGS common to PDAC, HNSCC and GC in **(C)** is shown. Genes that were not annotated for a given dataset are shown in gray. The expression fold change values are depicted by the intensity of color along a red (upregulated) to blue (downregulated) scale as indicated on the diagram. Abbreviations: DEG, differentially expressed gene; HNSCC, head and neck squamous cell carcinoma; PDAC, pancreatic ductal adenocarcinoma; STAD, stomach adenocarcinoma; PNI, perineural invasion.

The transcriptomic datasets analyzed were derived from bulk tumor samples which are composed of numerous cell types. Therefore, the transcriptome of the tumor samples represents multiple molecular and cellular processes. We, therefore, next designed a strategy to isolate candidate pathways that direct the plasticity/growth of tumor nerves and PNI. A previous study in gastric cancer constructed a list of 104 genes, which had been implicated in PNI in the research literature (referred to here as the “gastric cancer PNI gene list”) (Jia et al., 2019). From this gastric cancer PNI gene list, we noted that many of the genes were associated with the development of the nervous system in the embryo, a period when neurons normally grow. We, therefore, next aimed to ask if disruption of neurodevelopmental programs could be a core feature of nerve/tumor interactions. To test this hypothesis, a neurodevelopmental gene signature consisting of 2,193 genes (GO:0007399) was constructed (Supplementary Figure S16, Supplementary Tables S4–6). Strikingly, cross-referencing this neurodevelopmental gene signature with the gastric cancer PNI gene list revealed that 47% of the genes in the gastric cancer PNI gene list were neurodevelopmental genes (Figure 3A; Supplementary Table S5).

To determine if neurodevelopmental gene dysregulation was associated with PNI in different cancers, datasets where the data had been segregated based on the presence or absence of PNI were analyzed (Supplementary Table S7; Supplementary Figure S17). Among the cancers analyzed, the PDAC GEO dataset GSE102238 (Yang et al., 2020), HNSCC GEO dataset GSE86544 (Warren et al., 2016), and a HNSCC published dataset (referred to here as Eviston dataset) (Eviston et al., 2021) were analyzed. Analysis of the PDAC dataset GSE102238 (Yang et al., 2020) revealed low statistical significance when comparing samples designated as PNI/no PNI tumors (Supplementary Figure S17B). Interestingly, cross-referencing the neurodevelopmental list with the 144 significant HNSCC DEGs from the Eviston dataset revealed that 30% of the genes were neurodevelopmental (Figure 3B). For the HNSCC dataset (GSE86544) (Warren et al., 2016), we identified 11% of the DEGs between PNI/no PNI tumors were neurodevelopmental genes (Supplementary Table S8). These data supported the idea that neurodevelopmental genes were associated with PNI.

Next, to determine the extent to which neurodevelopmental genes were dysregulated in PDAC, HNSCC, and STAD, the neurodevelopmental gene signature was cross-referenced with the DEGs from the PDAC, HNSCC, and STAD datasets derived from bulk tumor samples (Figure 1B, 3C; Supplementary Table S5). By combining all datasets for each individual cancer, this analysis revealed that the 372 neurodevelopmental DEGs identified in HNSCC, 419 in PDAC, and 680 in STAD represented approximately 12%, 16%, and 12% of the total DEGs for HNSCC, PDAC, and STAD, respectively (Figure 1B; Figure 3C; Supplementary Table S5). Intersection of the neurodevelopmental genes identified from PDAC, HNSCC, and STAD revealed 126 neurodevelopment DEGs common in all cancer types examined (Figures 3C, D; Supplementary Table S6). This analysis supported the notion that neurodevelopmental genes were highly dysregulated in the cancers analyzed and supported the notion that neurodevelopmental programs were dysregulated in cancers with a high incidence of PNI.

### 2.4 Common axon guidance pathways dysregulated in PDAC, HNSCC, and STAD

**FIGURE 4:**
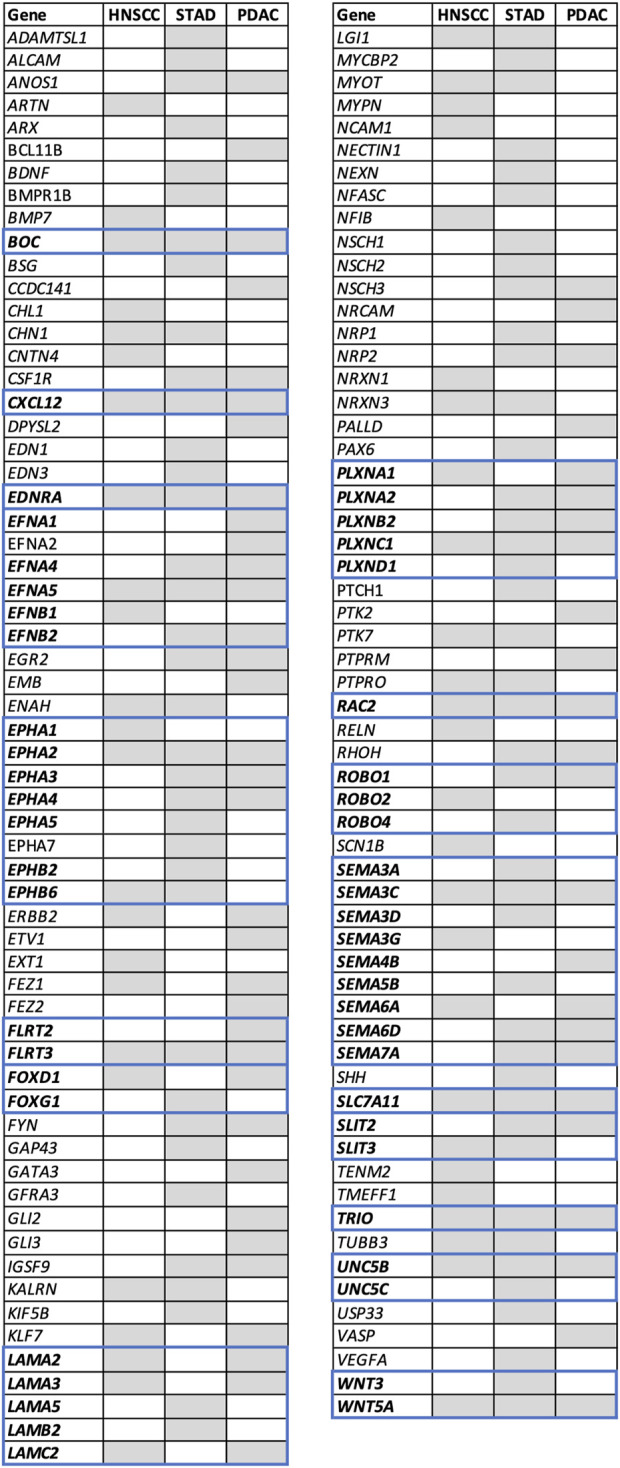
Axon guidance differentially expressed genes in head and neck squamous cell carcinoma (HNSCC), pancreatic ductal adenocarcinoma (PDAC), and stomach adenocarcinoma (STAD). DEGs defined as genes with |log2FC|>1 and adj. p-val <0.05 from all GEO datasets examined were cross-referenced to the axon guidance gene signature (GO:0007411) (Supplementary Table S9). DEGs present in at least one dataset are depicted as gray boxes. Genes are arranged in alphabetical order. Genes belonging to the same family are shown in bold and bordered by blue boxes. A heatmap comparing the expression level in all datasets examined is shown in Supplementary Figure S19. Abbreviations: DEG, differentially expressed gene; HNSCC, head and neck squamous cell carcinoma; PDAC, pancreatic ductal adenocarcinoma; STAD, stomach adenocarcinoma; FC, fold change and adj. *p*-value, adjusted *p*-value; GEO, gene expression omnibus.

The finding that neurodevelopmental genes were dysregulated in all the cancers analyzed was of particular note given the role of these pathways in endogenous nerve growth during neural development and nerve plasticity in adults. Further analysis revealed that a number of the most common neurodevelopmental DEGs in PDAC, HNSCC, and STAD were classical axon guidance genes including members of the semaphorin, Eph/ephrin, laminins, Robo, and Wnts. Therefore, we next determined the extent to which axon guidance pathways were dysregulated in PDAC, HNSCC, and STAD (Figure 1C). An axon guidance gene signature was constructed (GO:0007411) and cross-referenced with the DEGs from the PDAC, HNSCC, and STAD datasets (Supplementary Tables S9, S10). The number of dysregulated axon guidance genes was 50 for HNSCC, 58 for PDAC, and 79 for STAD (Figures 1C, 4). Twelve individual axon guidance genes were dysregulated in all three cancers examined, namely, *BOC, CXCL12, EDNRA, EFNA5, EPHA2, FLRT3, PLXNC1, RAC2, SEMA3C, TRIO, UNC5B,* and *WNT5A*. By grouping ligand/receptor genes from the same pathway together, we noted that the individual gene paralog disrupted often differed between different datasets or different cancers. From this, a common theme emerged where several pathways were consistently disrupted by dysregulation of at least one paralog/family member in different datasets and cancers. Comparison of dysregulated axon guidance molecules in datasets from PDAC, HNSCC, and STAD revealed a total of 52 different dysregulated genes belonging to 17 different gene families (Figure 4). This included the ephrin–Eph, semaphorin–neuropilin/plexin, Slit–Robo, laminins, FLRTs, Uncs, and Wnt axis in addition to other signaling molecules. Taken together, these findings support the notion that axon guidance programs were dysregulated in PDAC, HNSCC, and STAD. Furthermore, the data suggested that the different cancers analyzed may have evolved differently to disrupt the same molecular pathways by dysregulation of different paralog genes from the same pathway.

### 2.5 Analysis of TCGA datasets revealed axon guidance pathways to be dysregulated in a broad range of cancers

**FIGURE 5:**
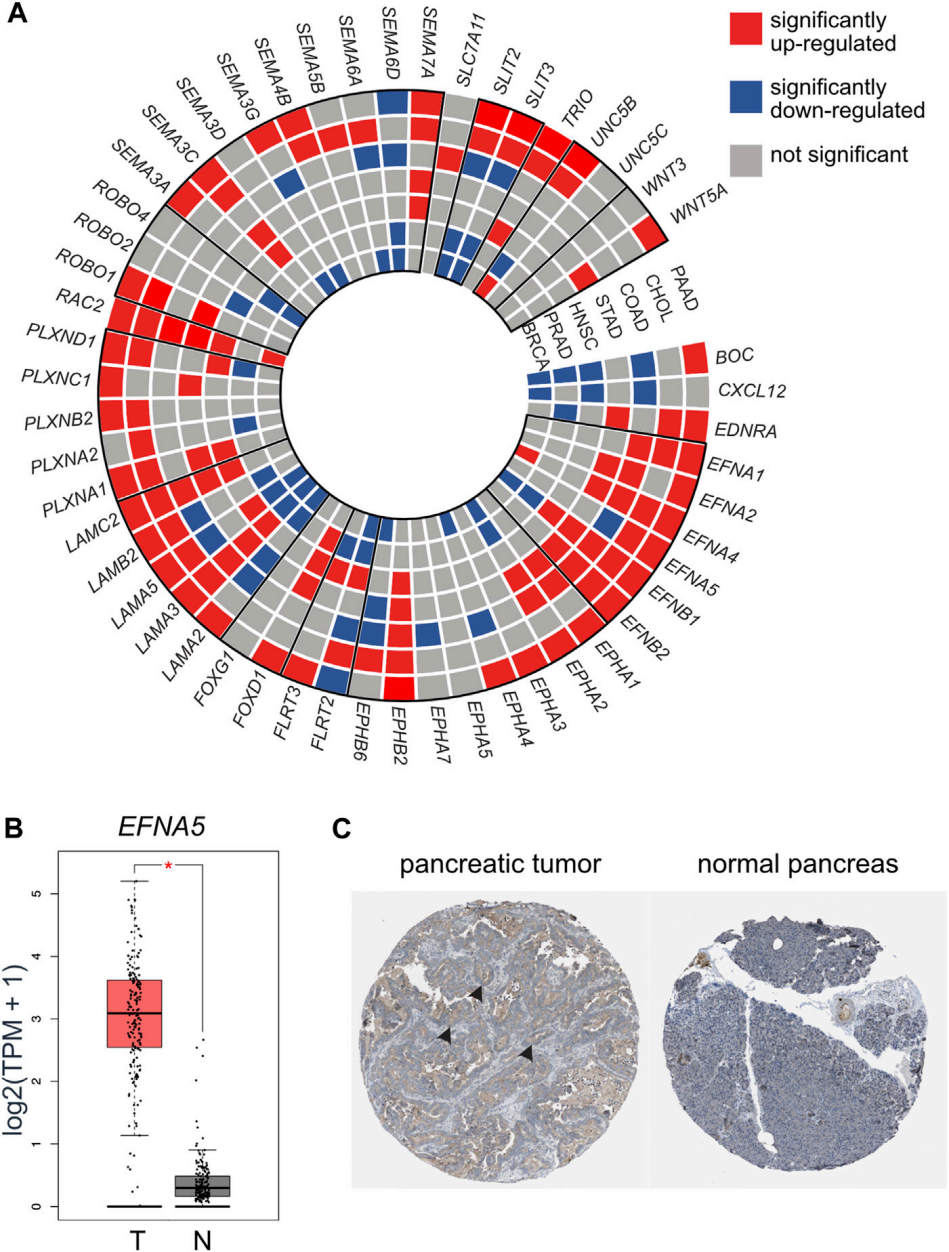
TCGA dataset analysis revealed axon guidance genes were dysregulated in a broad range of cancers associated with PNI. **(A)** A wheel chart depicting dysregulated axon guidance genes in the TCGA datasets. Cancer types are depicted as concentric circle layers and individual genes depicted as slices. Paralog genes are grouped with a bold outline. Significantly upregulated (red), downregulated (blue), and genes which did not reach statistical significance (gray) between tumor and control tissues are depicted. **(B)** mRNA expression of selected gene *EFNA5* in PAAD (Tumor (T) = 179 samples) and normal pancreas from TCGA and GTEx cohorts (normal (N) = 171 samples). **(C)** Immunohistochemistry of an example differentially expressed molecule EFNA5 in pancreatic tumor (https://www.proteinatlas.org/ENSG00000184349-EFNA5/pathology/pancreatic+cancer#32836_B_4_2) and normal pancreas (https://www.proteinatlas.org/ENSG00000184349-EFNA5/tissue/pancreas#32837_A_3_3) image credit: Human Protein Atlas version 22 proteinatlas.org (Uhlén et al., 2015; Uhlen et al., 2017), arrow heads depict overexpression of EFNA5 (brown staining) in the tumor sample. Abbreviations: PNI, perineural invasion; BRCA, breast cancer; PRCA, prostate cancer; HNSCC, head and neck squamous cell carcinoma; STAD, stomach cancer; COAD, colon adenocarcinoma; CHOL, cholangiocarcinoma; PAAD, pancreatic adenocarcinoma; TPM, transcripts per million; TCGA, The Cancer Genome.

We next aimed to examine if axon guidance pathways and molecules were disrupted more broadly in cancers with a high incidence of PNI and to examine the validity of the findings from the GEO data cohorts. For this analysis, independent datasets from TCGA cohorts were analyzed. TCGA cohorts are composed of RNA-Seq data compared with the GEO datasets which are composed of microarray data. In addition to HNSCC, PDAC, and STAD, other cancer types commonly associated with PNI such as prostate (PRCA), breast (BRCA), cholangiocarcinoma (CHOL), and colon (COAD) were also included (Figure 1D; Figure 5A, Supplementary Figure S18; Supplementary Table S11). Consistent with the analysis from the GEO datasets, the analysis of TCGA datasets revealed that several members of axon guidance families were dysregulated among the cancer types analyzed (Figure 5; Supplementary Table S11). Overall, several major axon guidance pathways were consistently dysregulated in both the GEO and TCGA dataset analysis including Eph/ephrin, laminins, slit/robo, and semaphorins/plexins. Semaphorins were found more frequently dysregulated in HNSCC and STAD from GEO cohorts compared with TCGA cohorts. Of note, from this TCGA analysis, axon guidance genes were found most frequently upregulated for PDAC and CHOL cancers (upregulated genes in red in Figure 5A). For example, the axon guidance ligand *EFNA5* was robustly and significantly upregulated in PDAC samples at the RNA level and was also noted at the protein level (Figures 5B, C). This was in sharp contrast to BRCA and PRCA, where axon guidance genes were less frequently dysregulated and had a trend toward downregulation (downregulated genes in blue in Figure 5A).

Taken together, this analysis using TCGA cohorts supported and extended the findings using GEO cohorts that axon guidance programs were broadly dysregulated in several cancers with a high incidence of PNI. Furthermore, it confirmed the result of our previous analysis that more commonly gene families rather than individual genes show distinct trends.

### 2.6 Dysregulation of axon guidance pathways linked with overall survival

**FIGURE 6:**
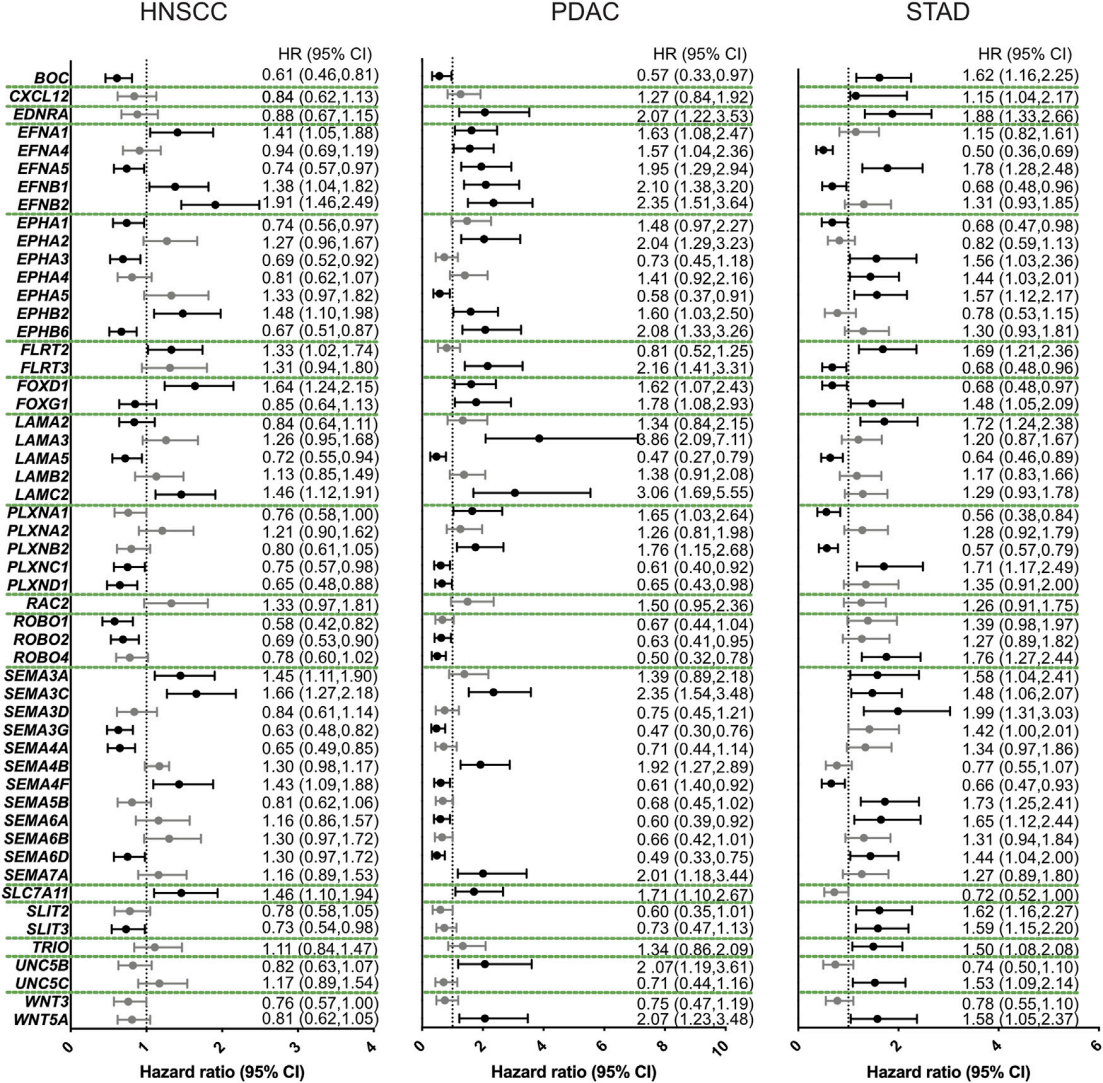
Axon guidance gene expression influences disease survival. Forest plots of the overall disease survival with respect to relative gene expression. For this analysis, the reference group included patients with low expression of the candidate gene. The dotted line represents a hazard ratio (HR) of 1. Higher expression of candidate genes was associated with significant less overall survival when HR > 1, with a confidence interval (CI) of 95%. These are the data points to the right the dotted line. Higher expression of candidate genes was associated with more overall survival when HR < 1 and 95% CI. These are the data points to the left of the dotted line. Abbreviations: HNSCC, head and neck squamous cell carcinoma; PDAC, pancreatic ductal adenocarcinoma; STAD, stomach adenocarcinoma; HR, hazard ratio; CI, confidence interval.

**FIGURE 7:**
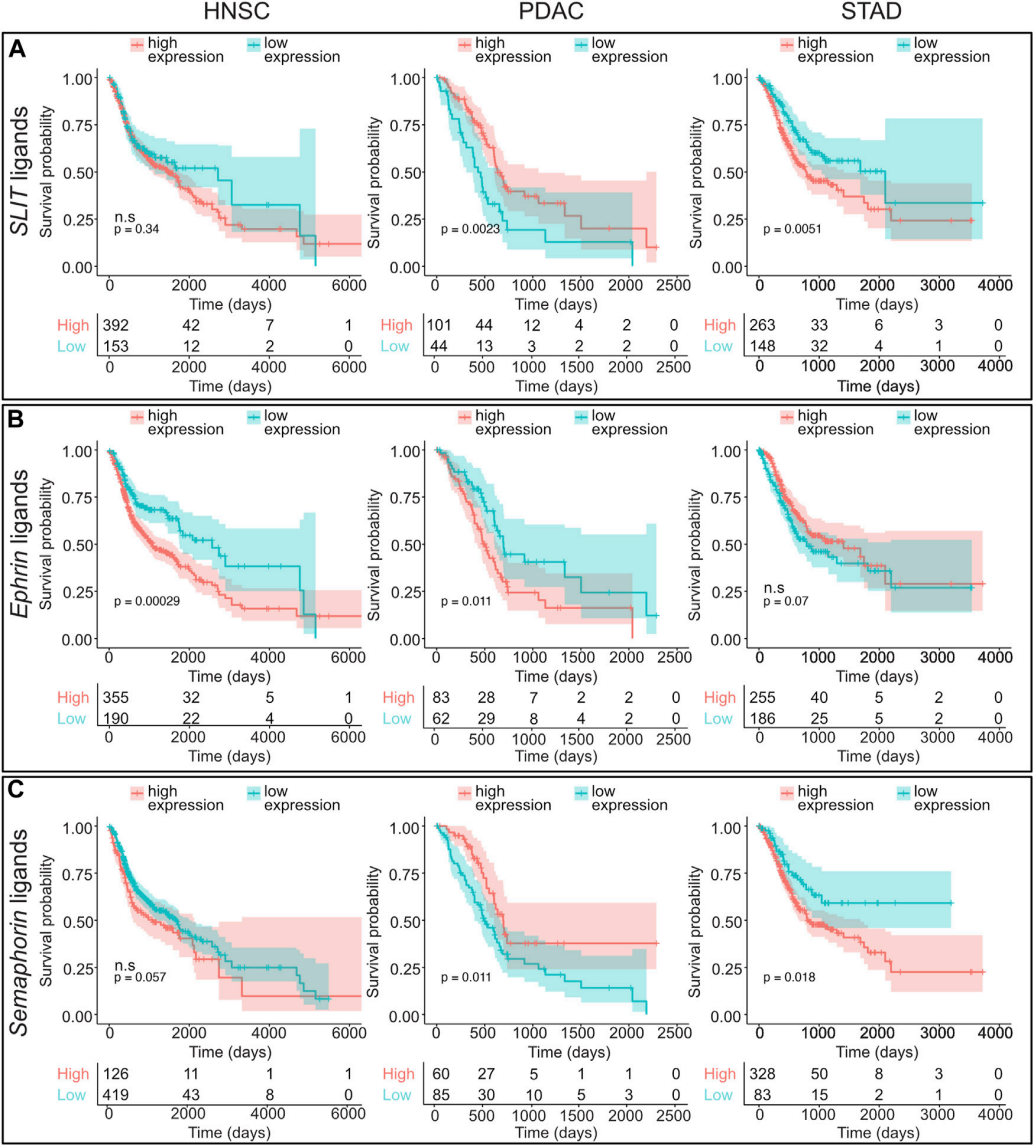
Survival analysis of axon guidance gene families analyzing cohorts of paralog genes. Kaplan–Meier plots and number of patients at risk are shown which visualize HNSCC, PDAC, and STAD overall survival based on combined expression levels for the following paralog gene families: **(A)** SLIT ligands (*SLIT1*, *SLIT2*, and *SLIT3*), **(B)** EPHRIN ligands (*EFNA1*, *EFNA2*, *EFNA3*, *EFNA4*, *EFNA5*, *EFNB1*, *EFNB2*, and *EFNB3*), and **(C)** semaphorin ligands (*SEMA3A*, *SEMA3B*, *SEMA3C*, *SEMA3D*, *SEMA3E*, *SEMA3F*, *SEMA3G*, *SEMA4A*, *SEMA4B*, *SEMA4C*, *SEMA4D*, *SEMA4F*, *SEMA4G*, *SEMA5A*, *SEMA5B*, *SEMA6A*, *SEMA6B*, *SEMA6C*, *SEMA6D*, and *SEMA7A*). For this analysis, patients were segregated in two cohorts (low and high ligand expression) based on the most significant cut-off value for the combined genes. Low- and high-expression groups are depicted in blue and red curves, respectively. Abbreviations: HNSCC, head and neck squamous cell carcinoma; PDAC, pancreatic ductal adenocarcinoma; STAD, stomach adenocarcinoma; ns, not significant.

Since axon guidance families were dysregulated in a number of cancers associated with PNI, we next asked if the expression level of axon guidance molecules and pathways was correlated with disease survival. For this analysis, Kaplan–Meier plots were constructed using best cutoff expression values for dysregulated axon guidance genes within the HNSCC, PDAC, and STAD cohorts (Supplementary Table 12; Figure 6). For pathways where several paralogs were dysregulated, a combined Kaplan–Meier plot for all paralogs was constructed (Figure 7 and Supplementary Figure S20). These data revealed several trends.

Growth factors, including WNT, FGF, and SHH pathways, have been shown to act as axon guidance molecules (Bovolenta, 2005; Sánchez-Camacho and Bovolenta, 2009; Yam and Charron, 2013). Expression above the cutoff of *WNT3* showed a marginal trend toward overall better survival; however, this was not significant. In contrast, expression above the cutoff of the non-canonical WNT gene, *WNT5A* gene was associated with a significant overall worse survival for PDAC and STAD. Expression of a receptor of the growth factor SHH, *BOC* showed a significant impact on survival for all cancers examined with expression above the cutoff in HNSCC and PDAC associated with better survival and worse survival in STAD (Figure 6; Supplementary Table 12).

We observed that, among others, three classical axon guidance pathways semaphorin/plexin/neuropilin, slit/robo, and Eph/ephrin axis influenced overall survival in STAD (Figures 6, 7; Supplementary Figure S20; Supplementary Table 12). *SLIT* and *ROBO* genes conferred similar prognosis trends to each other within the cancers examined. In particular, the expression above the cutoff of *SLIT* and *ROBO* family members was associated with better prognosis in HNSCC and PDAC, and with worse prognosis in STAD (Figures 6, 7A, Supplementary Figure S20; Supplementary Table 12).

We observed that the expression above the cutoff for *ephrin* ligand genes showed a general trend toward worse overall survival for HNSCC and PDAC (Figures 6B, 7B). Strikingly, for PDAC, all *ephrin* genes examined showed a statistically significant worse survival when expressed above the cutoff levels, whereas for HNSCC and STAD, this was restricted to a few members of the family, *EFNA1*, *EFNB1*, and *EFNB2* for HNSCC and *EFNA5* for STAD (Figures 6, 7B; Supplementary Table 12). In contrast, the expression levels of the *ephrin* receptors produced differing effects on survival depending on the particular gene and cancer type.

For *SEMA3A*, *SEMA3C,* and *SEMA7A* as well as the semaphorin receptor *neuropilin,* there was a clear trend where the expression above the cutoff was associated with worse survival for all three cancers (Figures 6, 7C; Supplementary Table 12). Of these, only S*EMA3C* was significant for all three cancers. In contrast, the expressions of other *semaphorin* ligand family members and *plexin* receptors were associated with different survival trends depending on the individual gene cancer analyzed (Figure 6, Supplementary Figure S20; Supplementary Table 12).

Taken together, this analysis revealed that the dysregulated axon guidance pathways significantly impact on survival dynamics in HNSCC, PDAC, and STAD.

## 3 Discussion

The most important finding from this study was the observation that the tumor and tumor microenvironment are likely important sources of signals for nerve–tumor interactions including neural tropism, nerve plasticity, and PNI in a wide range of solid tumors throughout the body. Among other things, for the cancers analyzed, a common feature was disruption of the extracellular matrix and core neurodevelopmental pathways. Furthermore, the data supported the notion that the cancers analyzed may have evolved distinctly at a molecular genetic level to achieve disruption of common pathways by dysregulation of alternative paralog genes. These results together with the finding that neurodevelopmental pathways were associated with overall disease survival highlighted the pathways’ importance during cancer progression, their potential in influencing nerve/tumor interactions and in providing disease biomarkers.

### 3.1 Dysregulation of the extracellular matrix was a common feature of the cancers analyzed

Analysis of mRNA expression of whole tumor samples revealed that the extracellular matrix was the most dominantly disrupted biological process and cellular component in all cancers examined. This analysis supported the notion that the formation and structure of the extracellular matrix was a key component in HNSCC, PDAC, and STAD, consistent with the known desmoplasia during cancer progression. Extracellular matrix (ECM) not only functions as a scaffold for the tissues, which is remodeled during cancer pathogenesis, but also provides a reservoir for molecules that signal to different tumor-associated cell types (Clause and Barker, 2013). This may be important in the growth of nerves in tumors by anchoring and/modulating the activity of molecules which facilitate nerve–tumor interactions. Supporting the role of the ECM in nerve–tumor interactions, ECM molecules have been shown to be dysregulated in a transcriptomic analysis of HNSCC comparing samples with clinical PNI to control and non-PNI tumors (Eviston et al., 2021). Several studies have associated ECM molecules with PNI (Mukherjee et al., 2022). For example, laminin 5 was reported to be overexpressed in HNSCC specimens and significantly correlated with the presence of PNI (Anderson et al., 2001). Fitting with this idea, it is well-established that ECM molecules support neural growth and guidance during embryonic development both by acting as a support substrate for growing axons but also by sequestering ligands and co-binding to classical axon guidance receptors and ligands (Liesi, 1990; Pires-Neto et al., 1999; Shipp and Hsieh-Wilson, 2007).

### 3.2 Neurodevelopmental programs are associated with nerve growth and perineural invasion

Our results showed that dysregulation of neurodevelopmental pathways was a key feature associated with cancers with PNI. Previous work has identified a number of molecules involved in cancer nerve growth and PNI in different cancer types (Liebig et al., 2009; Bapat et al., 2011; Amit et al., 2016; Liang et al., 2016; Liu et al., 2022). Our analysis of the PNI gene lists from gastric cancer (Jia) and HNSCC (Eviston) revealed a high proportion of dysregulated neurodevelopment genes associated with PNI (Jia et al., 2019; Eviston et al., 2021). Within the Jia gastric cancer PNI gene list, 14% were known axon guidance genes including classical axon guidance pathways, neurotrophins, and chemokines (Jia et al., 2019). This is of particular note since axon guidance genes drive the growth and migration of developing axons in the embryo and synaptic and neural plasticity of nerves (Cho and Miller, 2002; Butler and Tear, 2007; Li and Ransohoff, 2008; Kolodkin and Tessier-Lavigne, 2011; Stoeckli, 2018). The study here suggests that axonal guidance molecules could have a broad role in nerve growth and PNI during tumor development and progression, which is supported by experimental research in different cancer types (Biankin et al., 2012; Shao et al., 2013; Göhrig et al., 2014; Schmitd et al., 2018; Furuhashi et al., 2021; Yin et al., 2021).

Previous studies have reported dysregulation of semaphorins and their cognate receptors neuropilins and plexins in a wide range of cancers. Particularly, class 3 semaphorins have shown significant amplification in the genomes of pancreatic cancer patients (Biankin et al., 2012). The analysis presented in the study here identified *SEMA3C* as significantly upregulated in pancreatic, bile duct, stomach, and head and neck squamous cell carcinoma, which was correlated with worse overall survival. Consistent with this, in prostate cancer, the SEMA3C/PlexinA2/NRP1 axis has been shown to be correlated with PNI and nerve density within the tumor cancer model (Yin et al., 2021).

Furthermore, we noted that members of the EFN/EPH pathway were the most frequently upregulated in the majority of the selected cancers. Several studies have suggested that these molecules may have a role in nerve/tumor interactions. For instance, *EPHA4A* was found upregulated in a PDAC cell line (MIAPACA), which enhanced their migration and influenced neurite outgrowth in a DRG-tumor cell *in vitro* assay (Furuhashi et al., 2021). In another study, *EFNA1/EPHA2* was correlated with TNM stage and perineural invasion in adenoid cystic carcinoma of the salivary glands (Shao et al., 2013).

For the Slit/Robo pathways, differing results have been obtained. Genomic aberrations of axon guidance SLIT/ROBO pathways were commonly found in an early-stage pancreatic cancer cohort (Biankin et al., 2012). Consistent with this, ectopic expression of SLIT2 in PDAC cell lines impaired cell migration, invasion, and interaction with neuronal cells in an *in vitro* co-culture assay, suggesting that Slit expression inhibits tumor progression (Göhrig et al., 2014). However, other evidence suggests that SLIT/ROBO expression increases progressively from normal pancreas to acinar-ductal metaplasia and PDAC (Biankin et al., 2012). Consistent with this, our data showed that in PDAC, *SLIT* expression was high and high expression of either Slits or their cognate ligands Robos was linked to poor survival. In contrast, in gastric, prostate, and breast cancers, Slits appeared to be downregulated in tumor tissue, and lower Slit family expression was associated with worse survival in gastric cancer. These contrasting results might reflect a difference in the expression levels across the disease stages and/or the type of neurons or other cells that are present in each cancer type.

Taken together, while individual studies have revealed several, sometimes contradictory findings, a clear picture has emerged that these classical and other neurodevelopmental molecules have a major role in nerve–tumor interactions.

### 3.3 Evolution of cancer between individuals and cancer types—dysregulation of different paralog genes from the same pathways

Substantial evidence supports the view that nerve–tumor interactions have a major pathophysiological impact in cancer development and progression (Amit et al., 2016; Liang et al., 2016; Zahalka and Frenette, 2020). Therefore, targeting cancer nerves has gained attraction as a potential therapy. However, at the time of writing, no treatment directly targeting this process is in place. Interestingly, this study revealed that a common feature between different cancers analyzed was the disruption of the same molecular neurodevelopmental pathways, albeit via disruption of different paralog genes. This observation suggested that the cancer types analyzed might have evolved independently, with the overall effect of disrupting the same core pathways. This concept also applied to different individuals with the same cancer type. This notion was supported by the observation that by comparing several data cohorts from the same cancer type, individual genes were significantly dysregulated in some datasets, but not in others. Similarly, even when highly significant, the degree of dysregulation between samples from different individuals for a particular gene varied considerably (e.g., see Supplementary Figure S18).

This finding has important implications for future cancer drug design. In order to target signaling pathways in a systematic way, an important consideration can be to account for genetic variation between individuals. For example, one of the most promising pathways to date for targeting cancer nerve growth is the NGF signaling pathway, a neurotrophic factor, known to have a neurodevelopmental role. NGF has been shown in preclinical studies to mediate tumor nerve growth and has subsequently been targeted in clinical trials in prostate cancer via a small-molecule inhibitor of the NGF cognate receptor TRKA (Collins et al., 2007; Zahalka and Frenette, 2020). While this intervention reduced metastasis in patients where the TRKA receptor was disrupted in the tumor itself, no effect has been reported on nerve growth (Collins et al., 2007; Smith et al., 2016; Drilon et al., 2018). Importantly, for individuals where the TRKA receptor was not disrupted in the tumor, no positive effect was observed (Collins et al., 2007; Smith et al., 2016; Drilon et al., 2018). This highlights the importance of a conclusion of this study that given the genetic heterogeneity/evolution between individuals, targeting individual genes is likely to result in variable outcomes and personalized approaches or targeting pathways more broadly may be needed.

### 3.4 The tumor and tumor environment as a source of signals for nerve-tumor interactions

The cancer datasets analyzed here are based on analysis of bulk tumors and control samples which are cellularly heterogeneous. Therefore, in this and other studies using similar cohorts, analysis using standard bioinformatics tools is likely to embody changes among the most abundant cells and transcripts. This likely represents a broad range of processes dysregulated during cancer development and progression. When investigating signaling between nerves and tumors, an important consideration is that tumor cells and cells from the microenvironment, such as fibroblasts, are commonly highly represented within the samples compared with, for example, tumor nerves (Hanahan and Coussens, 2012). This is especially relevant in this context since in many cases, the neural cell bodies of the nerves which innervate organs in the body are located at a distance from the tumor, in ganglia and/or CNS. In these cases, the neural cell bodies are unlikely to be included in the sample. Together, this means that anatomically neurons and other cells in the nerves represent a relatively small cytoplasmic volume of the tumor. Therefore, the biological pathways most relevant to tumor-induced nerve–tumor interactions including PNI during tumor pathogenesis may not be ranked highly using a bioinformatics standard approach. This will be confounded by the limitations of the datasets which comprise disease-free adjacent tissue as control. In the datasets analyzed, the transcriptome of datasets using adjacent tissue controls segregated poorly from tumor samples. The implication of using tumor samples of this type is that our results are more likely to identify the signaling molecules (paracrine and juxtracrine ligands) coming from the tumor which attract nerves rather than the protein produced by neurons or glial cells that signal to the tumor cells. We identified several ligand molecules that are both dysregulated and are correlated with survival including members of the SLIT, ephrin, and semaphorin families, growth factors, chemokines, and other ligand classes. Interestingly, we observed the dysregulation of a number of cognate receptors of these ligands, which suggests that these pathways are likely to play a role in other processes in tumor development and progression. Such pleotropic roles of axon guidance molecules, for example, immune response and vasculogenesis, have been well-documented (Neufeld et al., 2002; Fujiwara et al., 2006; Zhang and Hughes, 2006; Ji and Ivashkiv, 2009; Dai et al., 2019; Kiseleva and Rutto, 2022).

Single-cell RNA sequencing would provide a higher resolution of which molecule may be dysregulated in different cells. However, in determining signals from the growing neuron, a major caveat to that approach is the relatively low number of transcripts at the growth cone and branching nerve. Furthermore, in order to identify potential target pathways for nerve growth and PNI, in this study, datasets where patient samples have been segregated into PNI/no PNI have been analyzed. The caveat to this is that since the presence of PNI is associated with more advanced disease, it is likely to reflect a number of different processes associated with a more advanced disease stage.

Finally, the neurodevelopmental and axon guidance gene signatures used in this study were calculated using the QuickGo repository. Through this, we noted that molecules and pathways known to play a role in neurodevelopment and axon guidance were absent from these computed gene signatures. For example, connexins which form gap junction channels, which are known to play a role in the guidance of neurons in some contexts, were absent from the axon guidance gene signature in QuickGo. Therefore, further analysis may reveal other axon guidance pathways important in tumor development and progression.

### 3.5 Dysregulation of axon guidance pathways showed a different trend among breast and prostate cancers compared to the other cancers analyzed

Finally, we observed that the number of dysregulated axon guidance genes and if they were up or downregulated varied between different cancers analyzed. For example, PDAC showed a relatively high number of dysregulated axon guidance genes with a tendency toward upregulation. In contrast, the sex-enriched/-specific cancers breast and prostate cancers showed a tendency to fewer dysregulated axon guidance genes with a trend toward downregulation. Of note in this respect, PDAC is commonly diagnosed at a relatively late disease stage, whereas breast and prostate cancers are often detected at earlier disease stages. The difference in axon guidance gene expression between PDAC and breast/prostate cancers could therefore reflect the disease stages of samples analyzed. An alternative explanation is that different organs are innervated by different nerve types which may respond differently to the same axon guidance cues. It is well established that during the normal growth and guidance of neurons during development, different neurons use different combinations of axon guidance molecules to navigate to their precise target (Tessier-Lavigne and Goodman, 1996; Chen et al., 2008; Kolodkin and Tessier-Lavigne, 2011; Blockus and Chédotal, 2016). Axon guidance cues are commonly attractants to some groups of neurons and repellants to others (Kolodkin and Tessier-Lavigne, 2011; Blockus and Chédotal, 2016; Boyer and Gupton, 2018). Thus, it is highly likely that in the adult, the neurons within different organs would respond differently to the same cues. In this way, in order for the tumor to evolve the same biological outcome, ectopic nerve growth would require dysregulation of the axon guidance pathways in a different way.

### 3.6 Concluding remarks

Taken together, this study showed the tumor and tumor microenvironment as a potential reservoir of signals for nerve–tumor interactions in a wide range of solid tumors throughout the body. Moreover, it supports the idea that dysregulation axon guidance and other neurodevelopmental molecular pathways is a core feature in cancers with a high incidence of tumor nerve growth and PNI. This highlights the potential broad role of neurodevelopmental pathways in nerve plasticity including neural signaling, nerve growth, and PNI during cancer development and progression as a general concept for a wide range of solid tumors throughout the body.

## 4 Materials and methods

### 4.1 Cancer nomenclature

Several data sources and different cancer types were analyzed in this study. For several datasets used, the cancers are referred to in different ways. For clarity, the nomenclature used in this paper has been made uniform. The cancers where more than one name are noted in the datasets are as follows, and the nomenclature referred to by the original datasets is referred to in Table 1: pancreatic ductal adenocarcinoma (PDAC) is used in this paper for datasets referred to as PDAC, pancreatic adenocarcinoma (PAAD) and pancreatic cancer; head and neck squamous cell carcinoma (HNSCC) is used in this study for datasets referred to as HNSCC, head and neck cancer and oral squamous cell carcinoma; stomach adenocarcinoma (STAD) used in this paper for datasets which are referred to as STAD and gastric cancer.

### 4.2 Gene Expression Omnibus (GEO) datasets

To identify molecular candidates in different cancers with a high incidence of PNI, a systematic search for datasets from the public Gene Expression Omnibus (GEO) database was performed (https://www.ncbi.nlm.nih.gov/geo/) (Edgar et al., 2002; Barrett et al., 2012; Clough and Barrett, 2016). The search criteria included the terms pancreatic cancer, pancreatic adenocarcinoma, gastric cancer, stomach cancer, cholangiocarcinoma, head and neck squamous cellular carcinoma, head and neck cancer, colorectal cancer, colon and rectal cancer, prostate cancer, and breast cancer. The GEO datasets used in this study were composed of microarray data from tumor biopsies or resected tumors and non-cancerous tissue from the biopsies or paired healthy tissue. Datasets were selected according to the following criteria: 1) expression profile by array; 2) type of sample: tissue; 3) organism: *Homo sapiens*; and 4) sample size of at least 6. The quality of the datasets was analyzed using several methods including analysis of the distribution of the expression values, unsupervised clustering, and for datasets which had an unbalanced group composition, a group composition equalization method was used (Supplementary Figures S1–S13). Volcano plots were constructed to assess the data distribution, statistical significance (−log10 *p*-value), and fold change (log2 fold change) (Figure 2 and Supplementary Figure S14). Datasets whose volcano plots showed no clear significant fold changes were excluded.

### 4.3 Unsupervised clustering

Unsupervised clustering analysis was performed using the R programming language. The datasets were obtained from the Gene Expression Omnibus (GEO) repository using series matrix files. The data were preprocessed when needed for normalization employing the quantile normalization method. The optimal number of clusters was determined using a “within groups sum of squares” (WSS) plot. The k-means clustering algorithm was then used and the resulting clusters visualized using a cluster plot.

### 4.4 Dataset equalization analysis

A dataset equalization analysis was performed on GEO datasets which had an unbalanced sample composition. The script used was based on the code in the limma package from GEO2R and is shown in the Supplementary infomation. In short, DEGs were generated for each dataset by comparing an equal number of control and tumor samples, where the samples from the larger group (tumors or controls) were randomly selected from the dataset. This was repeated 100 times, and the list of DEGs and frequency they occur was calculated. This was performed first using all probes from the screen and then using only the probes identified using the limma package in GEO2R as occurring in all or at least three datasets from the cancer being analyzed (Supplementary Figures S1–S5).

### 4.5 Data processing and differentially expressed gene identification

All datasets were composed of log-transformed expression data. The microarray datasets were processed using the limma R package in the GEO2R platform (https://www.ncbi.nlm.nih.gov/geo/geo2r/) (Ritchie et al., 2015; Clough and Barrett, 2016). Fold-change (FC) and adjusted *p*-values were calculated through the comparison of tumor versus control tissues using the statistical test Benjamini and Hochberg with false discovery rate. When multiple probes were available for the same gene, all probes were used for the analysis. Differentially expressed genes (DEGs) were defined as genes with |log2(FC)|≥1 and adjusted *p*-values <0.05. Volcano plots of DEGs were constructed for each dataset using the ggplot2 R package (Figure 2 and Supplementary Figure S14) (Wickham, 2011). Subsequently, the DEGs from the same type of cancer were intersected using the tool VENN DIAGRAMS from Van de Peer Lab http://bioinformatics.psb.ugent.be/beg/tools/venn-diagrams. An intersection of the DEGs identified from different cancers was performed using DEGs common in at least three different datasets from the same cancer type. This intersection analysis is presented in a diagram that was constructed using the bioinformatics software DiVenn (Sun et al., 2019).

### 4.6 Functional enrichment analysis of differentially expressed genes

The enrichment analysis was performed to gain insights about the functional meaning of differentially expressed genes. This was performed using the R package ClusterProfiler. The function enrichGo was used to obtain the cellular component ontology (CC), biological process (BP), and molecular function (MF). A significance threshold of *p*-value <0.05 and q-value <0.05 for enriched terms was used. The enriched terms were visualized using dotplot R function, with the top 10 enriched terms depicted (Figures 2I—K and Supplementary Figure S15).

### 4.7 Neurodevelopment and axon guidance gene signature analysis

A gene signature comprising 2,193 genes annotated for human nervous system development (GO:0007399) was constructed using the platform QuickGO (Binns et al., 2009) (Supplementary Table S4, Supplementary Figure S16). This neurodevelopmental gene signature consisted of terms which have been associated with the process that leads to the formation and maturation of the nervous tissue over time. Subsequently, DEGs from the same type of cancer were combined and compared with the neurodevelopmental signature. An intersection analysis between the cancers analyzed was performed by intersecting all neurodevelopmental DEGs for STAD, PDAC, and HNSCC using DiVenn (Sun et al., 2019). A hierarchical clustering heatmap was generated to visualize the neurodevelopmental DEGs common to all cancers using the package complex heatmap of R software (Figure 3).

Similarly, a gene signature consisting in 281 genes annotated for axon guidance (GO:0007411) was constructed using QuickGO (Binns et al., 2009) (Supplementary Table S9). This gene signature was cross-referenced with the DEGs identified from PDAC, HNSCC, and STAD GEO datasets to examine the dysregulation of axon guidance pathways (Supplementary Table 10). DEGs are depicted and ordered alphabetically. A heatmap without clustering was constructed to visualize the axon guidance DEGs (Supplementary Table 19). Gene families which were shown to be dysregulated by any of the paralogs across all cancers were selected for further analysis using TCGA data cohorts.

### 4.8 TCGA data and survival analysis

Axon guidance genes belonging to commonly dysregulated gene families in all cancer types were analyzed in the database Gene Expression Profiling Interactive Analysis (GEPIA) (Tang et al., 2017). GEPIA is an interactive web server for analyzing RNA-seq data from the TCGA and the GTEx projects. The expression values of DEGs in several PNI-associated cancers including breast adenocarcinoma (BRCA), cholangiocarcinoma (CHOL), colon adenocarcinoma (COAD), head and neck squamous cellular carcinoma (HNSCC), pancreatic adenocarcinoma (PAAD—referred to here as PDAC), prostate adenocarcinoma (PRAD), and stomach adenocarcinoma (STAD) were compared. The differential thresholds used in this analysis, *p*-value <0.05 and |log2FC|>1, were consistent with the differential thresholds used for the GEO datasets. The TCGA analysis was based on TCGA tumors compared with TCGA normal and GTEx normal tissue. The statistical test used for the comparisons was one-way ANOVA. The expression analysis was summarized in a wheel chart (Figure 5). The expression of each individual axon guidance gene is shown in Supplementary Figure S18.

In order to determine if dysregulation of the genes of interest was associated with overall survival, the survival analysis tool KMplot was used (Lánczky and Győrffy, 2021). Briefly, the hazard ratio (HR) was calculated for each axon guidance gene when comparing tumors with high versus low mRNA expression of the gene being analyzed. For this, the best cut-off values were used for comparison. The summary of the survival analysis is presented in a forest plot constructed with GraphPad software (Figure 6). Genes with HR < 1 and *p* < 0.05 confer worse prognosis when expressed at a relatively low level, whereas genes with HR > 1 and *p* < 0.05 confer worse prognosis when expressed at a relatively high level.

Several paralog genes belonging to the same family were dysregulated in different cancers and different datasets. Therefore, the impact of dysregulation of gene cohorts on survival was examined by combining paralog genes from the same pathway. To do this, TCGA gene expression and survival matrixes were downloaded from the Xena repository (https://xenabrowser.net), and R studio was used to compute the best cut-off value for the paralog gene cohorts and the data displayed with Kaplan–Meier plots. The script is available in the Supplementary information.

## Data Availability

The original contributions presented in the study are included in the article/Supplementary Material; further inquiries can be directed to the corresponding author.

## Abbreviations

Adj. P. val, adjusted *p*-value; BRCA, breast cancer; BP, biological process; CC, cellular component; CHOL, cholangiocarcinoma; COAD, colon adenocarcinoma; DEG, differentially expressed gene; FC, fold change; GC, gastric cancer; GO, gene ontology; GEO, Gene Expression Omnibus; GEPIA, Gene Expression Profiling Interactive Analysis; GTEx, The Genotype-Tissue Expression; HNC, head and neck cancer; HNSCC, head and neck squamous cellular carcinoma; MF, molecular function; PAAD, pancreatic adenocarcinoma; PDAC, pancreatic ductal adenocarcinoma; PNI, perineural invasion; PPI, protein–protein interaction; PRAD, prostate adenocarcinoma; STAD, stomach adenocarcinoma; TCGA, The Cancer Genome Atlas; TNM, Classification of Malignant Tumors; TPM, transcript per million.
