# Supplementary material for: Dysregulation of core neurodevelopmental pathways—a common feature of cancers with perineural invasion
Source: Front Genet. 2023 Aug 31;14:1181775. doi: 10.3389/fgene.2023.1181775 (PMC10501147; doi:10.3389/fgene.2023.1181775)
Supplement: Supplementary file 5 [file DataSheet1.pdf]

Supplementary Material

Dysregulation of core neurodevelopmental pathways – a common feature of cancers with perineural invasion.

Luz María González-Castrillón[1], Maud Wurmser[1], Daniel Öhlund[2] and Sara Wilson[1*]*

*** Correspondence:** Corresponding Author sara.wilson@umu.se

1 Supplementary figures and captions

HNSCC GSE23036: 63 tumors (T); 5 controls (C)

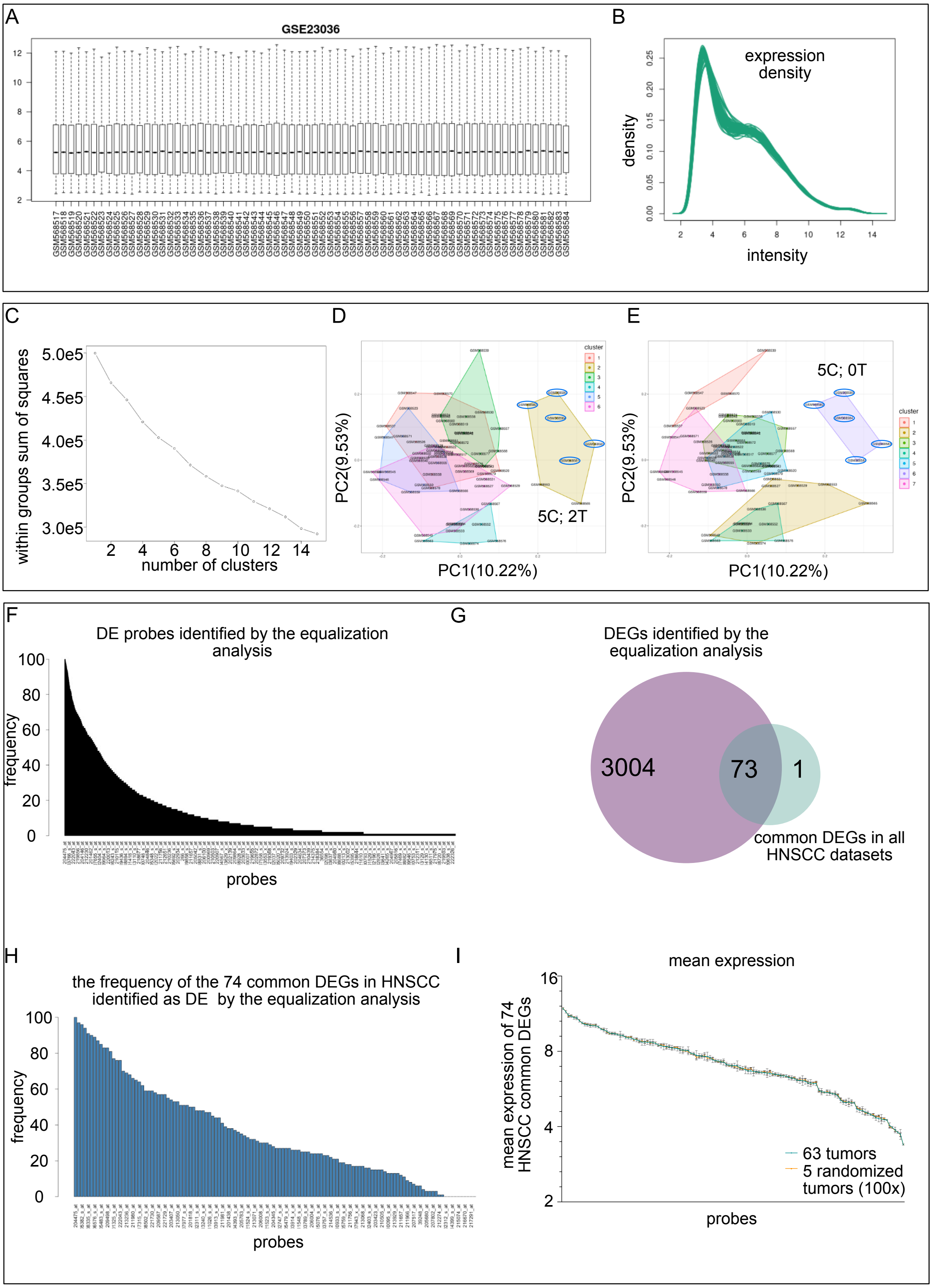

Supplementary Figure 1: Head and Neck Squamous Cell Carcinoma dataset GSE23036: The dataset has 63 tumor (T) and 5 control (C) samples. (**A** - **B**) The distribution of the expression values of the samples in dataset GSE23036 are presented as box plot (**A**) and expression density plots (**B**). (**C** - **E**) Unsupervised cluster analysis. The optimal number of clusters (K) is depicted using a Within groups of sum of squares (WSS) plot (**C**). Cluster plots using 6 (**D**) or 7 (**E**) clusters, were generated using the K-means method. In **D** and **E**, the sample identification numbers are shown and the samples circled in blue are control samples. (**F - I**) Dataset analysis using group equalization. In this analysis differentially expressed genes were identified using the limma package by comparing the control group (5 controls) versus a randomized tumor group (5 tumors) from the dataset with 100 repetitions. The tumor group was selected randomly for each trial. A graph showing the frequency of the differentially expressed probes within the 100-loop trial is shown (**F**). 74 common DEGs were identified from all HNSCC datasets using GEO2R (from **Figure 2D**). A Venn diagram depicting the intersection between the 74 common DEGs identified from all HNSCC compared with DEGs generated using the equalization method in **F** is shown (**G**). A graph showing the frequency of the probes for the 74 common DEGs identified from all HNSCC datasets within the 100-loop trial is shown (**H**). A graph depicting the mean expression values of the 74 common DEGs in all HNSCC datasets in the 63 tumors compared with the mean of the mean expression values in the randomized tumor group using the equalization analysis is shown. The bars represent the standard error of the mean (**I**). Abbreviations: tumor (T), control (C) Within groups of sum of squares (WSS), differentially expressed gene (DEG), differentially expressed (DE), head and neck squamous cell carcinoma (HNSCC), principle component (PC).

HNSCC GSE31056: 23 tumors (T); 73 controls (C)

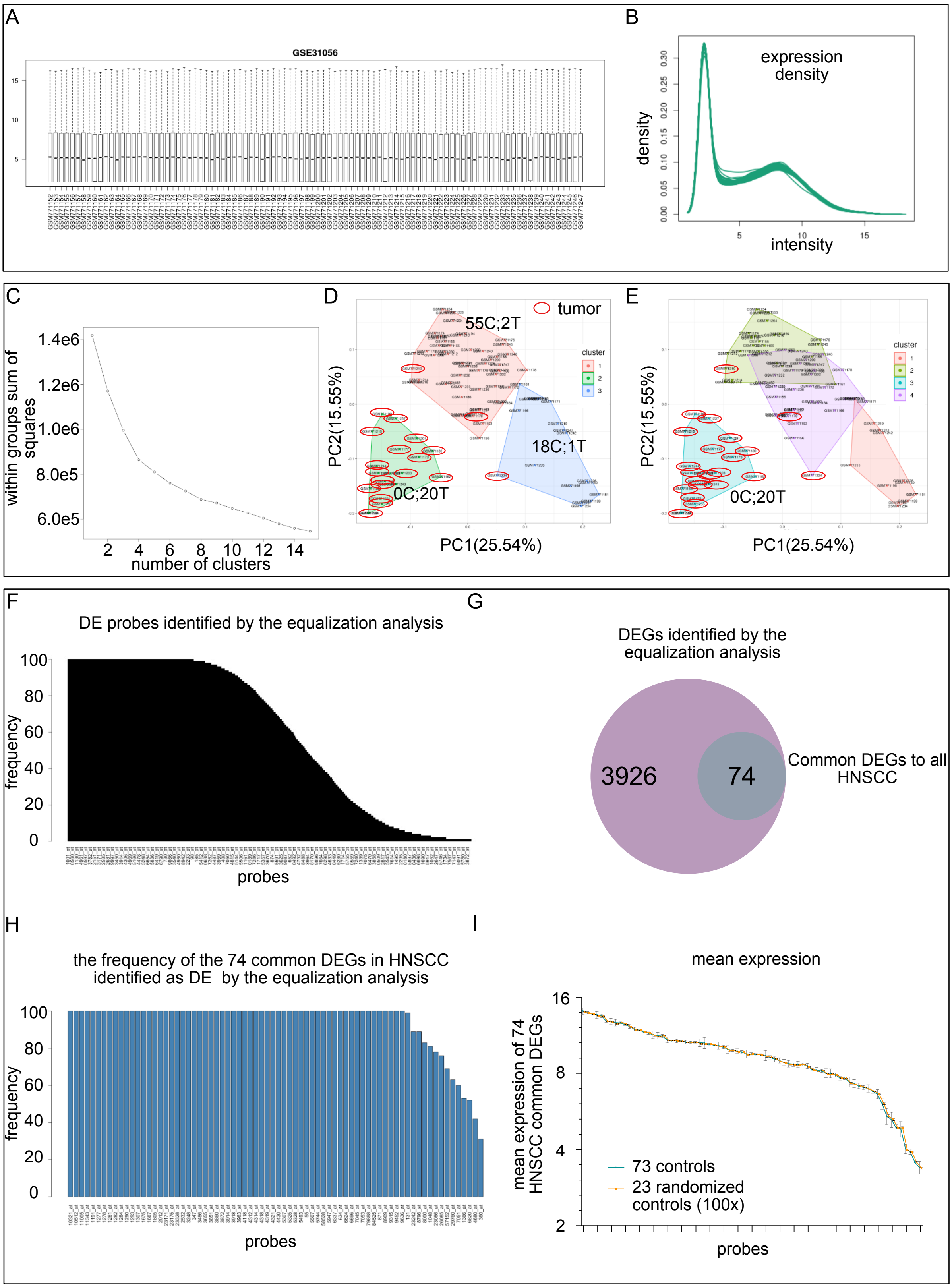

Supplementary Figure 2: Head and neck squamous cell carcinoma dataset GSE31056: The dataset has 23 tumor (T) and 73 control (C) samples. (**A** - **B**) The distribution of the expression values of the samples in dataset GSE31056 are presented as box plot (**A**) and expression density plots (**B**). (**C** - **E**) Unsupervised cluster analysis. The optimal number of clusters (K) is depicted using a Within groups of sum of squares (WSS) plot (**C**). Cluster plots using 3 (**D**) or 4 (**E**) clusters, were generated using the K-means method. In **D** and **E**, the sample identification numbers are shown and the samples circled in red are tumor samples. (**F** - **I**) Dataset analysis using group equalization. In this analysis differentially expressed genes were identified using the limma package by comparing the tumor group (23 tumors) versus a randomized control group (23 controls) from the dataset with 100 repetitions. The control group was selected randomly for each trial. A graph showing the frequency of the differentially expressed probes within the 100-loop trial is shown (**F**). 74 common DEGs were identified from all HNSCC datasets using GEO2R (from **Figure 2D**). A Venn diagram depicting the intersection between the 74 common DEGs identified from all HNSCC compared with DEGs generated using the equalization method in **F** is shown (**G**). A graph showing the frequency of the probes for the 74 common DEGs identified from all HNSCC datasets within the 100-loop trial is shown (**H**). A graph depicting the mean expression values of the 74 common DEGs in all HNSCC datasets in the 73 controls compared with the mean of the mean expression values in the randomized control group using the equalization analysis is shown. The bars represent the standard error of the mean (**I**). Abbreviations: tumor (T), control (C) Within groups of sum of squares (WSS), differentially expressed gene (DEG), differentially expressed (DE), head and neck squamous cell carcinoma (HNSCC), principle component (PC).

PDAC GSE16515: 36 tumors (T); 16 controls (C)

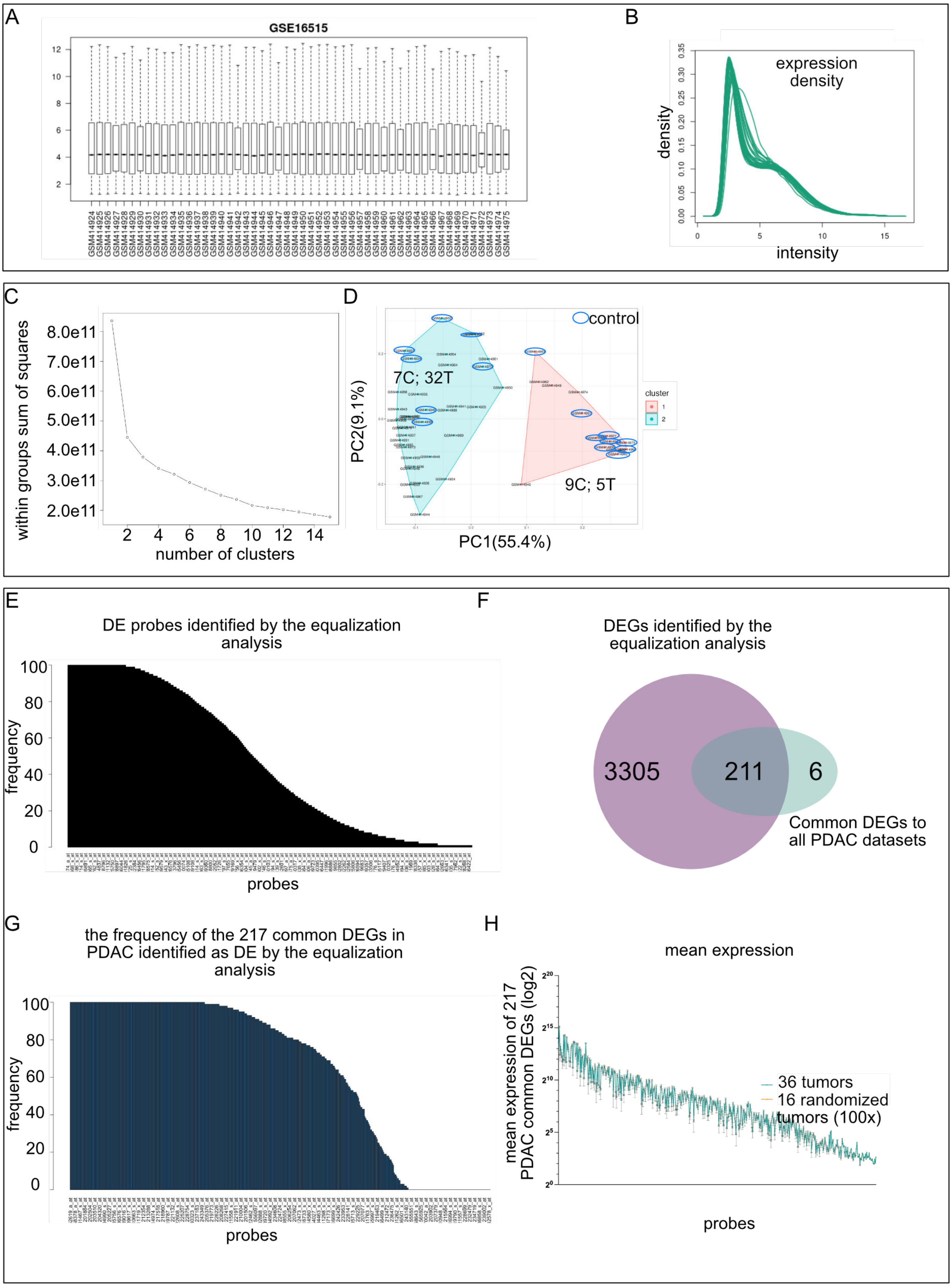

Supplementary Figure 3: Pancreatic ductal adenocarcinoma dataset GSE16515: The dataset has 36 tumor (T) and 16 control (C) samples. (**A** - **B**) The distribution of the expression values of the samples in dataset GSE16515 are presented as box plot (**A**) and expression density plots (**B**). (**C** - **E**) Unsupervised cluster analysis. The optimal number of clusters (K) is depicted using a Within groups of sum of squares (WSS) plot (**C**). A cluster plot using 2 clusters was generated using the K-means method. The sample identification numbers are shown and the samples circled in blue are control samples (**D**). (**E - H**) Dataset analysis using group equalization. In this analysis differentially expressed genes were identified using the limma package by comparing the control group (16 controls) versus a randomized tumor group (16 tumors) from the dataset with 100 repetitions. The tumor group was selected randomly for each trial. A graph showing the frequency of the differentially expressed probes within the 100-loop trial is shown (**E**). 217 common DEGs were identified from all PDAC datasets using GEO2R (from **Figure 2E**). A Venn diagram depicting the intersection between the 217 common DEGs identified from all PDAC compared with DEGs generated using the equalization method in **E** is shown (**F**). A graph showing the frequency of the probes for the 217 common DEGs identified from all PDAC datasets within the 100-loop trial is shown (**G**). A graph depicting the mean expression values of the 217 common DEGs in all PDAC datasets in the 36 tumors compared with the mean of the mean expression values in the randomized tumor group using the equalization analysis is shown. The bars represent the standard error of the mean (**H**). Abbreviations: tumor (T), control (C) Within groups of sum of squares (WSS), differentially expressed gene (DEG), differentially expressed (DE), pancreatic ductal adenocarcinoma (PDAC), principle component (PC).

STAD GSE33651: 40 tumors (T); 12 controls (C)

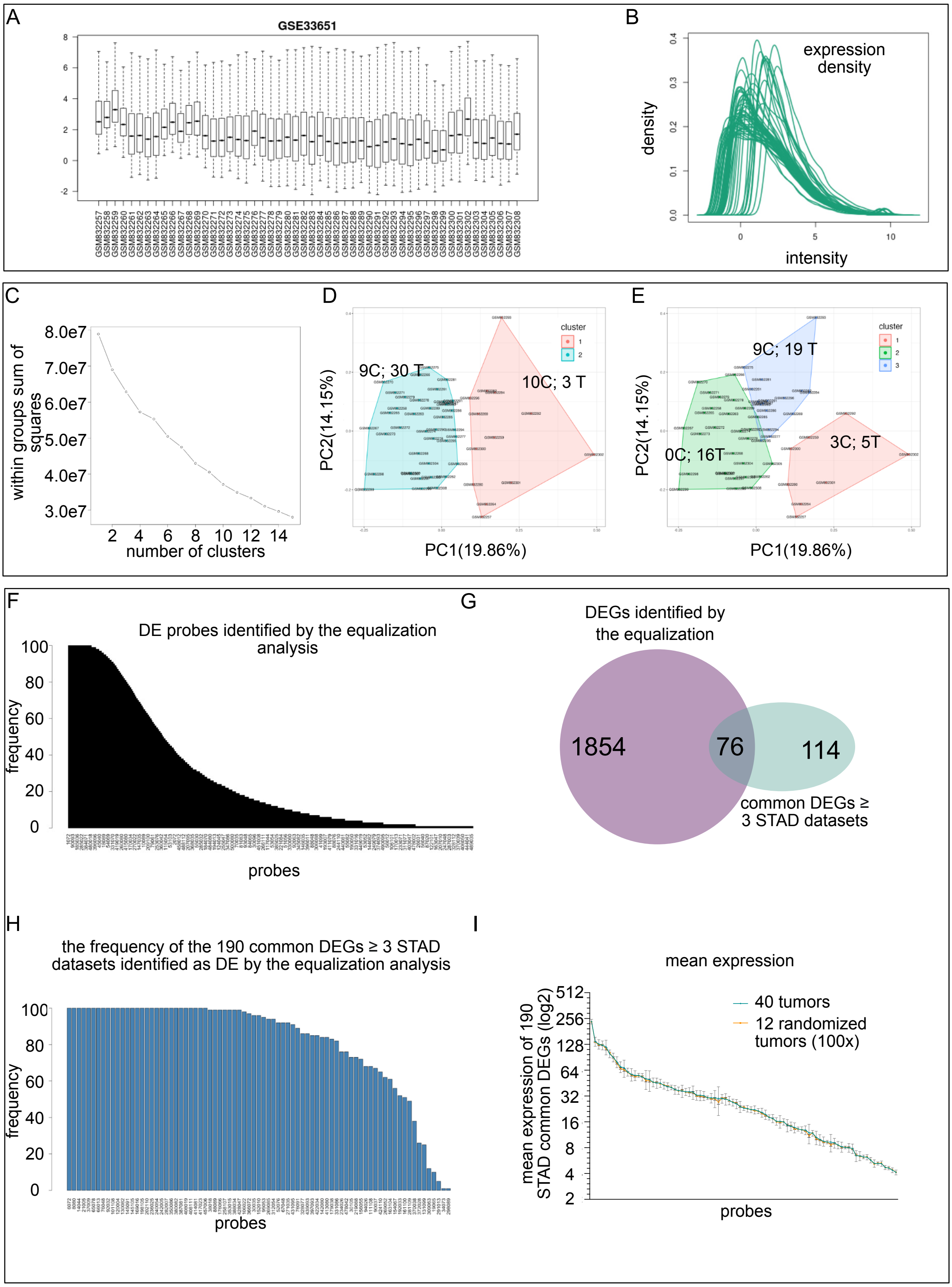

Supplementary Figure 4: Stomach adenocarcinoma dataset GSE33651: The dataset has 40 tumor (T) and 12 control (C) samples. (**A** - **B**) The distribution of the expression values of the samples in dataset GSE33651 are presented as box plot (**A**) and expression density plots (**B**). (**C** - **E**) Unsupervised cluster analysis. The optimal number of clusters (K) is depicted using a Within groups of sum of squares (WSS) plot (**C**). Cluster plots using 2 (**D**) or 3 (**E**) clusters, were generated using the K-means method. In **D** and **E**, the sample identification numbers are shown. (**F - I**) Dataset analysis using group equalization. In this analysis differentially expressed genes were identified using the limma package by comparing the control group (12 controls) versus a randomized tumor group (12 tumors) from the dataset with 100 repetitions. The tumor group was selected randomly for each trial. A graph showing the frequency of the differentially expressed probes within the 100-loop trial is shown (**F**). 190 DEGs were identified as common in at least 3 STAD datasets using GEO2R (from **Figure 2F**). A Venn diagram depicting the intersection between the 190 common DEGs identified in more than 3 STAD datasets compared with DEGs generated using the equalization method in **F** is shown (**G**). A graph depicting the frequency of the probes for the 190 common DEGs identified in more than 3 STAD datasets within the 100-loop trial is shown. (**H**). A graph depicting the mean expression values of the 190 common DEGs in more than 3 STAD datasets in the 40 tumors compared with the mean of the mean expression values in the randomized tumor group using the equalization analysis is shown. The bars represent the standard error of the mean (**I**). Abbreviations: tumor (T), control (C) Within groups of sum of squares (WSS), differentially expressed gene (DEG), differentially expressed (DE), stomach adenocarcinoma (STAD), principle component (PC).

STAD GSE54129: 111 tumors (T); 21 controls (C)

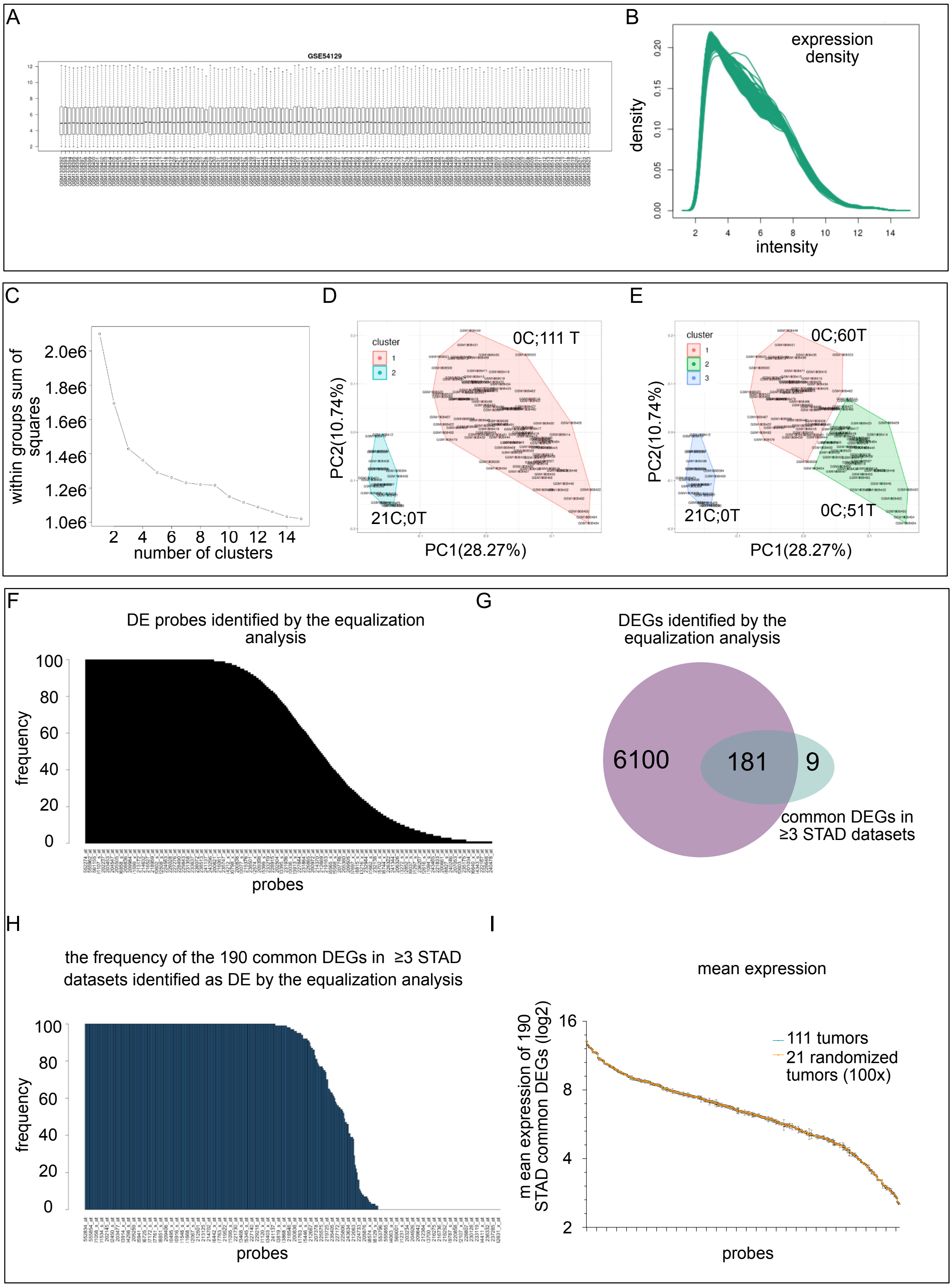

Supplementary Figure 5: Stomach adenocarcinoma dataset GSE54129: The dataset has 111 tumor (T) and 21 control (C) samples. (**A - B**) The distribution of the expression values of the samples in dataset GSE54129 are presented as box plot (**A**) and expression density plots (**B**). (**C - E**) Unsupervised cluster analysis. The optimal number of clusters (K) is depicted using a Within groups of sum of squares (WSS) plot (**C**). Cluster plots using 2 (**D**) or 3 (**E**) clusters, were generated using the K-means method. In **D** and **E**, the sample identification numbers are shown (**F - I**). Dataset analysis using group equalization. In this analysis differentially expressed genes were identified using the limma package by comparing the control group (21 controls) versus a randomized tumor group (21 tumors) from the dataset with 100 repetitions. The tumor group was selected randomly for each trial. A graph showing the frequency of the differentially expressed probes within the 100-loop trial is shown (**F**). 190 DEGs were identified as common in at least 3 STAD datasets using GEO2R (from **Figure 2F**). A Venn diagram depicting the intersection between the 190 common DEGs identified in more than 3 STAD datasets compared with DEGs generated using the equalization method in **F** is shown (**G**). A graph depicting the frequency of the probes for the 190 common DEGs identified in more than 3 STAD datasets within the 100-loop trial is shown (**H**). A graph depicting the mean expression values of the 190 common DEGs in more than 3 STAD datasets in the 111 tumors compared with the mean of the mean expression values in the randomized tumor group using the equalization analysis is shown. The bars represent the standard error of the mean (**I**). Abbreviations: tumor (T), control (C) Within groups of sum of squares (WSS), differentially expressed gene (DEG), differentially expressed (DE), stomach adenocarcinoma (STAD), principle component (PC).

HNSCC GSE138206: 6 tumors (T); 6 controls (C)

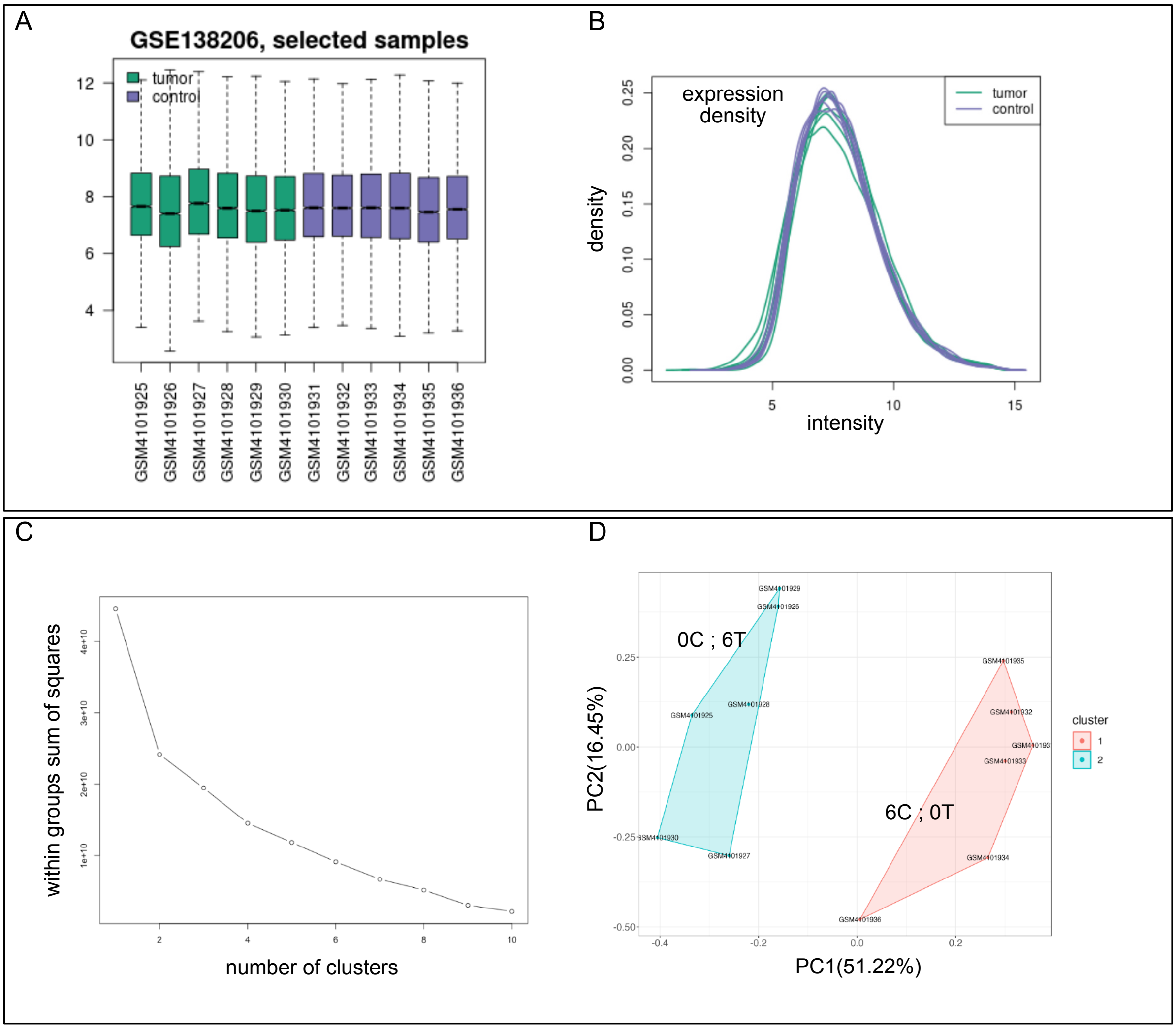

Supplementary Figure 6: Head and neck squamous cell carcinoma dataset GSE138206: The dataset has 6 tumor (T) and 6 control (C) samples (6 adjacent tissue controls were excluded in this analysis). (**A** - **B**) The distribution of the expression values of the samples in dataset GSE138206 are presented as box plot (**A**) and expression density plots (**B**). (**C** - **D**) Unsupervised cluster analysis. The optimal number of clusters (K) is depicted using a Within groups of sum of squares (WSS) plot (**C**). Cluster plots using 2 (**D**) clusters, was generated using the K-means method. In **D** the sample identification numbers are shown. Abbreviations: tumor (T), control (C) Within groups of sum of squares (WSS), differentially expressed gene (DEG), head and neck squamous cell carcinoma (HNSCC), principle component (PC).

Supplementary Figure 7

HNSCC GSE6631: 22 tumors (T); 22 controls (C)

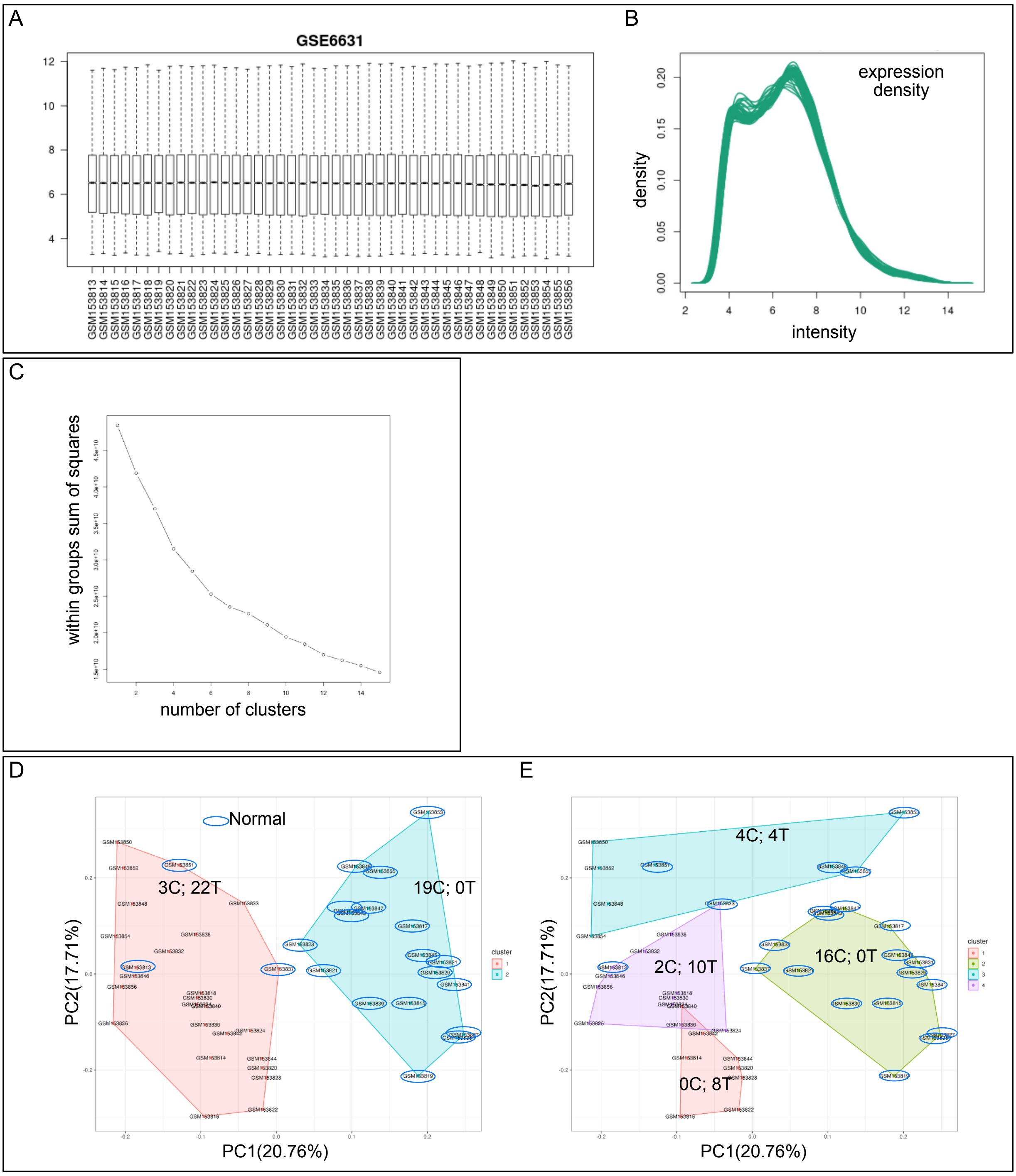

Supplementary Figure 7: Head and neck squamous cell carcinoma dataset GSE6631: The dataset has 22 tumor (T) and 22 control (C) samples. (**A** - **B**) The distribution of the expression values of the samples in dataset GSE6631 are presented as box plot (**A**) and expression density plots (**B**). (**C** - **E**) Unsupervised cluster analysis. The optimal number of clusters (K) is depicted using a Within groups of sum of squares (WSS) plot (**C**). Cluster plots using 2 (**D**) or 4 (**E**) clusters, were generated using the K-means method. In **D** and **E**, the sample identification numbers are shown and the samples circled in blue are control samples. Abbreviations: tumor (T), control (C) Within groups of sum of squares (WSS), differentially expressed gene (DEG), head and neck squamous cell carcinoma (HNSCC), principle component (PC).

PDAC GSE15471: 39 tumors (T); 39 controls (C)

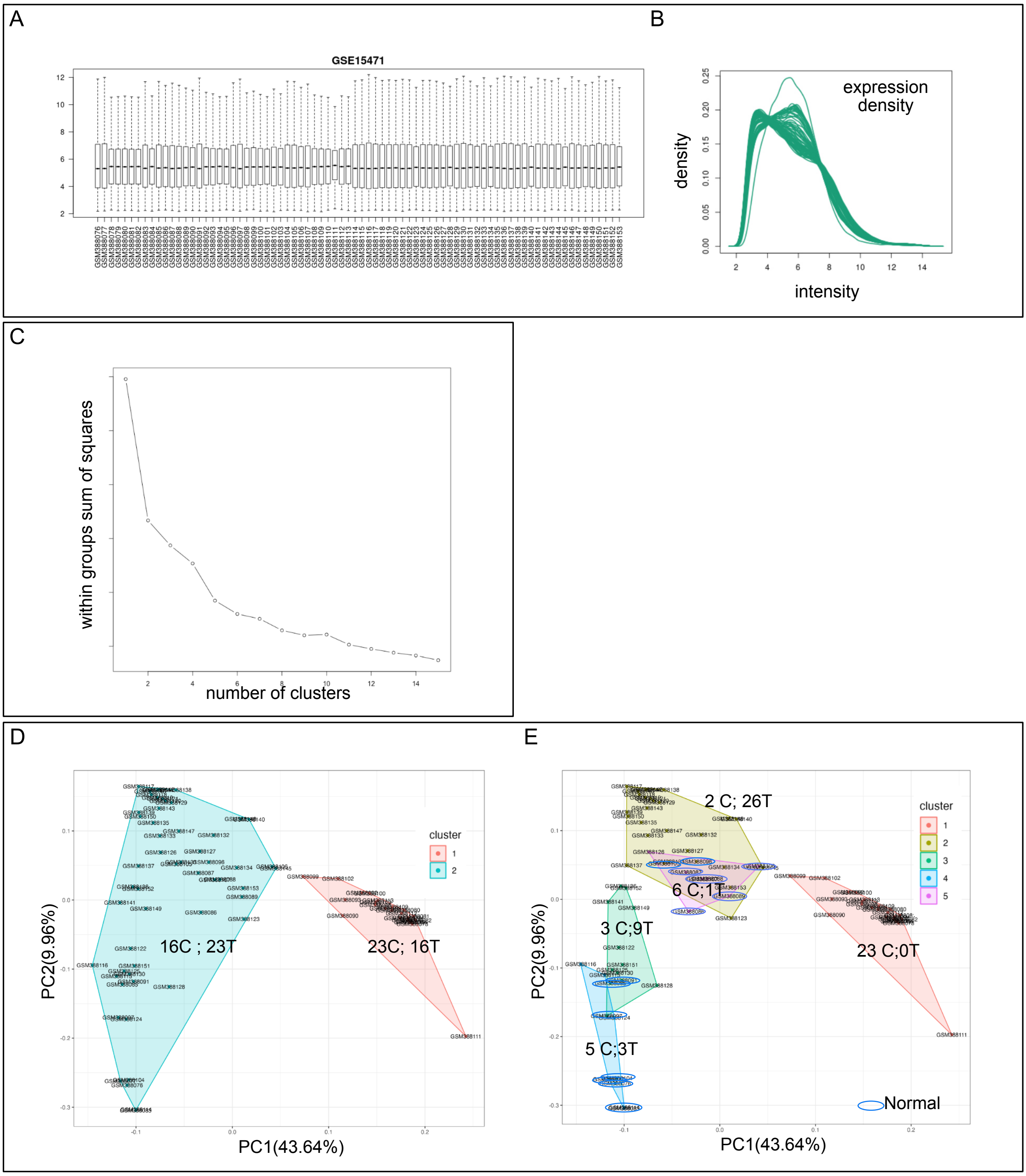

Supplementary Figure 8: Pancreatic ductal adenocarcinoma dataset GSE15471: The dataset has 39 tumor (T) and 39 control (C) samples. (**A** - **B**) The distribution of the expression values of the samples in dataset GSE15471 are presented as box plot (**A**) and expression density plots (**B**). (**C** - **E**) Unsupervised cluster analysis. The optimal number of clusters (K) is depicted using a Within groups of sum of squares (WSS) plot (**C**). Cluster plots using 2 (**D**) or 5 (**E**) clusters, were generated using the K-means method. In **D** and **E**, the sample identification numbers are shown and the samples circled in blue are control samples. Abbreviations: tumor (T), control (C) Within groups of sum of squares (WSS), differentially expressed gene (DEG), pancreatic ductal adenocarcinoma (PDAC), principle component (PC).

Supplementary Figure 9

PDAC GSE62452: 69 tumors(T); 61 controls (C)

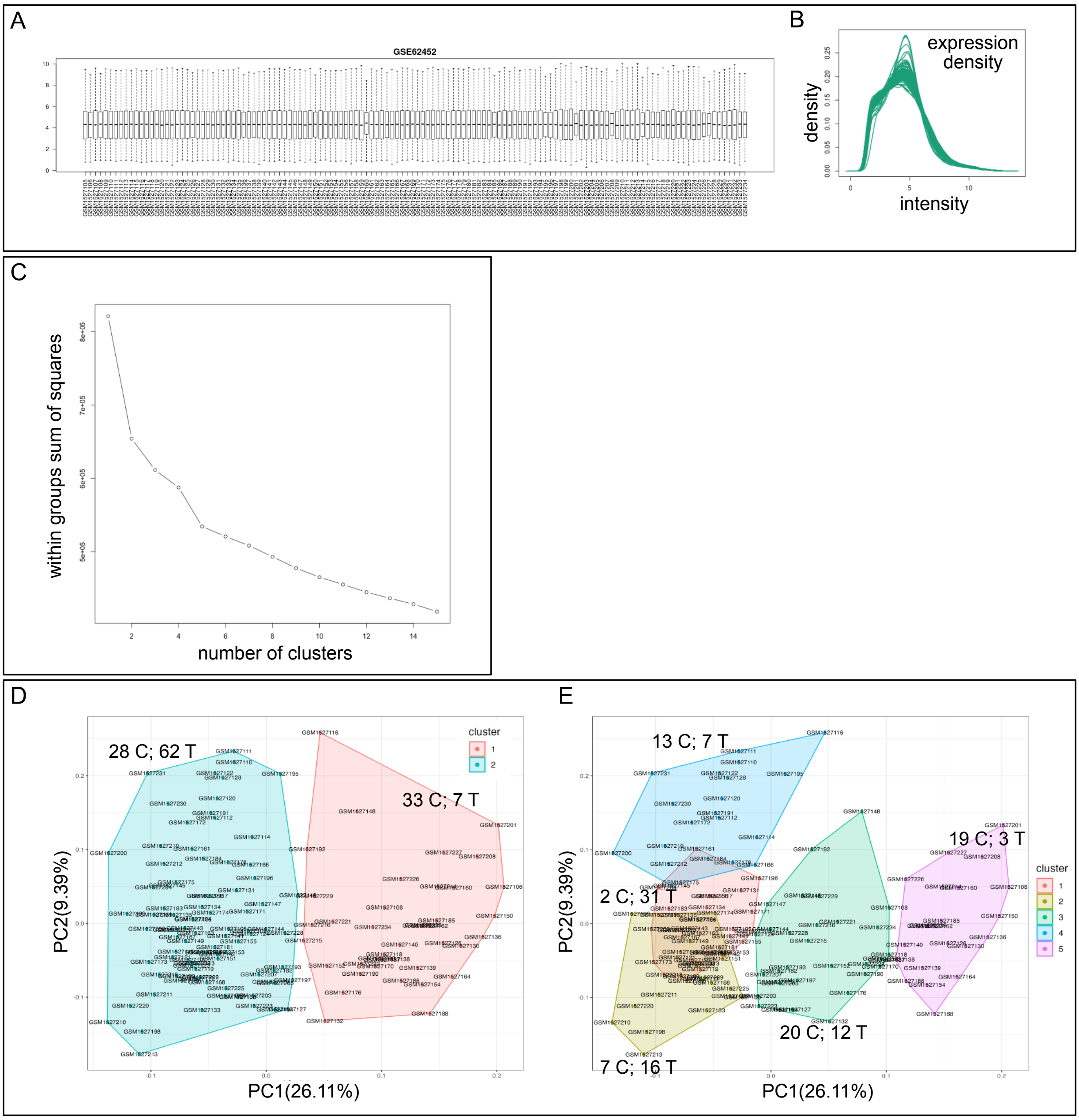

Supplementary Figure 9: Pancreatic ductal adenocarcinoma dataset GSE62452: The dataset has 69 tumor (T) and 61 control (C) samples. (**A** - **B**) The distribution of the expression values of the samples in dataset GSE62452 are presented as box plot (**A**) and expression density plots (**B**). (**C** - **E**) Unsupervised cluster analysis. The optimal number of clusters (K) is depicted using a Within groups of sum of squares (WSS) plot (**C**). Cluster plots using 2 (**D**) or 5 (**E**) clusters, were generated using the K-means method. In **D** and **E**, the sample identification numbers are shown. Abbreviations: tumor (T), control (C) Within groups of sum of squares (WSS), differentially expressed gene (DEG), pancreatic ductal adenocarcinoma (PDAC), principle component (PC).

PDAC GSE28735: 45 tumors (T); 45 controls (C)

Supplementary Figure 10

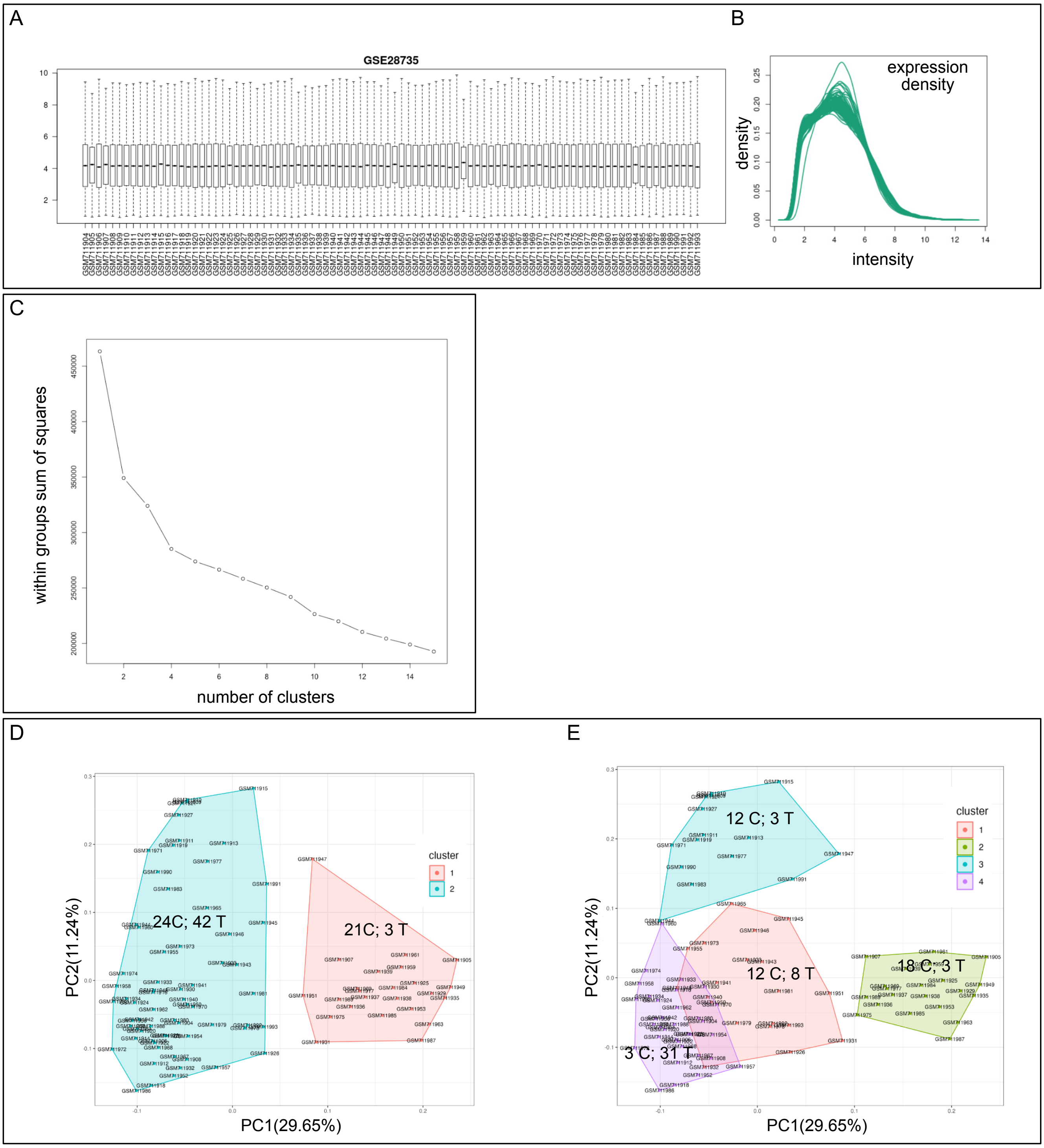

Supplementary Figure 10: Pancreatic ductal adenocarcinoma dataset GSE28735: The dataset has 45 tumor (T) and 45 control (C) samples. (**A** - **B**) The distribution of the expression values of the samples in dataset GSE28735 are presented as box plot (**A**) and expression density plots (**B**). (**C** - **E**) Unsupervised cluster analysis. The optimal number of clusters (K) is depicted using a Within groups of sum of squares (WSS) plot (**C**). Cluster plots using 2 (**D**) or 4 (**E**) clusters, were generated using the K-means method. Abbreviations: tumor (T), control (C) Within groups of sum of squares (WSS), differentially expressed gene (DEG), pancreatic ductal adenocarcinoma (PDAC), principle component (PC).

Supplementary Figure 11

STAD GSE103236: 10 tumors (T); 9 controls (C)

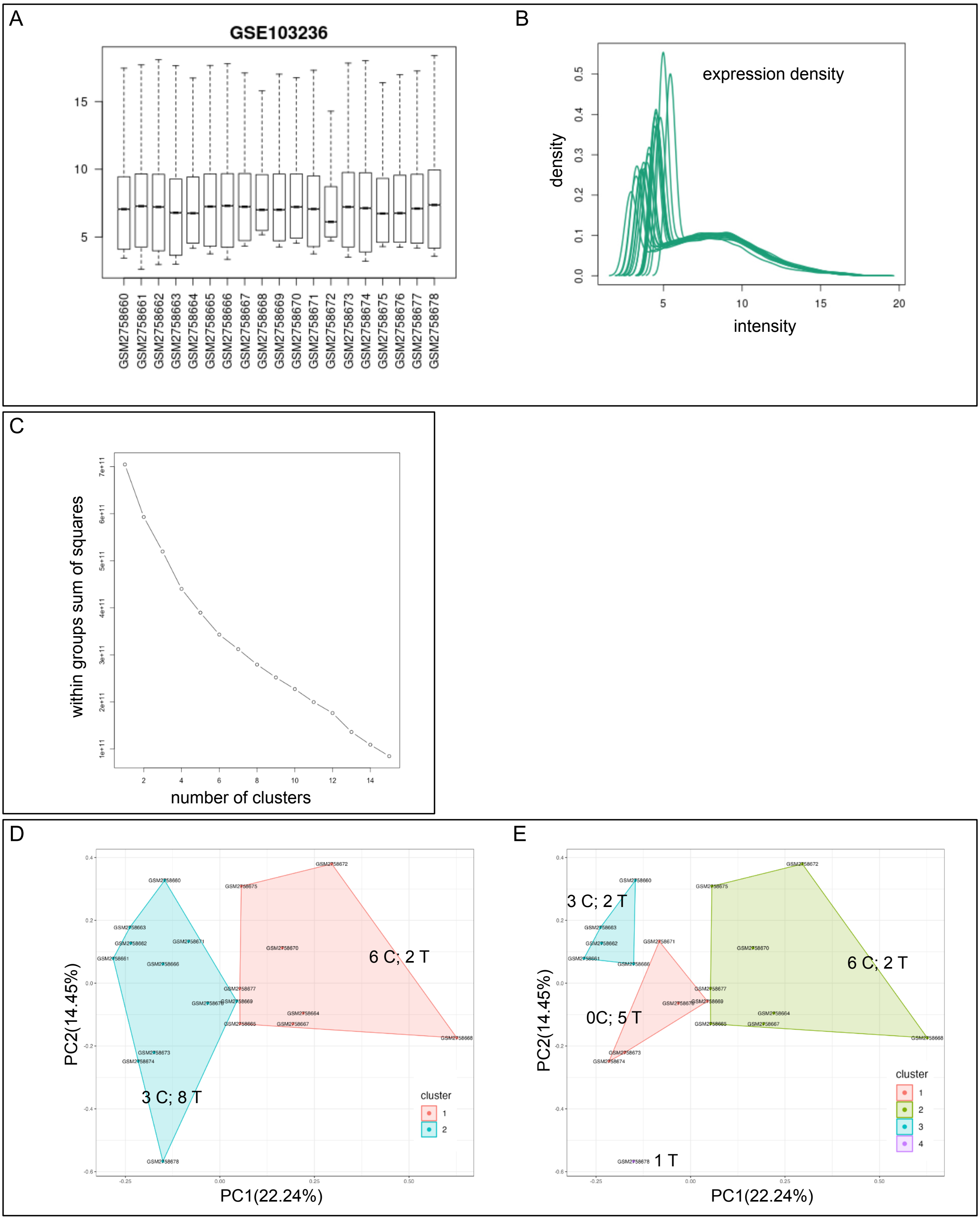

Supplementary Figure 11: Stomach adenocarcinoma dataset GSE103236: The dataset has 10 tumor (T) and 9 control (C) samples. (**A - B**) The distribution of the expression values of the samples in dataset GSE103236 are presented as box plot (**A**) and expression density plots (**B**). (**C - E**) Unsupervised cluster analysis. The optimal number of clusters (K) is depicted using a Within groups of sum of squares (WSS) plot (**C**). Cluster plots using 2 (**D**) or 4 (**E**) clusters, were generated using the K-means method. In **D** and **E**, the sample identification numbers are shown. Abbreviations: tumor (T), control (C) Within groups of sum of squares (WSS), differentially expressed gene (DEG), stomach adenocarcinoma (STAD), principle component (PC).

Supplementary Figure 12

STAD GSE65801: 32 tumors (T); 32 controls (C)

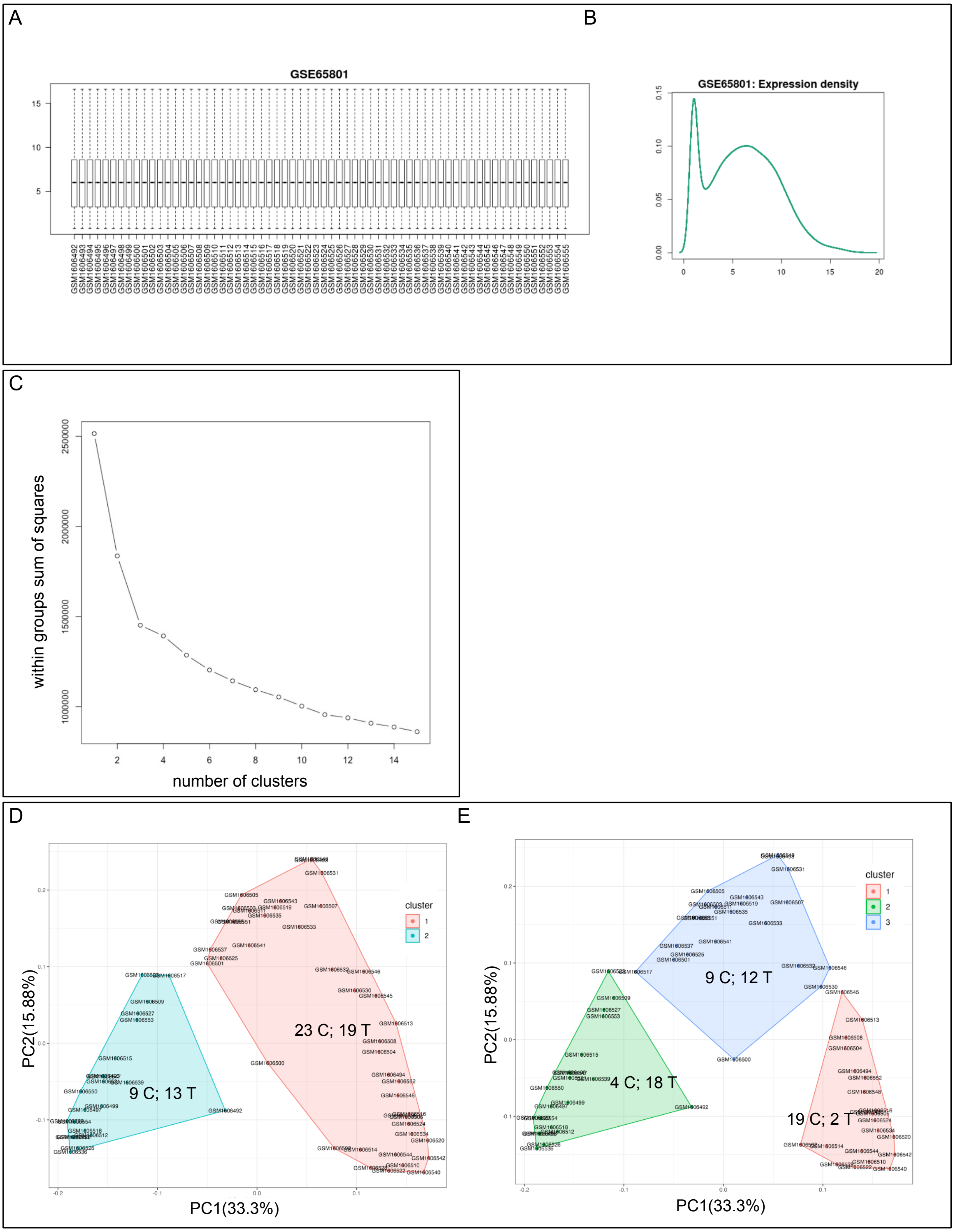

Supplementary Figure 12: Stomach adenocarcinoma dataset GSE65801: The dataset has 32 tumor (T) and 32 control (C) samples. (**A** - **B**) The distribution of the expression values of the samples in dataset GSE65801 are presented as box plot (**A**) and expression density plots (**B**). (**C** - **E**) Unsupervised cluster analysis. The optimal number of clusters (K) is depicted using a Within groups of sum of squares (WSS) plot (**C**). Cluster plots using 2 (**D**) or 3 (**E**) clusters, were generated using the K-means method. In **D** and **E**, the sample identification numbers are shown. Abbreviations: tumor (T), control (C) Within groups of sum of squares (WSS), differentially expressed gene (DEG), stomach adenocarcinoma (STAD), principle component (PC).

STAD GSE79973: 10 tumors (T); 10 controls (C)

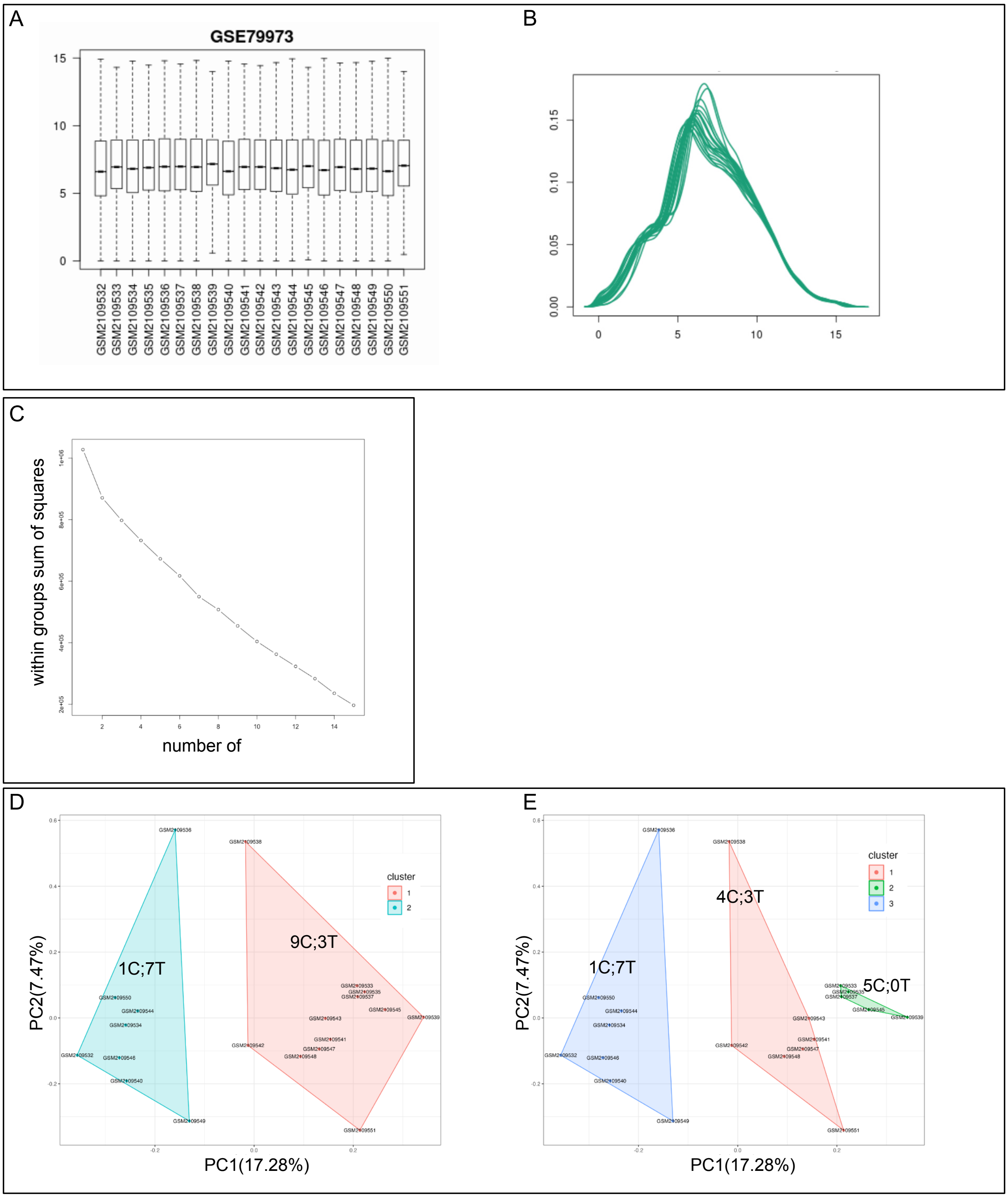

Supplementary Figure 13: Stomach adenocarcinoma dataset GSE79973: The dataset has 10 tumor (T) and 10 control (C) samples. (**A** - **B**) The distribution of the expression values of the samples in dataset GSE79973 are presented as box plot (**A**) and expression density plots (**B**). (**C** - **E**) Unsupervised cluster analysis. The optimal number of clusters (K) is depicted using a Within groups of sum of squares (WSS) plot (**C**). Cluster plots using 2 (**D**) or 3 (**E**) clusters, were generated using the K-means method. In **D** and **E**, the sample identification numbers are shown. Abbreviations: tumor (T), control (C) Within groups of sum of squares (WSS), differentially expressed gene (DEG), stomach adenocarcinoma (STAD), principle component (PC).

Supplementary Figure 14

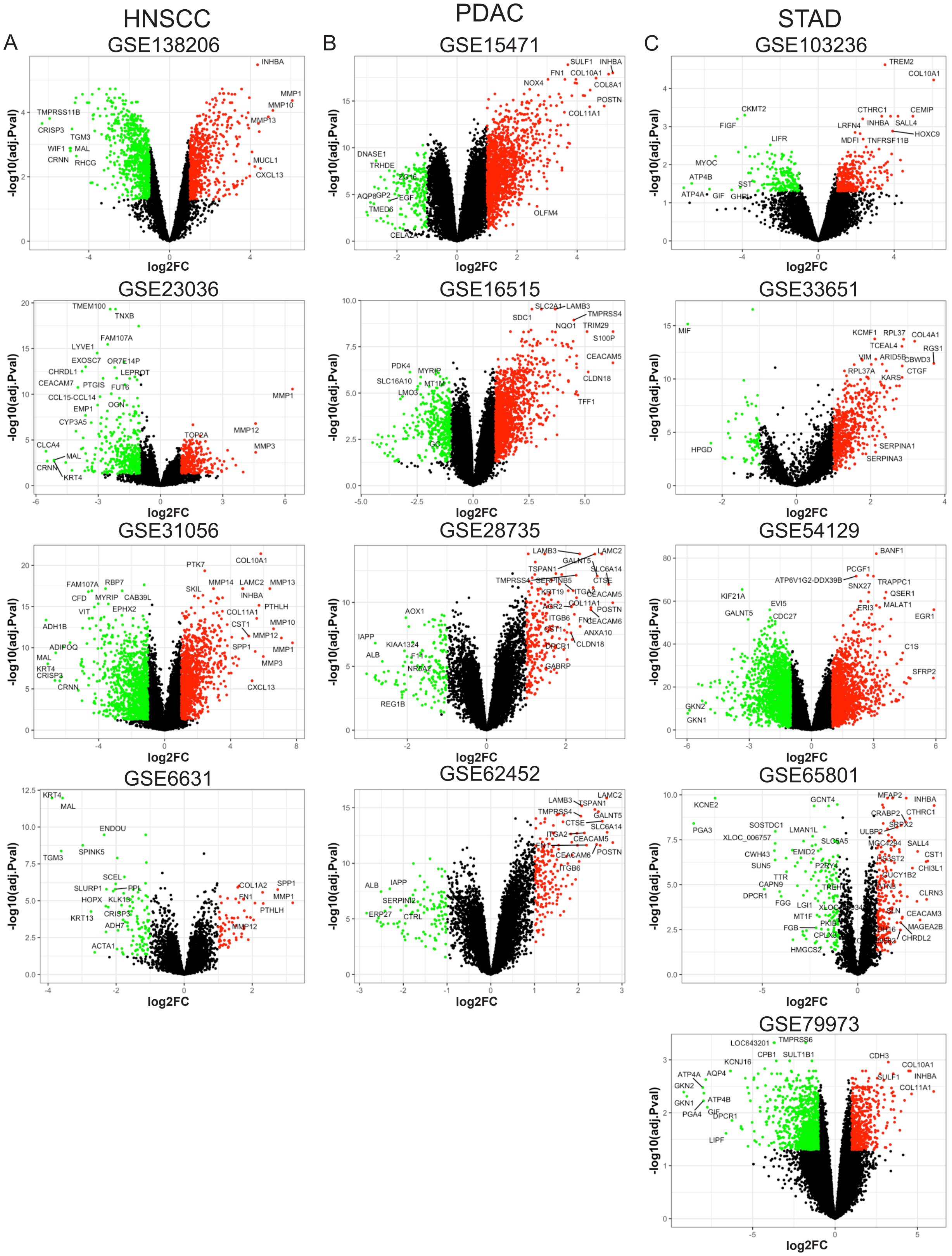

Supplementary Figure 14: Volcano plots showing the distribution of DEGs from the GEO datasets. (**A**) Head and neck squamous cell carcinoma (HNSCC), (**B**) Pancreatic ductal adenocarcinoma (PDAC), (**C**) and gastric adenocarcinoma (STAD) datasets. The dataset identification number is indicated above the graph for that dataset. The X-axis indicates the log2FC and the Y-axis the -log10(adj. p-value). Each dot represents a gene. Red depicts upregulated genes with log2FC >1 & adj. p-value <0.05; green depicts downregulated genes with a log2FC < -1 & adj. p-value <0.05. Black dots represent genes that are either below threshold for fold change, statistical significance or both. Abbreviations: DEG, differentially expressed gene; HNSCC, head and neck squamous cell carcinoma ; PDAC, pancreatic ductal adenocarcinoma; STAD, stomach adenocarcinoma; FC, fold change.

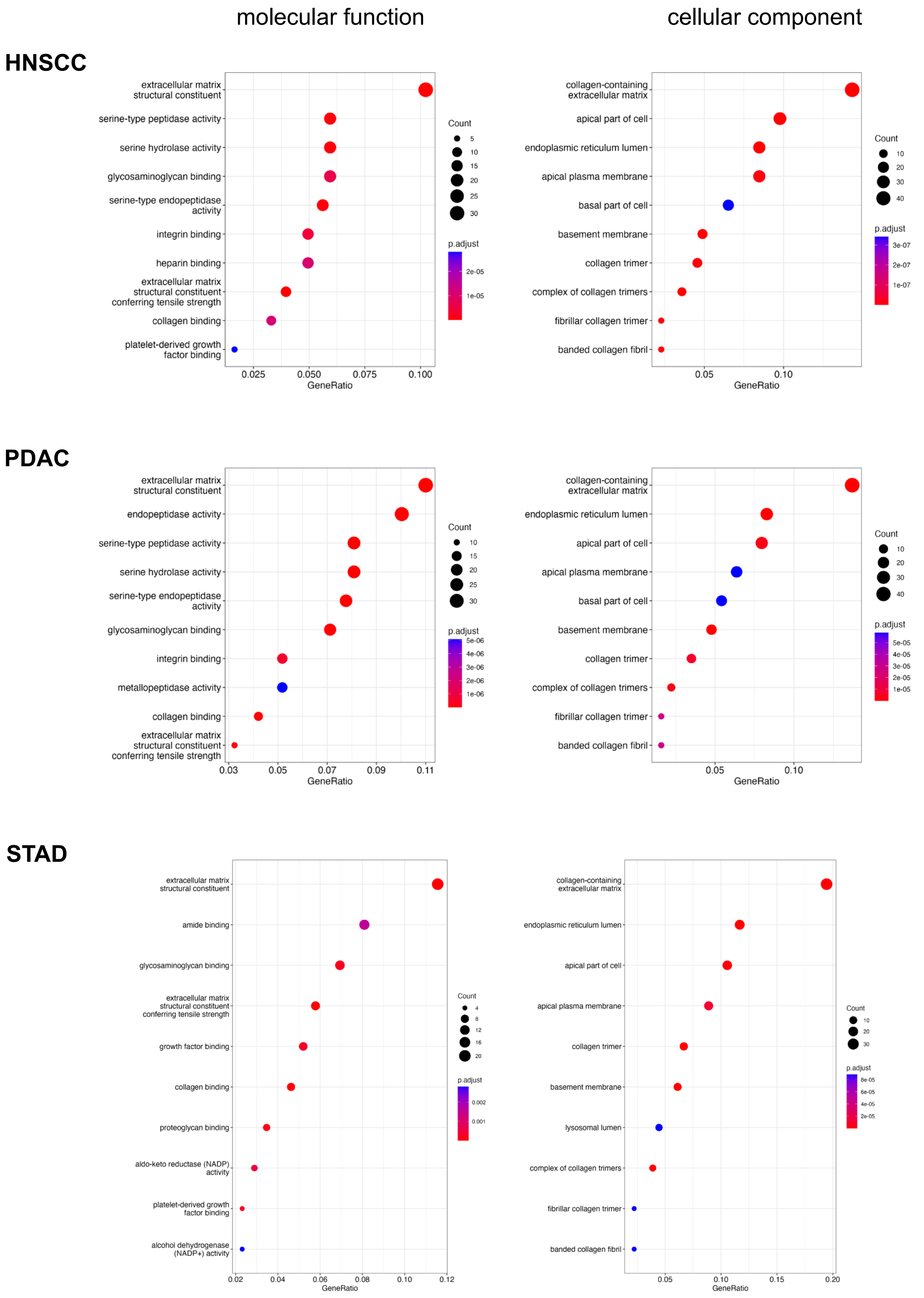
molecular function
cellular component
HNSCC
extracellular matrix structural constituent
serine-type peptidase activity
serine hydrolase activity
glycosaminoglycan binding
serine-type endopeptidase activity
integrin binding
heparin binding
extracellular matrix structural constituent conferring tensile strength
collagen binding
platelet-derived growth factor binding
GeneRatio
Count
p.adjust
collagen-containing extracellular matrix
apical part of cell
endoplasmic reticulum lumen
apical plasma membrane
basal part of cell
basement membrane
collagen trimer
complex of collagen trimers
fibrillar collagen trimer
banded collagen fibril
PDAC
endopeptidase activity
metallopeptidase activity
STAD
amide binding
growth factor binding
proteoglycan binding
aldo-keto reductase (NADP) activity
alcohol dehydrogenase (NADP+) activity
lysosomal lumen

Supplementary Figure 15: Gene ontology analysis for the GEO datasets head and neck squamous cell carcinoma (HNSCC), pancreatic ductal adenocarcinoma (PDAC) and stomach adenocarcinoma (STAD). Gene ontology terms for molecular functions and cellular compartments are shown. The corresponding terms for biological processes are shown in **Figure 2I-K**. Differentially expressed genes (DEGs) common in at least 3 different datasets for each cancer were used to performed a gene ontology (GO) analysis. The GO analysis was conducted using the ClusterProfiler from R software and top 10 enriched terms determined by p-value <0.05 are depicted. The X-axis indicates the gene ratio and the Y-axis the ranked terms. Abbreviations: HNSCC, head and neck squamous cell carcinoma; PDAC, pancreatic ductal adenocarcinoma; STAD, stomach adenocarcinoma.

Supplementary Figure 16

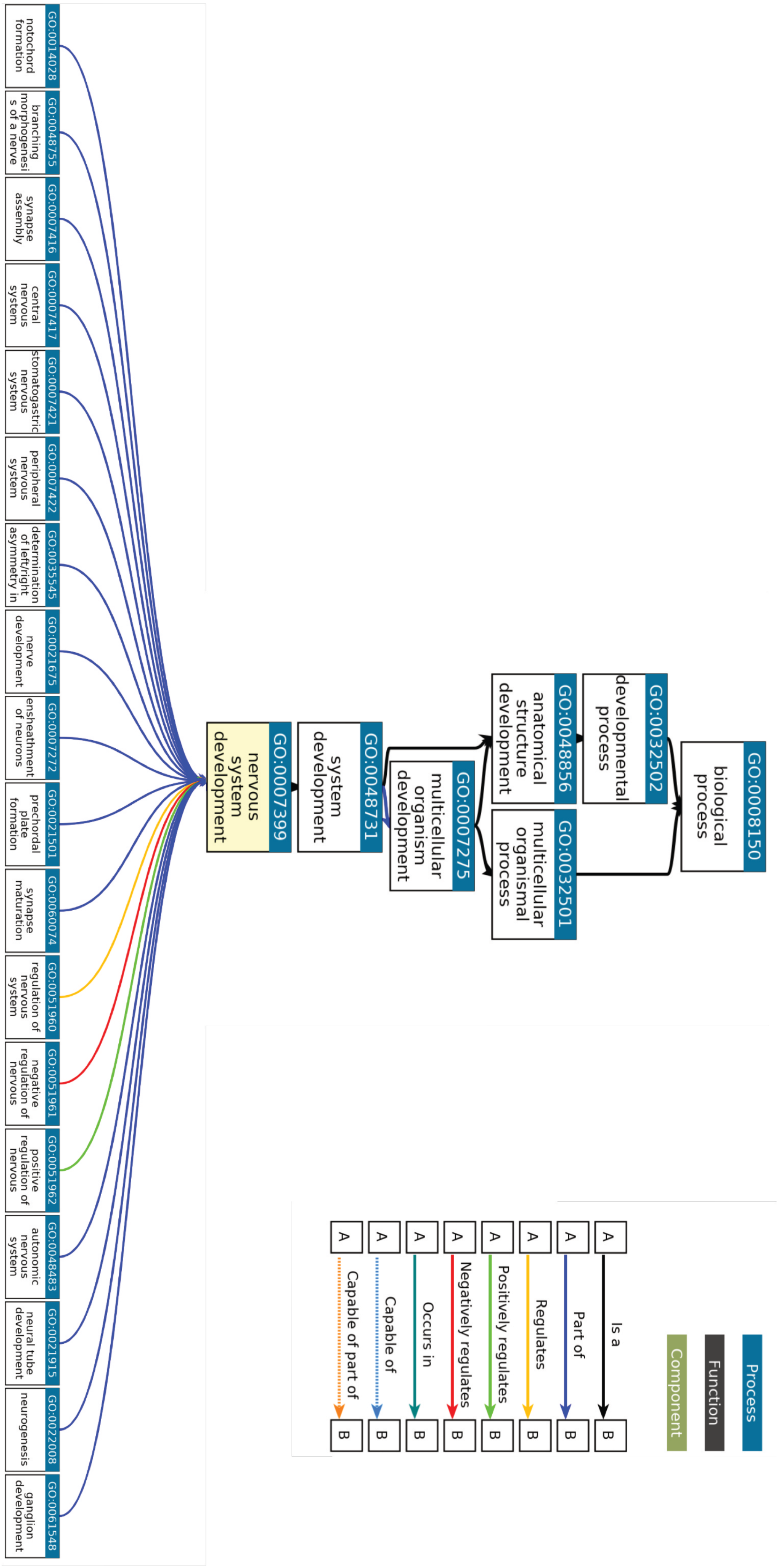

Supplementary Figure 16: Ancestor chart for neurodevelopmental gene signature GO:0007399 used in this study. The repository QuickGO was used to construct a neurodevelopmental gene signature (yellow box). Boxes above nervous system development are considered “ancestors” and boxes below are considered “children”. This chart was generated in QuickGo.

A

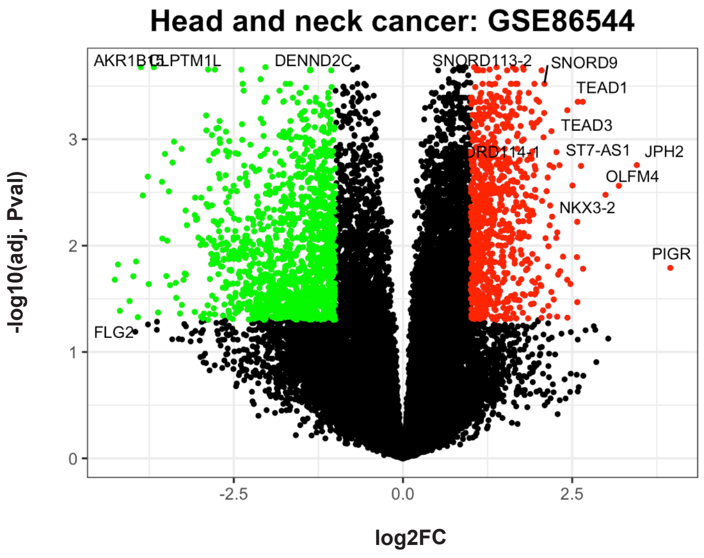
Head and neck cancer: GSE86544
AKR1B15
CLPTM1L
DENND2C
SNORD113-2
SNORD9
TEAD1
TEAD3
ST7-AS1
JPH2
OLFM4
NKX3-2
PIGR
FLG2
-log10(adj. Pval)
log2FC
3
2
1
0
-2.5
0.0
2.5

B

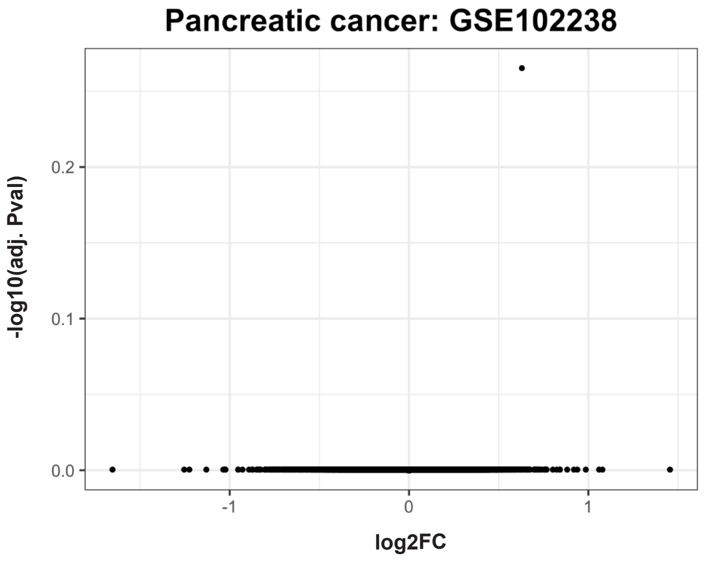
Pancreatic cancer: GSE102238
-log10(adj. Pval)
log2FC
0.2
0.1
0.0
-1
0
1

Supplementary Figure 17: Volcano plots showing the distribution of DEGs from the PNI datasets in PDAC and HNSCC. (**A**) head and neck squamous cell carcinoma (HNSCC) dataset GSE86544 (15 PNI tumors and 9 no PNI tumors) and (**B**) Pancreatic ductal adenocarcinoma (PDAC) dataset GSE102238 (28 PNI tumors and 22 no PNI tumors). Differentially expressed genes were identified by comparing gene expression between PNI tumor and no PNI tumors. The X-axis indicates the log2FC and the Y-axis the -log10(adj. p-value). Each dot represents a gene. Red depicts upregulated genes with log2FC >1 & adj. p-value <0.05; green depicts downregulated genes with a log2FC < -1 & adj. p-value <0.05. Black dots represent genes that are either below threshold for fold change, statistical significance or both. Abbreviations: DEG, differentially expressed gene; HNSCC, Head and Neck Squamous Cell Carcinoma; PDAC, pancreatic ductal adenocarcinoma; FC, fold change.

Supplementary Figure 18

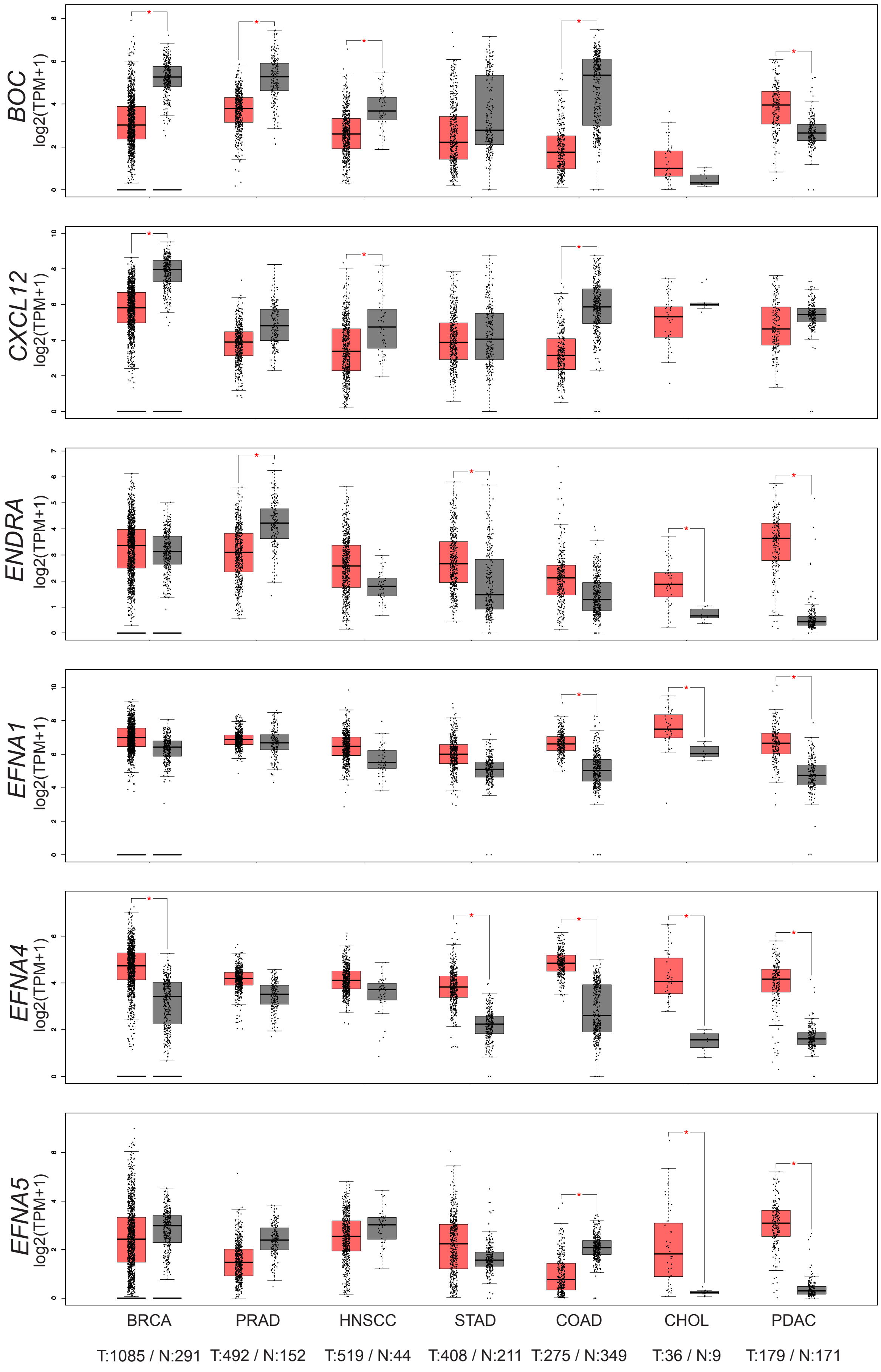

Supplementary Figure 18 continued

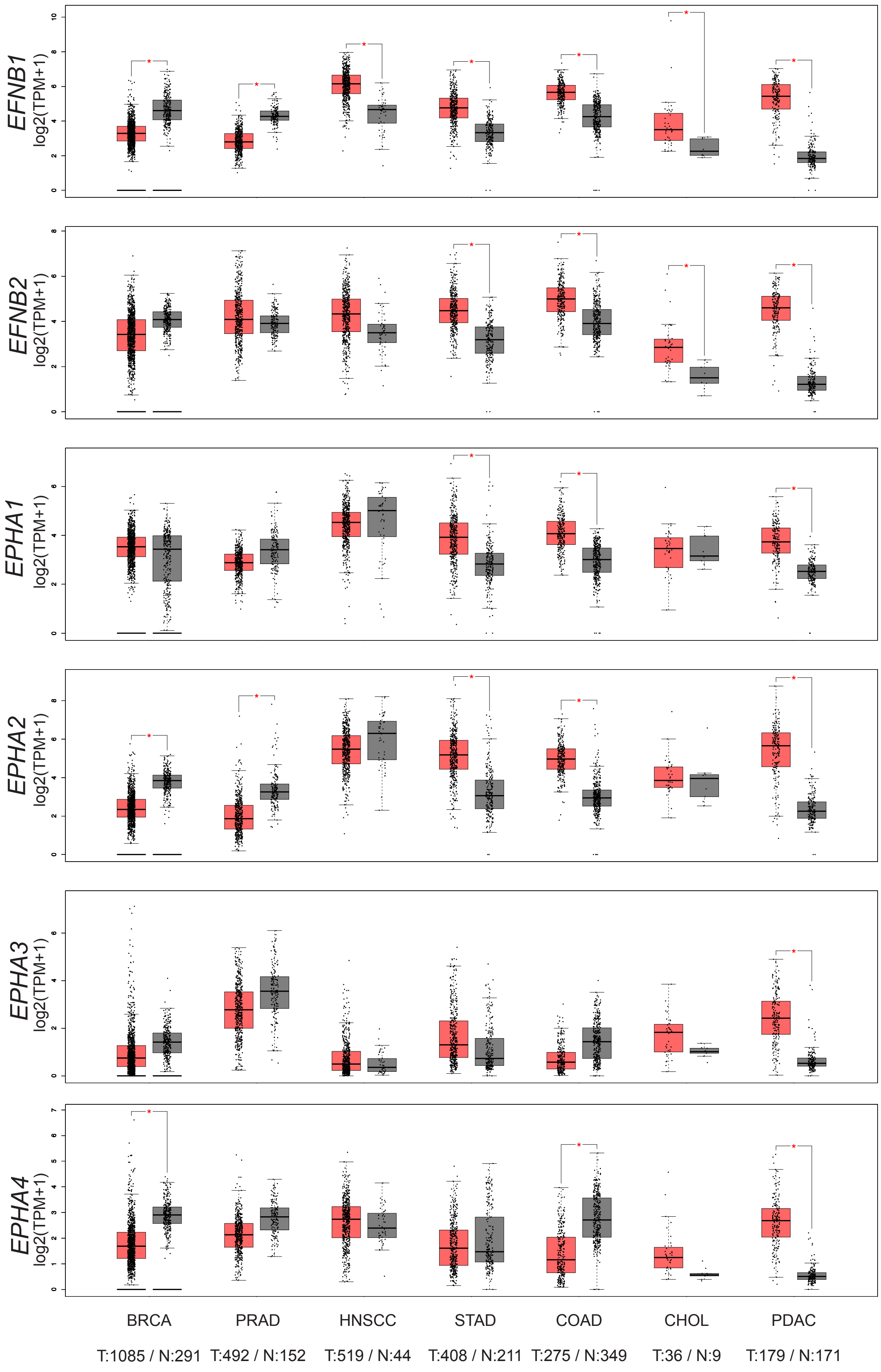

Supplementary Figure 18 continued

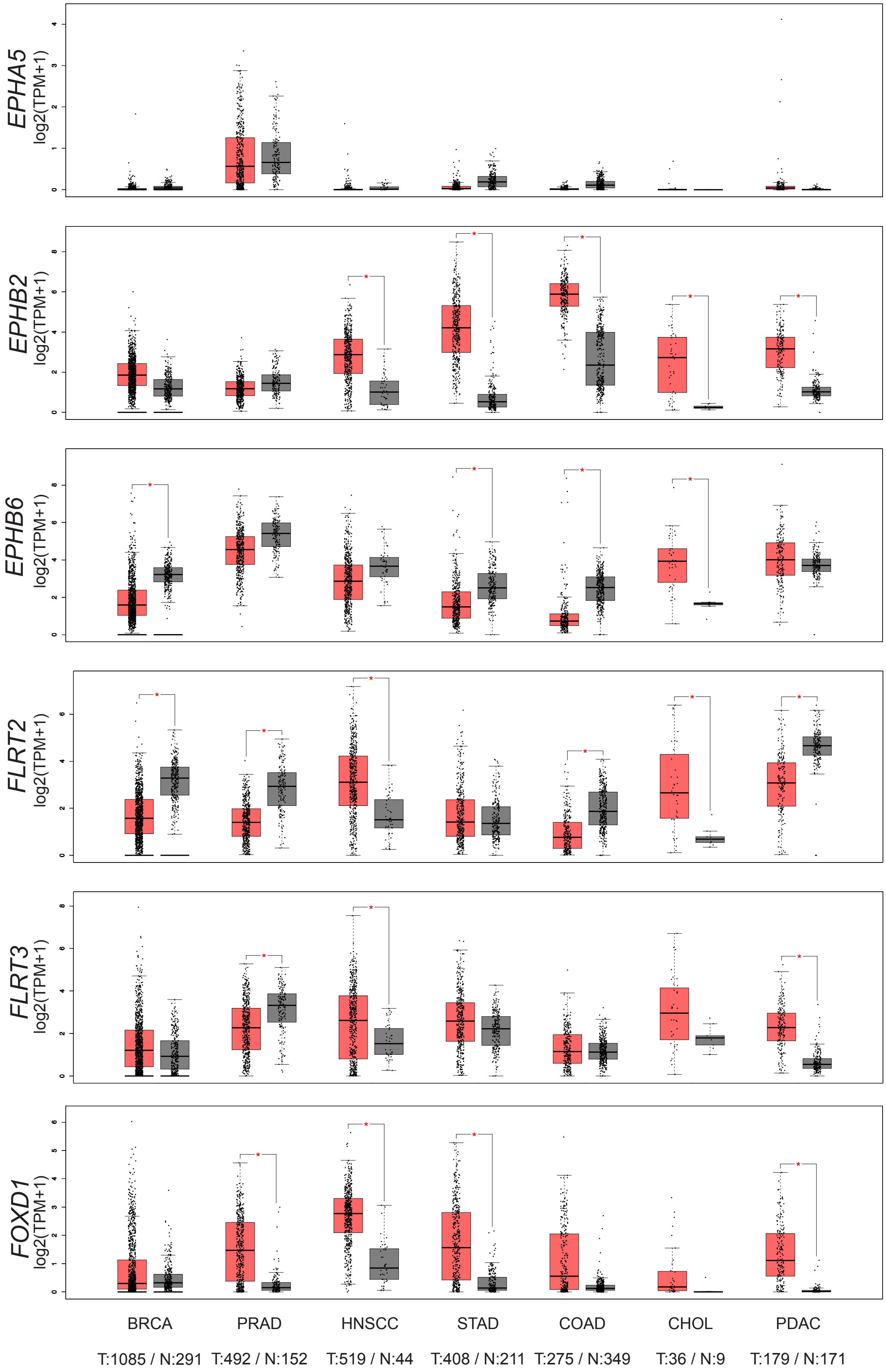

Supplementary Figure 18 continued

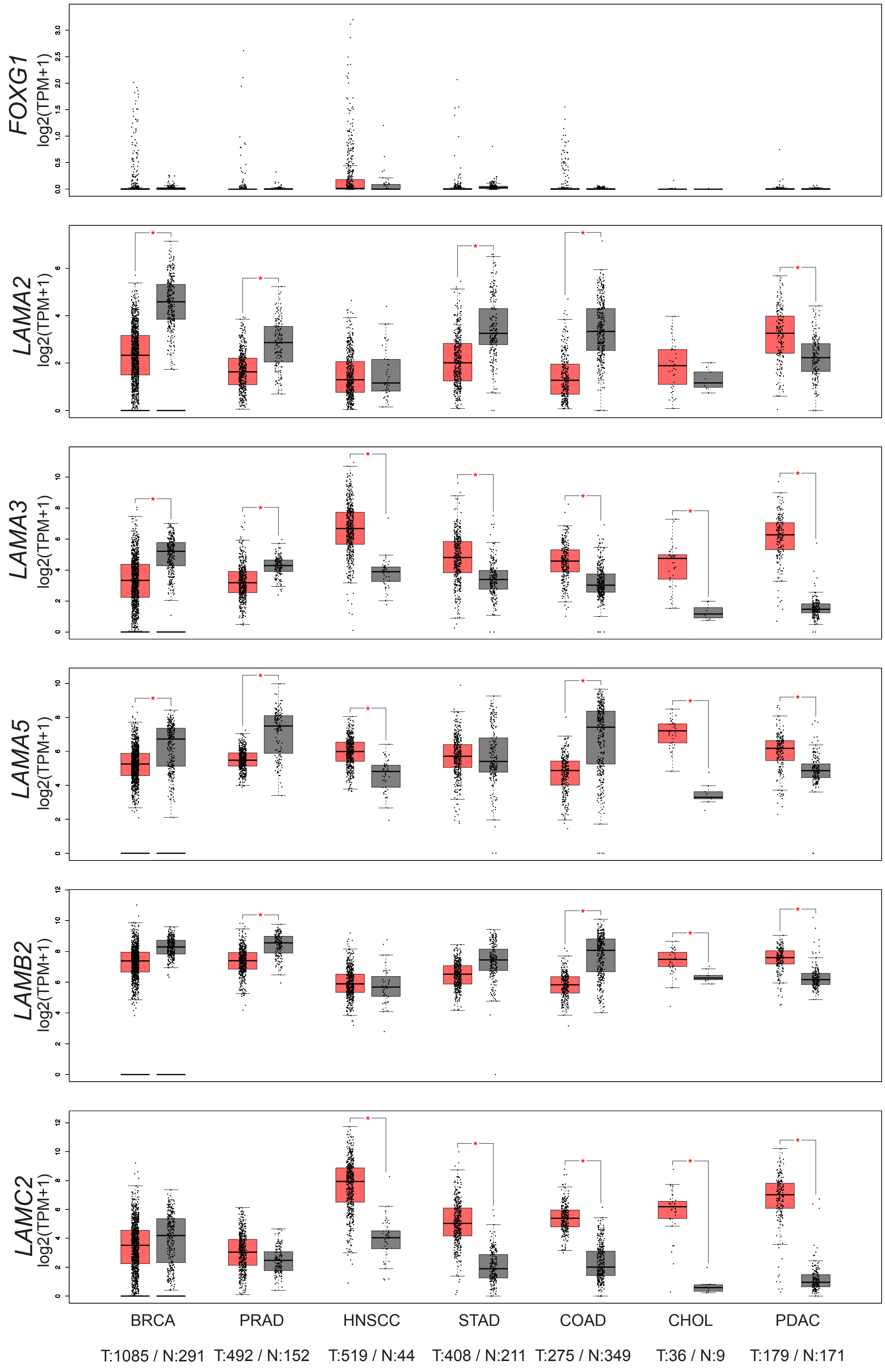

Supplementary Figure 18 continued

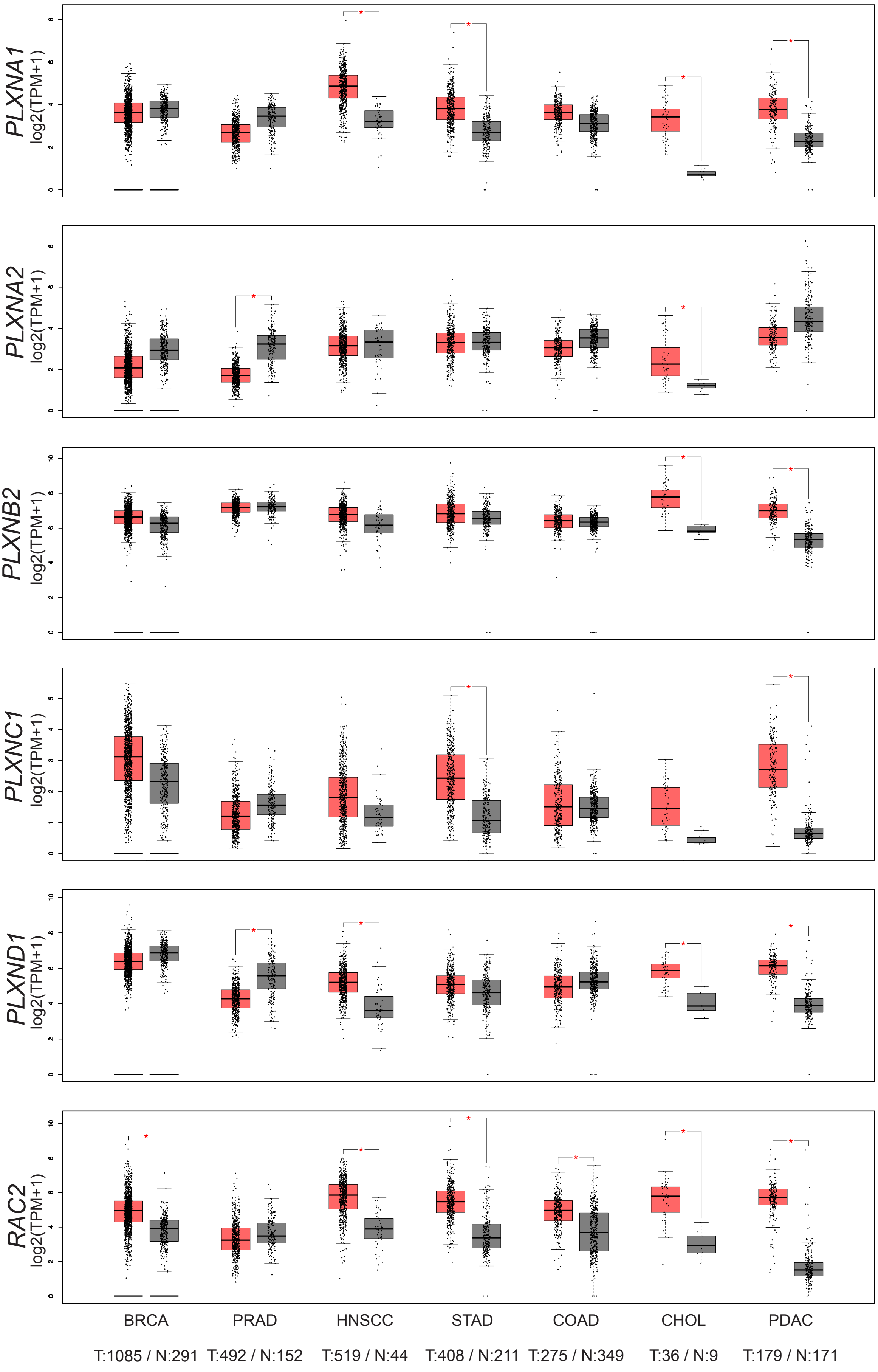

Supplementary Figure 18 continued

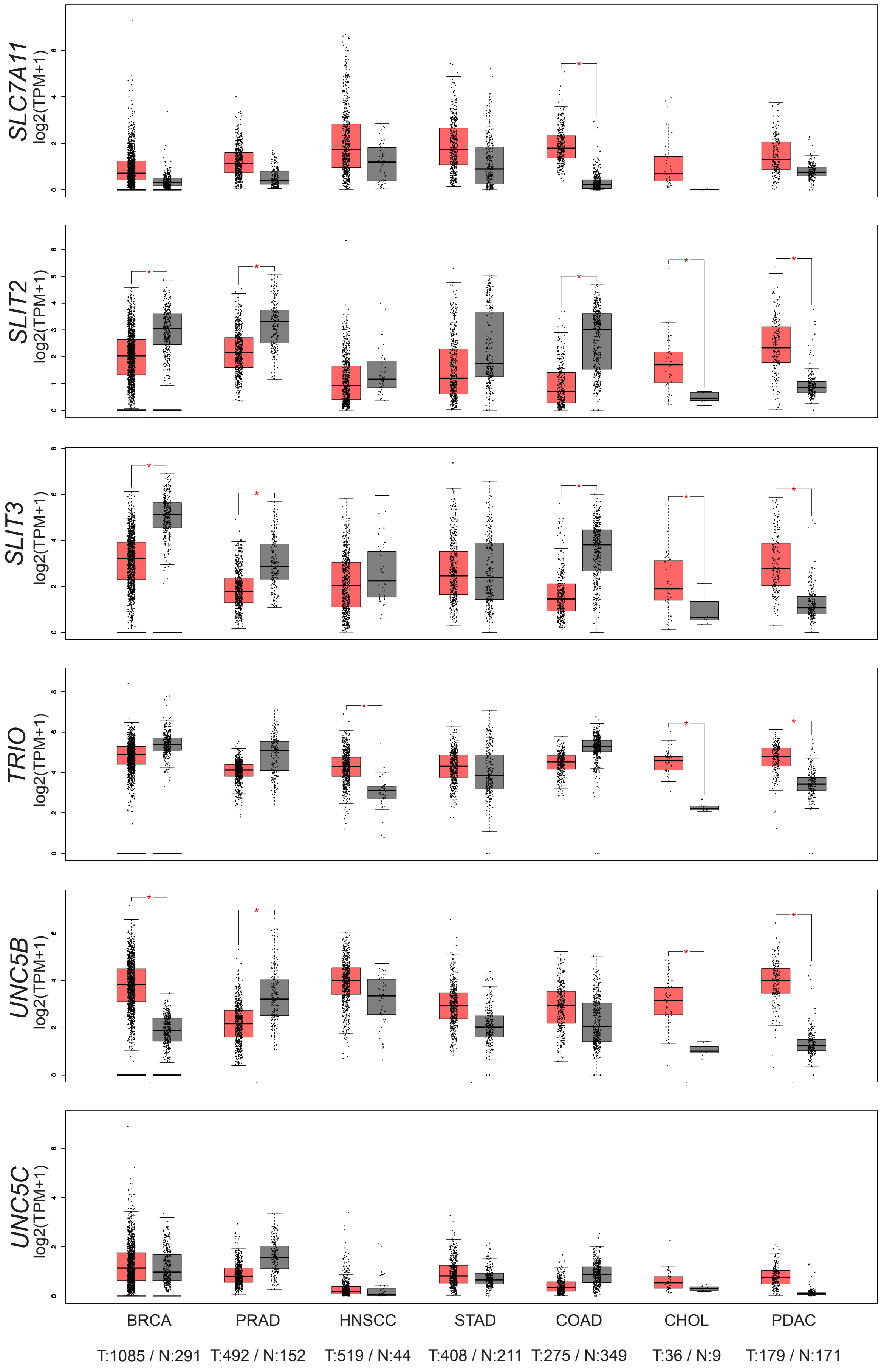

Supplementary Figure 18 continued

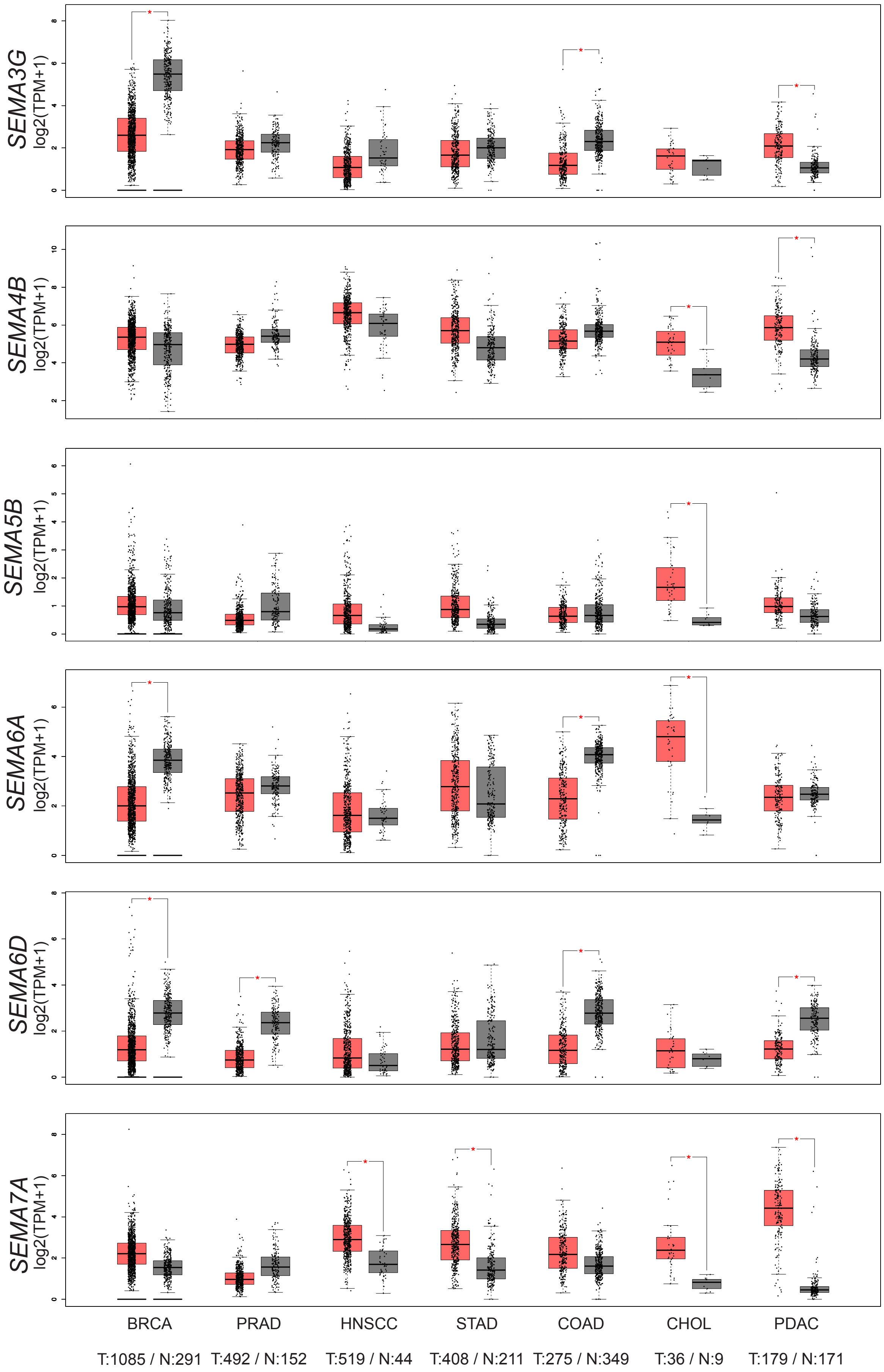

Supplementary Figure S18 continued

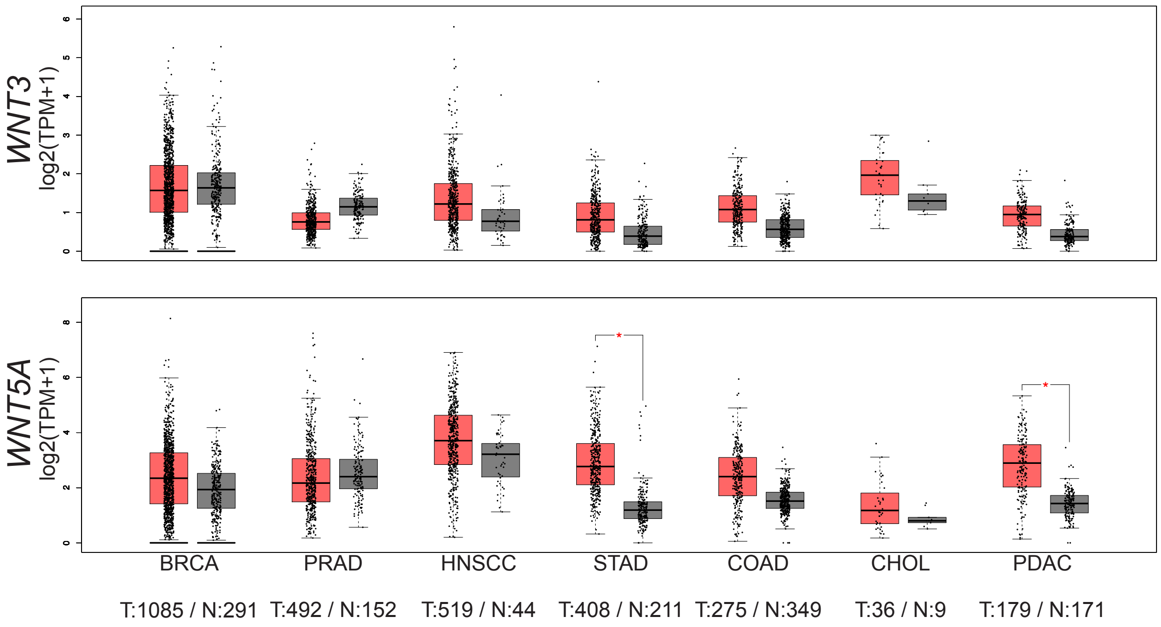

Supplementary Figure 18: Box plots from GEPIA gene expression data comparing the expression of selected axon guidance genes in tumor tissue and normal tissues in several cancers associated with high nerve density and PNI. Comparison of mRNA expression of axon guidance genes in TCGA tumors (red) and TCGA and GTEx normal samples (gray). mRNA expression is presented as log2(TPM+1). The analysis is presented for BRCA (1085 tumor samples and 291 normal samples), PRCA (492 tumor samples and 152 normal samples), HNSCC (519 tumor samples and 44 normal samples), STAD (408 tumor samples and 211 normal samples), COAD (275 tumor samples and 349 normal samples), CHOL (36 tumor samples and 9 normal samples) and PDAC (179 tumor samples and 171 normal samples). The following genes were analyzed *BOC*, *CXCL12*, *ENDRA*, *EFNA1*, *EFNA4*, *EFNA5*, *EFNB1*, *EFNB2*, *EPHA1*, *EPHA2*, *EPHA3*, *EPHA4*, *EPHA5*, *EPHB2*, *EPHB6*, *FLRT2*, *FLRT3*, *FOXD1*, *FOXG1*, *LAMA2*, *LAMA3*, *LAMA5*, *LAMB2*, *LAMC2*, *PLXNA1*, *PLXNA2*, *PLXNB2*, *PLXNC1*, *PLXND1*, *RAC2*, *ROBO1*, *ROBO2*, *ROBO4*, *SEMA3A*, *SEMA3C*, *SEMA3D*, *SEMA3G*, *SEMA4B*, *SEMA5B*, *SEMA6A*, *SEMA6D*, *SEMA7A*, *SLC7A11*, *SLIT2*, *SLIT3*, *TRIO*, *UNC5B*, *UNC5C*, *WNT3*, *WNT5A*. Statistical significance was assessed by one-way ANOVA test performed on the GEPIA platform. Abbreviations: BRCA, breast cancer; PRCA, prostate cancer; HNSCC, head and neck squamous cell carcinoma ; STAD, stomach adenocarcinoma; COAD, colon adenocarcinoma; CHOL, cholangiocarcinoma; PDAC, pancreatic ductal adenocarcinoma; TPM, transcripts per million; T, tumor and N, normal.

Supplementary figure 19

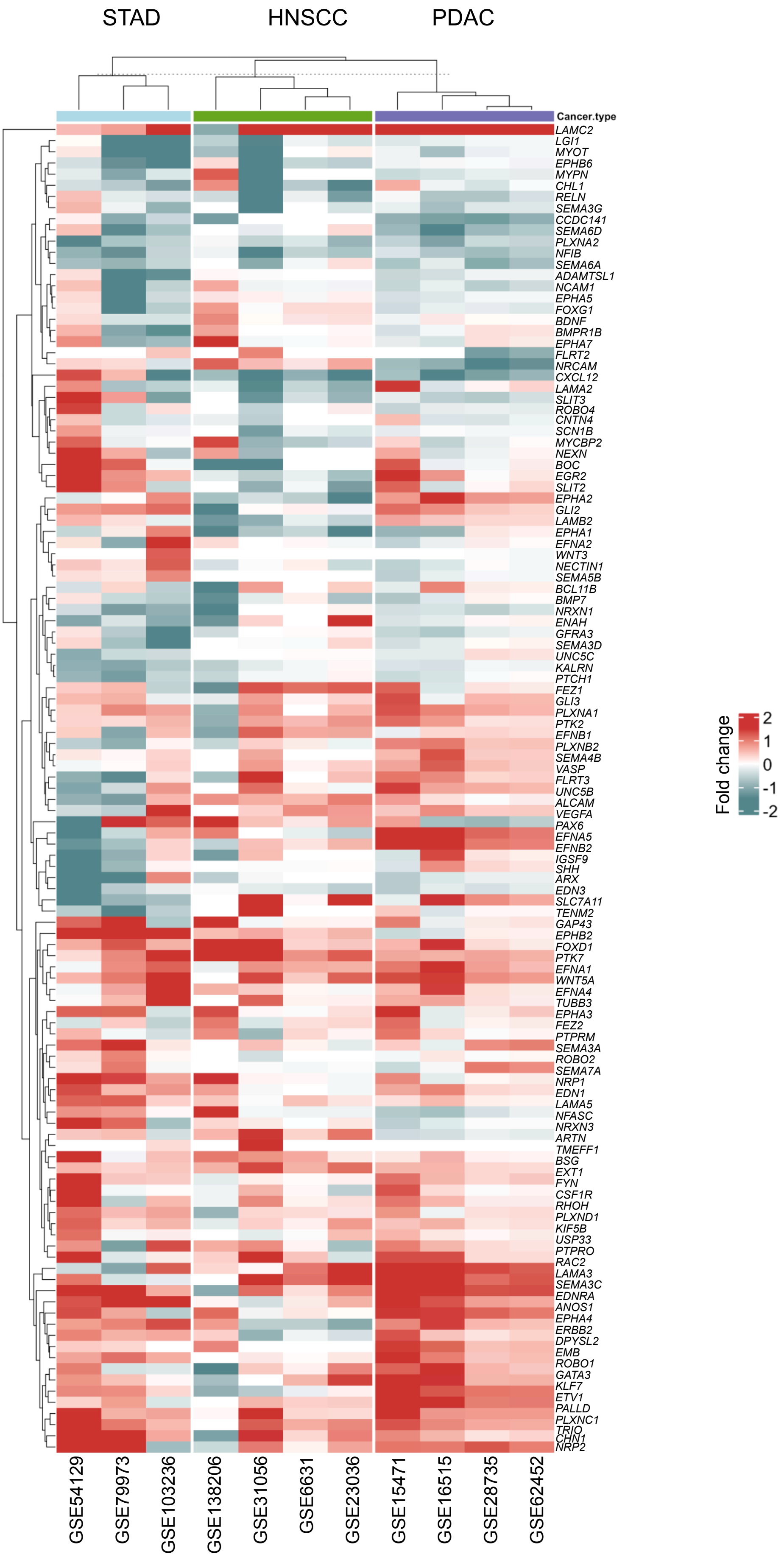

Supplementary Figure 19: Axon guidance differentially expressed genes in head and neck squamous cell carcinoma (HNSCC), pancreatic ductal adenocarcinoma (PDAC) and stomach adenocarcinoma (STAD). A hierarchical clustering heatmap of DEGs from all GEO datasets analyzed were cross referenced to the axon guidance gene signature (GO:0007411) (**Supplementary Table 9**). STAD datasets GSE33651 and GSE65801 are not depicted since the data expression for most of axon guidance probes were not available. Here, DEGs were defined as genes with |log2FC|>1 and adj. p-val <0.05. Abbreviations: DEG, differentially expressed gene; HNSCC, head and neck squamous cell carcinoma; PDAC, pancreatic ductal adenocarcinoma; STAD, stomach adenocarcinoma; FC, fold change and adj. p-value, adjusted p value.

Supplementary Figure 20

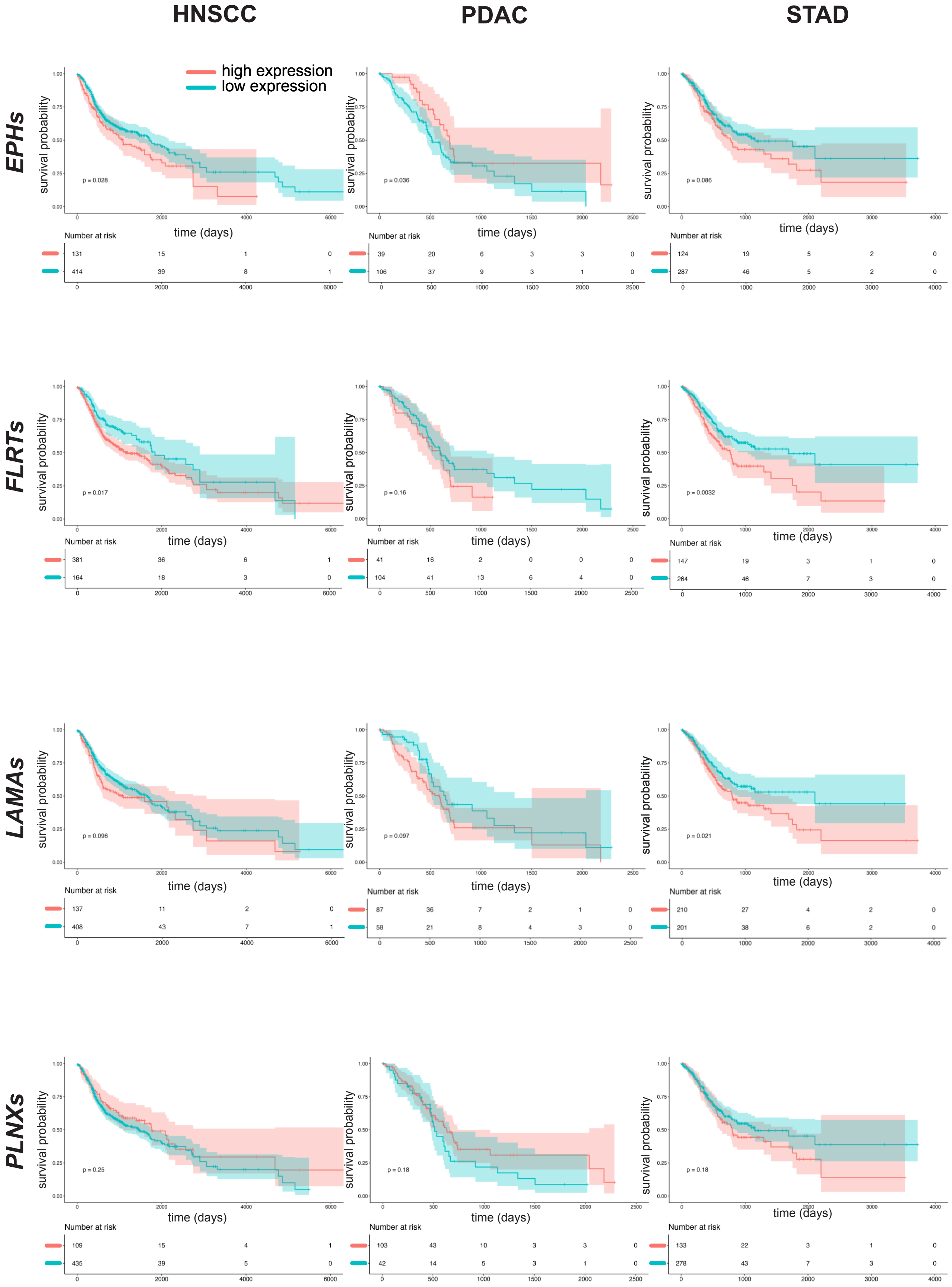

Supplementary Figure 20 continued

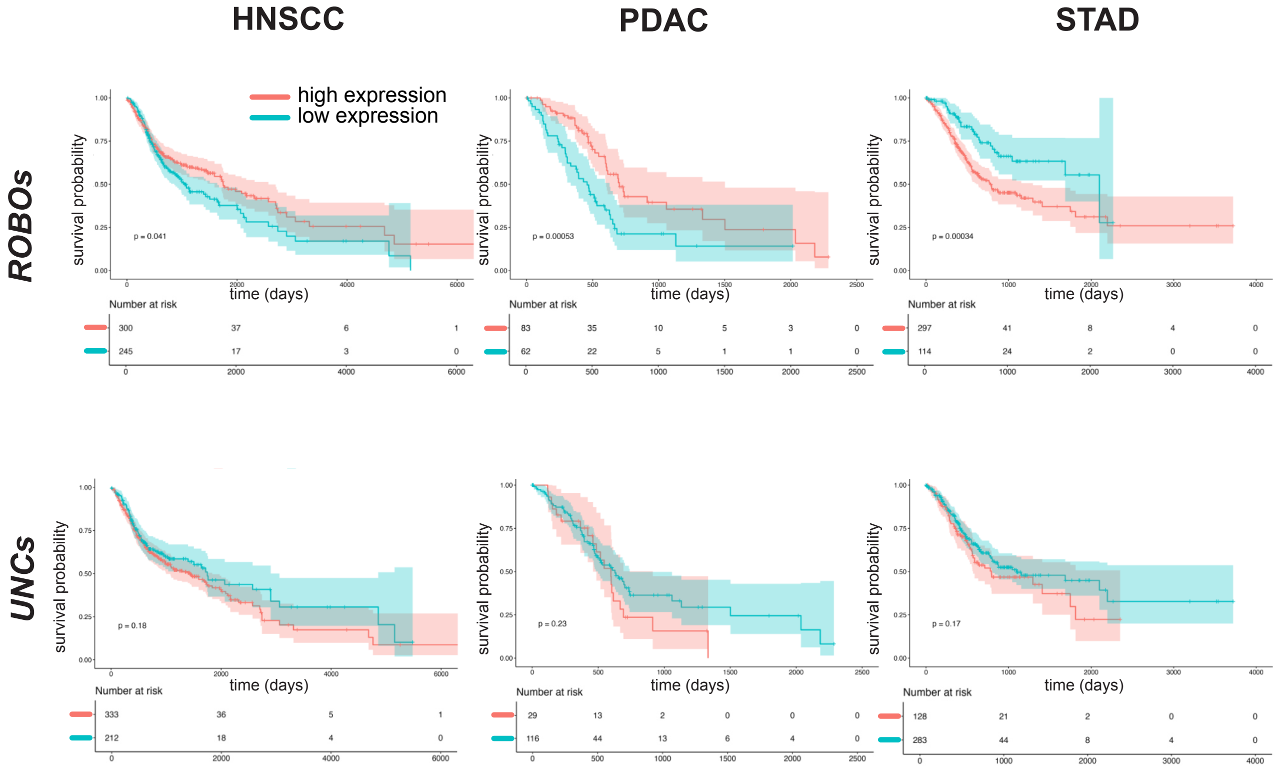

Supplementary Figure 20: Survival analysis of axon guidance gene families analyzing cohorts of paralogue genes. Kaplan-Meier plots are shown which visualize HNSCC, PDAC and STAD overall survival based on combined expression levels for the following paralogue gene families: EPH receptors (*EPHA1*, *EPHA10*, *EPHA2*, *EPHA3*, *EPHA4*, *EPHA5*, *EPHA6*, *EPHA7*, *EPHA8*, *EPHB1*, *EPHB2*, *EPHB3*, *EPHB4*, *EPHB6*), FLRTs (*FLRT2*, *FLRT3*), laminins (*LAMA1*, *LAMA2*, *LAMA3*, *LAMA5*, *LAMB2*, *LAMC2*), plexins (*PLXNA1*, *PLXNA2*, *PLXNA3*, *PLXNA4*, *PLXNA4B*, *PLXNB1*, *PLXNB2*, *PLXNB3*, *PLXNC1*, *PLXND1*), ROBO receptors (*ROBO1*, *ROBO2*, *ROBO3*, *ROBO4*) and UNCs (*UNC5A*, *UNC5B*, *UNC5C*, *UNC5D*). For this analysis, patients were segregated in two cohorts (low and high gene expression) based on the most significant cut-off value for the combined genes. Low and high expression groups are depicted in blue and red curves, respectively. Abbreviations: HNSCC, head and neck squamous cell carcinoma ; PDAC, pancreatic ductal adenocarcinoma; ns, not significant. STAD, stomach adenocarcinoma.

2 Supplementary table captions

Supplementary Table 1: Differentially expressed genes in head and neck squamous cell carcinoma (HNSCC), pancreatic ductal adenocarcinoma (PDAC) and stomach adenocarcinoma (STAD) GEO datasets. The DEGs listed are defined as genes with |log2FC|>1 and adj. p-val <0.05 from all GEO databases examined for HNSCC (sheet 1), PDAC (sheet 2) and STAD (sheet 3). Data is organized by columns for each dataset showing the dataset identification number, gene symbol, log2FC and adj. p-val for each dataset. Abbreviations: DEG, differentially expressed gene; HNSCC, head and neck squamous cell carcinoma; PDAC, pancreatic ductal adenocarcinoma; STAD, stomach adenocarcinoma; FC, fold change.

Supplementary Table 2: Data from Figure 1A - Intersection analysis of differentially expressed genes identified from head and neck squamous cell carcinoma (HNSCC), pancreatic ductal adenocarcinoma (PDAC) and stomach adenocarcinoma (STAD) GEO datasets. For each individual cancer type, DEGs identified from the GEO datasets listed in **Table 1** were compared and intersection analysis was used to identify common genes between datasets. The datasets with common genes are presented in column A, followed by the number of genes (column B) which are then listed in column C with gene symbols. Each row in the first column shows a different combination of datasets. Abbreviations: DEG, differentially expressed gene; HNSCC, head and neck squamous cell carcinoma ; PDAC, pancreatic ductal adenocarcinoma; STAD, stomach adenocarcinoma.

Supplementary Table 3: Data from Figure 2G - DEGs common in 3 or more datasets per cancer type for head and neck squamous cell carcinoma (HNSCC), pancreatic ductal adenocarcinoma (PDAC) and stomach adenocarcinoma (STAD) GEO datasets. DEGs identified in more than 3 datasets for each cancer type examined were intersected amongst all cancer types examined. The type of cancers with common genes are presented in column A, followed by the number of genes (column B) which are listed in column C with gene symbols. Each row in the first column shows a different combination of datasets. Abbreviations: DEG, differentially expressed gene; HNSCC, head and neck squamous cell carcinoma; PDAC, pancreatic ductal adenocarcinoma; STAD, stomach adenocarcinoma.

Supplementary Table 4: List of genes in the neurodevelopmental gene signature used in this study. The list of 2193 genes for the neurodevelopmental signature annotated for nervous system development GO:0007399 used in this study which was constructed in the QuickGo repository.

Supplementary Table 5: Data from Figure 3. Intersection analysis of the neurodevelopmental gene signature versus PNI gene lists in Figures 3A and 3B and the intersection of DEGS between each individual GEO cohort used for head and neck squamous cell carcinoma (HNSCC), pancreatic ductal adenocarcinoma (PDAC) and stomach adenocarcinoma (STAD). The neurodevelopmental signature in **Supplementary Table 4** (GO: 0007399) was cross-referenced with different data cohorts. The list of genes (gene symbols shown) from the intersection analysis is shown

as follows: In sheet 1 and summarized as a Venn diagram in **Figure 3A** the stomach adenocarcinoma PNI list from Jia *et al* 2019 (Jia et al., 2019). This is shown as an intersection of genes common to both the Jia gastric cancer PNI list and the neurodevelopmental list, genes only in the neurodevelopmental list and not the Jia gastric cancer PNI list and genes only in Jia gastric cancer PNI list and not the neurodevelopmental list. In sheet 2 and summarized as a Venn diagram in **Figure 3B** the head and neck squamous cell carcinoma PNI list from Eviston *et al* 2021 (Eviston et al., 2021). This is shown as an intersection of genes common to both the Eviston HNSCC PNI list and the neurodevelopmental list, genes only in the neurodevelopmental list and not in the Eviston HNSCC PNI list and genes only in the Eviston HNSCC PNI list and not the neurodevelopmental list. In sheets 3 – 5 and the Venn diagrams shown in **Figure 3C** the DEGs identified in the GEO cohorts for HNSCC datasets GSE6631, GSE23036, GSE31056 and GSE138206 (**Figure 2D**) crossed with the neurodevelopmental signature (Sheet 3), PDAC datasets GSE15471, GSE16515, GSE28735 and GSE62452 (**Figure 2E**) crossed with the neurodevelopmental signature (Sheet 4) and STAD datasets GSE33651, GSE54129, GSE65801, GSE79973 and GSE103236 (**Figure 2F**) crossed with the neurodevelopmental signature (Sheet 5). Abbreviations: PNI, perineural invasion; DEG, differentially expressed gene; HNSCC, head and neck squamous cell carcinoma ; PDAC, pancreatic ductal adenocarcinoma; STAD, stomach adenocarcinoma, FC, fold change; adj. p-val, adjusted p-value.

Supplementary Table 6: Data from Figure 3C – The intersection of the neurodevelopmental gene list and common head and neck squamous cell carcinoma (HNSCC), pancreatic ductal adenocarcinoma (PDAC) and stomach adenocarcinoma (STAD) GEO datasets. This Table lists the common neurodevelopmental DEGs identified in PDAC, HNSCC and STAD GEO datasets. In column 1 the cancer types with common neurodevelopmental genes is shown, followed by the number of common genes between the cancers identified in column 1 (column 2) and in column 3 the genes are listed by gene symbol. There are several rows in column 1 each showing the intersection between different cancers, STAD HNSCC PDAC, STAD PDAC, STAD HNSCC , HNSCC PDAC, STAD, PDAC and HNSCC. A total of 126 neurodevelopmental genes were found to be common between HNSCC, PDAC and STAD. Abbreviations: HNSCC, Head and Neck Squamous Cell Carcinoma; PDAC, pancreatic ductal adenocarcinoma; STAD, stomach adenocarcinoma.

Supplementary Table 7: Gene expression profile characteristics of datasets which segregate patients sample with or without PNI. Microarray (GEO) datasets with available PNI status for cancers analyzed in this study. For HNSCC, dataset GSE86544 consisted in 15 tumors with PNI and 9 tumors without PNI, and for PDAC dataset GSE102238 consisted in 28 tumors with PNI and 22 tumors without PNI. Abbreviations: PNI, perineural invasion; GEO, Gene Expression Omnibus database; HNSCC, head and neck squamous cell carcinoma ; PDAC, pancreatic ductal adenocarcinoma.

Supplementary Table 8: Differentially expressed genes in head and neck squamous cell carcinoma (HNSCC) PNI dataset GSE86544. List of DEGs defined as genes with |logFC|>1 and adj. p-val <0.05 when comparing tumors with PNI with tumors without PNI. Gene symbols and their corresponding logFC and adj. p-val are depicted. Abbreviations: HNSCC, head and neck squamous cell carcinoma, FC, fold change; adj. p-val, adjusted p-value.

Supplementary Table 9: List of genes in the Axon guidance gene signature used in this study. Gene signature list of 281 genes annotated for axon guidance GO:0007411 constructed in the QuickGo repository is shown. Gene symbols are listed.

Supplementary Table 10: Axon guidance DEGs identified in in head and neck squamous cell carcinoma (HNSCC), pancreatic ductal adenocarcinoma (PDAC) and stomach adenocarcinoma (STAD) GEO datasets. List of DEGs from PDAC, HNSCC and STAD from the GEO datasets in **Supplementary Table 1** which were cross-referenced with the axon guidance gene signature from **Supplementary Table 9** (GO:007411). Gene symbols are listed. A total of 50 axon guidance genes were found in HNSCC, 58 in PDAC and 79 in STAD. Abbreviations: DEG, differentially expressed gene; HNSCC, head and neck squamous cell carcinoma ; PDAC, pancreatic ductal adenocarcinoma; STAD, stomach adenocarcinoma.

Supplementary Table 11: Dysregulation of axon guidance genes in different cancer types from TCGA data cohorts. Genes from the axon guidance signature (GO:0007411) were analyzed for their expression in tumor samples and normal samples from the TCGA and GTEx cohorts. Gene symbols are listed. DEGs were defined as genes with |log2FC|>1 and adj. p-val <0.01. Genes in red and blue are significantly upregulated and downregulated, respectively. Genes in gray did not show significant differences. Genes are arranged in alphabetical order. Abbreviations: HNSCC, Head and Neck Squamous Cell Carcinoma; PDAC, pancreatic ductal adenocarcinoma; STAD, stomach adenocarcinoma; BRCA, breast cancer; PRCA, prostate cancer; HNSCC, head and neck squamous cell carcinoma ; STAD, stomach cancer; COAD, colon adenocarcinoma; CHOL, cholangiocarcinoma; PAAD, pancreatic adenocarcinoma.

Supplementary Table 12: Survival analysis of cancer patients with respect to the expression of axonal guidance genes. Hazard ratios (HR) and 95% confidence interval (CI) are depicted for axonal guidance genes belonging to gene families commonly dysregulated in PDAC, HNSCC and STAD. Hazard ratios were calculated by comparing high mRNA expression to low mRNA expression cohorts using the best cut-off value. Genes with HR>1 and logrank p <0.05 confer worse overall survival when highly expressed, whereas genes with HR<1 and logrank p<0.05 confer better overall survival when

highly expressed. Abbreviations: HNSCC, head and neck squamous cell carcinoma ; PDAC, pancreatic ductal adenocarcinoma; STAD, stomach adenocarcinoma; HR, hazard ratio; CI, confidence interval.

3 Script for the equalization analysis (used in Supplementary Figures 1 – 5)

In this script a function performing the differential analysis by the limma package used by GEO2R was used for comparing a randomly selected samples from the bigger group (tumor or control) versus the smallest group (tumor or control).

```
# Libraries loading

library(GEOquery)

library(limma)


# Function

perform_analysis <- function(gset_subset, cont_matrix, design_subset) {

  # Group membership for all samples

  gsms <- "1111111111111111111100000000000000000000"

  sml <- strsplit(gsms, split = "")[[1]]


  # sample assignment and design matrix

  gs <- factor(sml)

  groups <- factor(as.character(gs), levels = c("0", "1"))

  levels(groups) <- c("Normal", "DMD")


  # Log2 transformation if needed

  qx <- as.numeric(quantile(gset_subset, c(0., 0.25, 0.5, 0.75, 0.99, 1.0), na.rm = TRUE))
```

```
  LogC <- (qx[5] > 100) || (qx[6] - qx[1] > 50 && qx[2] > 0)
  if (LogC) {
    gset_subset[gset_subset <= 0] <- NaN
    gset_subset <- log2(gset_subset)
  }

  # data dimensions matching
  if (nrow(design_subset) != ncol(gset_subset)) {
    stop("Error: The number of rows in the design matrix does not match the number of samples in the data object.")
  }

  # Fit linear model
  fit <- lmFit(gset_subset, design_subset)

  # Model coefficients
  fit2 <- contrasts.fit(fit, cont_matrix)

  # table for the top genes
  fit2 <- eBayes(fit2, 0.01)
  #tT <- topTable(fit2, adjust = "fdr", sort.by = "B", number = 250)
  tT <- topTable(fit2, adjust = "fdr", sort.by = "B", number=Inf)



  return(tT)
}
```

```
# GEO loading

gset <- getGEO("GSE54129", GSEMatrix = TRUE, AnnotGPL = TRUE)

if (length(gset) > 1) idx <- grep("GPL571", attr(gset, "names")) else idx <- 1

gset <- gset[[idx]]


fvarLabels(gset) <- make.names(fvarLabels(gset))


# sample assignment 0=normal 1: tumor

gsms <-
"0000000000000000000001111111111111111111111111111111111111111111111111111111111
1111111111111111111111111111111111111111111111111111"

sml <- strsplit(gsms, split = "")[[1]]


# sample assignment and design matrix

gs <- factor(sml)

groups <- factor(as.character(gs), levels = c("0", "1"))

levels(groups) <- c("Normal", "DMD")

gset$group <- groups


# Contrasts of interest

cts <- paste(levels(groups)[1], "-", levels(groups)[2], sep = "")

design <- model.matrix(~ group + 0, data = gset)

colnames(design) <- levels(groups)

cont_matrix <- makeContrasts(contrasts = cts, levels = design)
```

```
##################################################

dir_path <- "path where results from below loop will be stored"


# Loop with N number of repetitions
N <- 100
results <- list()
sample_tables <- list()
analysis_tables <- list()


for (i in 1:N) {
  # # Select size:X random samples from group "1" (DMD)
  tumor_samples <- sample(colnames(gset)[groups == levels(groups)[2]], size = 21)
  #
  # # Select all samples from group "0" (Normal)
  normal_samples <- colnames(gset)[groups == levels(groups)[1]]

  # #select all the tumors
  # tumor_samples <- colnames(gset)[groups == levels(groups)[2]]
  # #select random controls
  # normal_samples <- sample(colnames(gset)[groups == levels(groups)[1]], size = 23)

  # Selected samples combined
```

```
selected_samples <- c(tumor_samples, normal_samples)

# Subset gset for the selected samples
gset_subset <- exprs(gset[, selected_samples])
  rownames(gset_subset) <- featureNames(gset)

# keep gset_subset as matrix
if (!is.matrix(gset_subset)) {
  gset_subset <- as.matrix(gset_subset)
}

design_subset <- design[selected_samples, , drop = FALSE]
colnames(design_subset) <- c("Normal", "DMD")
cont_matrix <- makeContrasts("DMD - Normal", levels = design_subset)

# call the function
analysis_results <- perform_analysis(gset_subset, cont_matrix, design_subset)
analysis_results$Probe <- rownames(analysis_results)
analysis_results <- analysis_results[, c("Probe", "adj.P.Val", "P.Value", "t", "B", "logFC")]

# sace the randomized samples
sample_table <- data.frame(Randomly_Selected_Samples = tumor_samples)
sample_tables[[i]] <- sample_table
analysis_tables[[i]] <- analysis_results
```

```
}

# export the data

for (i in 1:N) {

  write.table(sample_tables[[i]], file.path(dir_path, paste0("Sample_Table_", i, ".txt")), sep = "\t", quote = FALSE, row.names = FALSE)

}

for (i in 1:N) {

  write.table(analysis_tables[[i]], file.path(dir_path, paste0("Analysis_Results_", i, ".txt")), sep = "\t", quote = FALSE, row.names = FALSE)

}
```

4 Script for the gene cohort analysis (used in Figure 7 and Supplementary Figure 20)

Before computing the script, gene expression matrix and survival data were downloaded from the Xena repository https://xenabrowser.net. Both matrix names were changed in the script (highlighted text).

- "Gene_Expression <- fread("For_LuzMa/Gene_Expression_PDAC_Only_140622.txt", check.names = F) Gene_Expression <- Gene_Expression %>%"
- "Survival_data <- fread("For_LuzMa/Survival_Data_Xenobrowser_140622.txt")"

To follow the script a text file (e.g., UNC.txt) was created with the list of genes to be included in the gene cohort analysis and the name was changed in the script for each gene list (see highlighted text).

Once the percentiles and p-values were calculated, the best percentile was defined as the percentile with the most significant value (e.g., 0.3) and used as cut-off value for constructing the survival plot.

````
```{r setup, include=FALSE}

knitr::opts_chunk$set(echo = TRUE)

```

```{r}

library(data.table)

library(tidyverse)
````
````

~~~
library("survminer")

require("survival")

```

```{r}

Gene_Expression <- fread("For_LuzMa/Gene_Expression_PDAC_Only_140622.txt", check.names =
F)

Gene_Expression <- Gene_Expression %>%

  column_to_rownames("V1")

```

```{r}

Survival_data <- fread("For_LuzMa/Survival_Data_Xenobrowser_140622.txt")

```

```{r}

Sample.Info <- fread("For_LuzMa/Sample.Info.PDAC.Only.140622.txt")

```

#Make function for finding gene signature z score

```{r}

get_sign_mean_z<-function(df, gene_sign){

  library(tidyverse)

  # subsets the data frame for the genes in the signature

  sig_df<-df %>%

    rownames_to_column("Gene") %>%

    filter(Gene %in% gene_sign) %>%

    column_to_rownames("Gene")
~~~

~~~
  # calculates the z-scores on the subsetted matrix
  sig_df_z<-sig_df %>%
    t() %>%
    scale() %>%
    t() %>%
    as.data.frame() %>%
    drop_na()
 # takes the mean z-score
  mean_z<-colMeans(sig_df_z)
  return(mean_z)
}
```
#A function that takes the data and the pc cutoff, then returns a p value
```{r}
get_gene_sig_p_val <-function(in_gene_file, df, pc, Survival_data){
  library(dplyr)
  library(data.table)
Gene_sign_custom<-fread(in_gene_file, header = FALSE) %>% `$`(V1)
  custom_z_score <- get_sign_mean_z(df, Gene_sign_custom)
  Top.patients<-(custom_z_score>quantile(custom_z_score, pc)) %>%
    `[`(.==TRUE) %>%
    names()
  Bottom.patients<-(custom_z_score<quantile(custom_z_score, pc)) %>%
    `[`(.==TRUE) %>%
    names()
  Bottom.patients.survival <- Survival_data %>%
~~~

```
    filter(sample %in% Bottom.patients) %>%
    mutate(Expression.Group = "Low")
  Top.patients.survival <- Survival_data %>%
    filter(sample %in% Top.patients) %>%
    mutate(Expression.Group = "High")
  Patients.survival <- bind_rows(Top.patients.survival, Bottom.patients.survival) %>%
    mutate(OS = as.numeric(OS)) %>%
    mutate(OS.time = as.numeric(OS.time))
  library("survminer")
  require("survival")
  surv_object <- Surv(time = Patients.survival$OS.time, event = Patients.survival$OS)
  fit.coxph <- coxph(surv_object ~ Expression.Group, data = Patients.survival)
  pval <- summary(fit.coxph)$sctest[3]
  return(pval)
}
```

```{r}
get_surv_plot <-function(in_gene_file, in_df, in_pc, in_Survival_data){
  Gene_sign_custom<-fread(in_gene_file, header = FALSE) %>% `$`(V1)
  custom_z_score <- get_sign_mean_z(in_df, Gene_sign_custom)
  Top.patients<-(custom_z_score>quantile(custom_z_score, in_pc)) %>%
    `[`(.==TRUE) %>%
    names()
  Bottom.patients<-(custom_z_score<quantile(custom_z_score, in_pc)) %>%
```

```
    `[`(.==TRUE) %>%
    names()

  Bottom.patients.survival <- in_Survival_data %>%
    filter(sample %in% Bottom.patients) %>%
    mutate(Expression.Group = "Low")
  Top.patients.survival <- in_Survival_data %>%
    filter(sample %in% Top.patients) %>%
    mutate(Expression.Group = "High")
  Patients.survival <- bind_rows(Top.patients.survival, Bottom.patients.survival) %>%
    mutate(OS = as.numeric(OS)) %>%
    mutate(OS.time = as.numeric(OS.time))
  library("survminer")
  require("survival")
  surv_object <- Surv(time = Patients.survival$OS.time, event = Patients.survival$OS)
  assign("surv_object", surv_object, envir = .GlobalEnv)
  fit1 <- survfit(surv_object~Expression.Group, data = Patients.survival)
  plot <- ggsurvplot((fit1), data = Patients.survival, pval = TRUE)
  return(plot)
}
```

#Find best percentile

```{r}
in_gene_file <- "UNC.txt"
in_df <- Gene_Expression
in_Survival_data <- Survival_data
```

```
pcs <- seq(0.2, 0.8, by = 0.01)
pvals <- vector("double", length(pcs))
for (i in seq_along(pcs)){
in_pc <- pcs[[i]]
pvals[[i]] <- get_gene_sig_p_val(in_gene_file, in_df, in_pc, in_Survival_data)
}
Best.pc <- as.data.frame(pcs)
pcals <- as.data.frame(pvals)
Best.pc <- cbind(Best.pc, pvals)
```

```{r}
gene_file <- "UNC.txt"
df <- Gene_Expression
Survival_data <- Survival_data
pc <- 0.30
get_surv_plot(gene_file, df, pc, Survival_data)
```

5 Supplementary references

Eviston, T. J., Minaei, E., Mueller, S. A., Ahmadi, N., Ashford, B., Clark, J. R., et al. (2021). Gene expression profiling of perineural invasion in head and neck cutaneous squamous cell carcinoma. *Sci. Rep.* 11, 13192. doi: 10.1038/s41598-021-92335-4.

Jia, X., Lu, M., Rui, C., and Xiao, Y. (2019). Consensus-Expressed CXCL8 and MMP9 Identified by Meta-Analyzed Perineural Invasion Gene Signature in Gastric Cancer Microarray Data. *Front. Genet.* 10, 851. doi: 10.3389/fgene.2019.00851.
